# Prediction of Th1 and Cytotoxic T Lymphocyte Epitopes of *Mycobacterium tuberculosis* and Evaluation of Their Potential in the Diagnosis of Tuberculosis in a Mouse Model and in Humans

**DOI:** 10.1128/spectrum.01438-22

**Published:** 2022-08-08

**Authors:** Wenping Gong, Yan Liang, Jie Wang, Yinping Liu, Yong Xue, Jie Mi, Pengchuan Li, Xiaoou Wang, Lan Wang, Xueqiong Wu

**Affiliations:** a Tuberculosis Prevention and Control Key Laboratory/Beijing Key Laboratory of New Techniques of Tuberculosis Diagnosis and Treatment, Senior Department of Tuberculosis, The Eighth Medical Center of PLA General Hospital, Beijing, China; Quest Diagnostics Nichols Institute

**Keywords:** active tuberculosis, ATB, biomarkers, differential diagnosis, immunodominant peptides, latent tuberculosis infection, LTBI, *Mycobacterium tuberculosis*

## Abstract

Latent tuberculosis infection (LTBI) is the primary source of tuberculosis (TB) but there is no suitable detection method to distinguish LTBI from active tuberculosis (ATB). In this study, five antigens of Mycobacterium tuberculosis belonging to LTBI and regions of difference (RDs) were selected to predict Th1 and cytotoxic T lymphocyte (CTL) epitopes. The immunodominant Th1 and CTL peptides were identified in mouse models, and their performance in distinguishing LTBI from ATB was determined in mice and humans. Ten Th1 and ten CTL immunodominant peptides were predicted and synthesized *in vitro*. The enzyme-linked immunosorbent spot assay results showed that the combination of five Th1 peptides (area under the curve [AUC] = 1, *P < *0.0001; sensitivity = 100% and specificity = 93.33%), the combination of seven CTL peptides (AUC = 1, *P < *0.0001; 100 and 95.24%), and the combination of four peptide pools (AUC = 1, *P < *0.0001; sensitivity = 100% and specificity = 91.67%) could significantly discriminate mice with LTBI from mice with ATB or uninfected controls (UCs). The combined peptides or peptide pools induced significantly different cytokine levels between the three groups, improving their ability to differentiate ATB from LTBI. Furthermore, it was found that pool 2 could distinguish patients with ATB from UCs (AUC = 0.6728, *P = *0.0041; sensitivity = 72.58% and specificity = 59.46%). The combination of Th1 and CTL immunodominant peptides derived from LTBI-RD antigens might be a promising strategy for diagnosing ATB and LTBI in mice and patients with ATB and uninfected controls.

**IMPORTANCE** Latent tuberculosis infection (LTBI) is a challenging problem in preventing, diagnosing, and treating tuberculosis (TB). The innate and adaptive immune responses are essential for eliminating or killing the mycobacteria. Antigen-presenting cells (APCs) present and display mycobacterium peptides on their surfaces, and recognition between T cells and APCs is based on some essential peptides rather than the full-length protein. Therefore, the selection of candidate antigens and the prediction and screening of potential immunodominant peptides have become a key to designing a new generation of TB diagnostic biomarkers. This study is the first to report that the combination of Th1 and CTL immunodominant peptides derived from LTBI-RD antigens can distinguish LTBI from active TB (ATB) in animals and ATB patients from uninfected individuals. These findings provide a novel insight for discovering potential biomarkers for the differential diagnosis of ATB and LTBI in the future.

## INTRODUCTION

Tuberculosis (TB) has become one of the TOP 10 major infectious diseases in humans. According to the World Health Organization (WHO) 2021 Global Tuberculosis Report, there were 10 million new TB cases and 1.3 million deaths worldwide in 2020 (1). Previous studies have shown that about one-third of the world’s population has been infected with TB, but only 10% develop active TB, meaning that nearly 90% of people experience latent tuberculosis infection (LTBI) (2, 3). LTBI is a particular state in which an individual is infected with Mycobacterium tuberculosis that has not yet developed to active TB (ATB), characterized by a positive tuberculin skin test (TST) without clinical manifestations and imaging changes of ATB. It is estimated that individuals with LTBI have a 5 to 10% lifetime risk of developing ATB without timely diagnosis and intervention, and this risk can be as high as 10% per year if they are coinfected with human immunodeficiency virus (HIV), a risk much higher than that for HIV-negative people (4–6). Epidemiological investigations have shown that about 85 to 90% of newly diagnosed active pulmonary TB developed from LTBI (7). Therefore, early detection and diagnosis of individuals with LTBI is the basis for controlling the spread of TB.

Currently, diagnostic methods for LTBI include the TST and newly appearing interferon gamma (IFN-γ) release assays (IGRAs) (8). The antigen used in the traditional TST is old tuberculin or purified protein derivative (PPD), resulting in high false-positive rates in the population with BCG vaccination and is unable to distinguish between patients with LTBI and ATB (9). Recently, several new TST assays have been developed, such as the Diaskintest, C-Tb skin test, and EC-Test, which replaced traditional PPD with early secreted antigenic target 6 (ESAT6) and culture filtrate protein 10 (CFP10) antigens of the M. tuberculosis virulent strain (10, 11). Similarly, five commercialized IGRA kits have been developed to overcome the deficiencies of the traditional TST, such as the T-cell spot of the TB assay (T-SPOT.TB), QuantiFERON-TB Gold In-Tube (QFT-GIT), QuantiFERON-TB Gold-Plus (QFT-Plus), LIAISON QuantiFERON-TB Gold Plus (LIAISON QFT-Plus), and LIOFeron TB/LTBI (12–15). Coincidentally, these improved TST methods and the latest IGRAs share the same antigens, CFP10 and ESAT6, as stimulants. Both antigens are absent in the BCG strain and most nontuberculous mycobacteria. Therefore, these methods have significantly enhanced the sensitivity and specificity of diagnosis of M. tuberculosis infection, but they cannot differentiate LTBI from ATB.

Thus, discovering novel and efficient diagnostic biomarkers for LTBI can improve the sensitivity and specificity of LTBI diagnosis, reduce the incidence of M. tuberculosis infection, and eliminate potential sources of infection, which is of great significance for the control and prevention of TB. Previous studies have suggested that antigens of M. tuberculosis belonging to both mycobacterial regions of difference (RDs) and latent infection are the most promising candidates for LTBI differential diagnosis (7, 16, 17). In our previous study, we have identified 21 candidate antigens related to LTBI and RD (LTBI-RD), including Rv1511, Rv1736c, Rv1737c, Rv1978, Rv1980c, Rv1981c, Rv2031c, Rv2626c, Rv2653c, Rv2654c, Rv2656c, Rv2657c, Rv2658c, Rv2659c, Rv2660c, Rv3425, Rv3429, Rv3872, Rv3873, Rv3878, and Rv3879c (7). In the present study, we selected five antigens (Rv1737c, Rv2031c, Rv2626c, Rv2659c, and Rv2660c) from these 21 candidates to predict potential epitopes recognized with T helper 1 cell (Th1) and cytotoxic T lymphocytes (CTL) by using bioinformatics technologies. The predicted immunodominant Th1 and CTL peptides were synthesized *in vitro*. Their potential ability to distinguish LTBI from ATB was evaluated in animal models using the enzyme-linked immunosorbent spot (ELISpot) assay and high-throughput liquid protein microarray detection technology. We also explored five peptide pools’ potential ability to distinguish patients with ATB and LTBI individuals using the ELISpot assay. Furthermore, the sensitivity and specificity of these peptides and their communal pools were confirmed by using the receiver operator characteristics (ROC) curve. This study provides new candidate biomarkers for the differential diagnosis of LTBI and ATB. It highlights the potential value of the peptides derived from LTBI-RD antigens as a new method for diagnosing LTBI and ATB.

## RESULTS

### Twelve major MHC-I alleles and 25 MHC-II alleles were identified with high frequency in the Chinese population.

By utilizing the Allele Frequency Net Database, we identified 12 high-frequency histocompatibility complex I (MHC-I) alleles with an allele frequency of ≥0.10 in the Chinese population, including five HLA-A alleles, three HLA-B alleles, and four HLA-C alleles (see Table S1 in the supplemental material). In addition, we also identified 25 high-frequency MHC-II alleles with an allele frequency of ≥0.10 in the Chinese population, including 13 DRB1 alleles, 8 DQA1/DQB1 alleles, and 4 DPA1/DPB1 alleles (see Table S1).

### Seventy Th1 and 49 CTL dominant epitopes were predicted from the five candidate antigens.

The Immune Epitope Database (IEDB; http://tools.iedb.org/mhcii/ or http://tools.immuneepitope.org/mhci/) was used to predict the Chinese population’s predominant Th1 and CTL epitopes. The epitopes predicted by the IEDB database were ranked from small to large according to the comprehensive percentile rank score (the smaller the score, the higher the affinity). Epitopes with a percentile rank score of ≤10 were selected as dominant epitopes, and a total of 70 Th1 (see Table S2) and 49 CTL (see Table S3) dominant epitopes were obtained from five candidate antigens, respectively. In brief, 20 Th1 and 12 CTL dominant epitopes were predicted from Rv1737c antigen, 13 Th1, and 10 CTL dominant epitopes were predicted from Rv2031c antigen, 14 Th1 and 10 CTL dominant epitopes were predicted from Rv2626c antigen, 17 Th1 and 10 CTL dominant epitopes were predicted from Rv2659c antigen, and 6 Th1 and 7 CTL dominant epitopes were predicted from Rv2660c antigen, respectively (see Table S2 and Table S3). Among them, 10 Th1 and 10 CTL peptides with the highest ranking which could be synthesized artificially *in vitro* were selected as final dominant peptides for the following experiments (Table 1), including Th1-Rv1737c-P3, Th1-Rv1737c-P5, Th1-Rv2031c-P1, Th1-Rv2031c-P2, Th1-Rv2626c-P1, Th1-Rv2626c-P2, Th1-Rv2659c-P1, Th1-Rv2659c-P2, Th1-Rv2660c-P1, Th1-Rv2660c-P2, CTL-Rv1737c-P1, CTL-Rv1737c-P2, CTL-Rv2031c-P1, CTL-Rv2031c-P2, CTL-Rv2626c-P1, CTL-Rv2626c-P2, CTL-Rv2659c-P1, CTL-Rv2659c-P2, CTL-Rv2660c-P1, and CTL-Rv2660c-P2.

**TABLE 1:** Th1 and CTL immunodominant peptides selected to diagnose ATB and LTBI

Selected peptide	Allele	Position		Peptide sequence	Method	Percentile rank^*a*^
		Start	End			
Th1-Rv1737c-P3	HLA-DQA1*06:01/DQB1*03:03, HLA-DQA1*03:01/DQB1*06:01	162	176	TTHAIVAAALASTAV	NetMHCIIpan	0.06
Th1-Rv1737c-P5	HLA-DRB1*09:01	193	207	DPVLPRLKAAARLPV	Consensus (comb.lib./smm/nn)	0.08
Th1-Rv2031c-P1	HLA-DRB1*09:01	95	109	YGSFVRTVSLPVGAD	Consensus (comb.lib./smm/nn)	0.02
Th1-Rv2031c-P2	HLA-DRB1*07:01, HLA-DRB1*15:02, HLA-DRB5*01:01	92	106	EFAYGSFVRTVSLPV	Consensus (comb.lib./smm/nn)	0.04
Th1-Rv2626c-P1	HLA-DRB3*01:01	42	56	DDRLHGMLTDRDIVI	Consensus (comb.lib./smm/nn)	0.01
Th1-Rv2626c-P2	HLA-DRB1*09:01, HLA-DRB3*02:02, HLA-DRB1*15:04, HLA-DRB1*14:04, HLA-DRB1*14:01	128	142	IVQFVKAICSPMALA	Consensus (comb.lib./smm/nn)	0.04
Th1-Rv2659c-P1	HLA-DRB1*16:02, HLA-DRB1*15:04	294	308	PSALYRMFYKARKAA	NetMHCIIpan	0.01
Th1-Rv2659c-P2	HLA-DRB5*01:01, HLA-DRB1*14:04, HLA-DRB1*14:01, HLA-DRB1*12:02, HLA-DRB3*02:02	296	310	ALYRMFYKARKAAGR	Consensus (smm/nn/sturniolo)	0.02
Th1-Rv2660c-P1	HLA-DQA1*03:01/DQB1*06:01, HLA-DQA1*06:01/DQB1*03:03	2	16	IAGVDQALAATGQAS	NetMHCIIpan	1.79
Th1-Rv2660c-P2	HLA-DQA1*05:01/DQB1*03:01	16	30	SQRAAGASGGVTVGV	Consensus (comb.lib./smm/nn)	1.86
CTL-Rv1737c-P1	HLA-C*01:02	268	276	RIAPRHVVL	NetMHCpan	0.01
CTL-Rv1737c-P2	HLA-A*33:03	378	386	CTYTALHAR	NetMHCpan	0.02
CTL-Rv2031c-P1	HLA-C*03:04	23	31	AAFPSFAGL	NetMHCpan	0.04
CTL-Rv2031c-P2	HLA-A*33:03	92	100	EFAYGSFVR	NetMHCpan	0.05
CTL-Rv2626c-P1	HLA-C*03:04, HLA-C*01:02	133	141	KAICSPMAL	NetMHCpan	0.02
CTL-Rv2626c-P2	HLA-B*15:11	131	139	FVKAICSPM	NetMHCpan	0.03
CTL-Rv2659c-P1	HLA-B*15:11	202	210	MAAWLAMRY	NetMHCpan	0.02
CTL-Rv2659c-P2	HLA-C*03:04, HLA-C*01:02	325	333	LAASTGATL	NetMHCpan	0.02
CTL-Rv2660c-P1	HLA-B*15:11, HLA-B*15:02	47	56	FTFSSRSPDF	NetMHCpan	0.07
CTL-Rv2660c-P2	HLA-C*01:02, HLA-C*03:04, HLA-C*07:02, HLA-A*24:02	41	49	VVAPSQFTF	NetMHCpan	0.17

a A low percentile rank indicates high affinity. Median percentile ranks of the three methods were then used to generate the rank for the consensus method.

### ATB and LTBI mouse models were successfully constructed.

**FIG 1:**
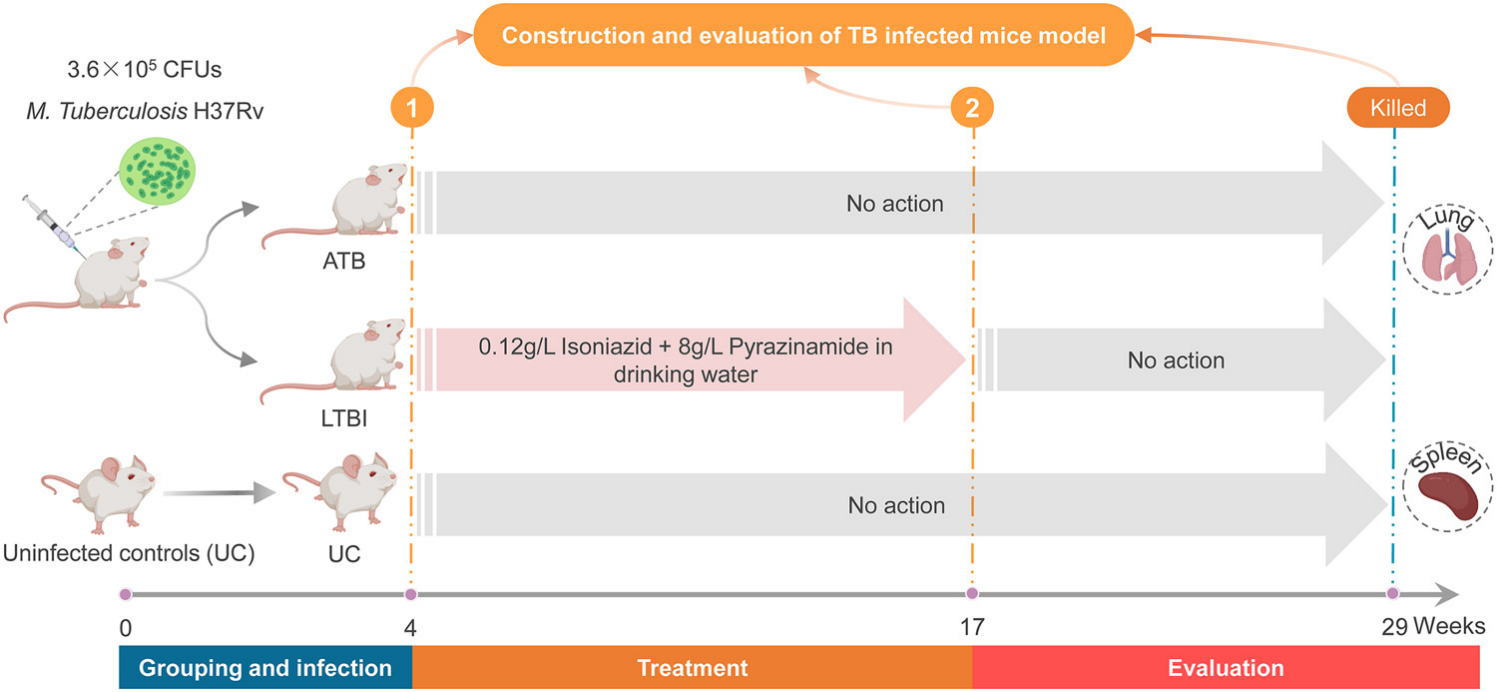
Flow chart of ATB and LTBI animal model construction.

**FIG 2:**
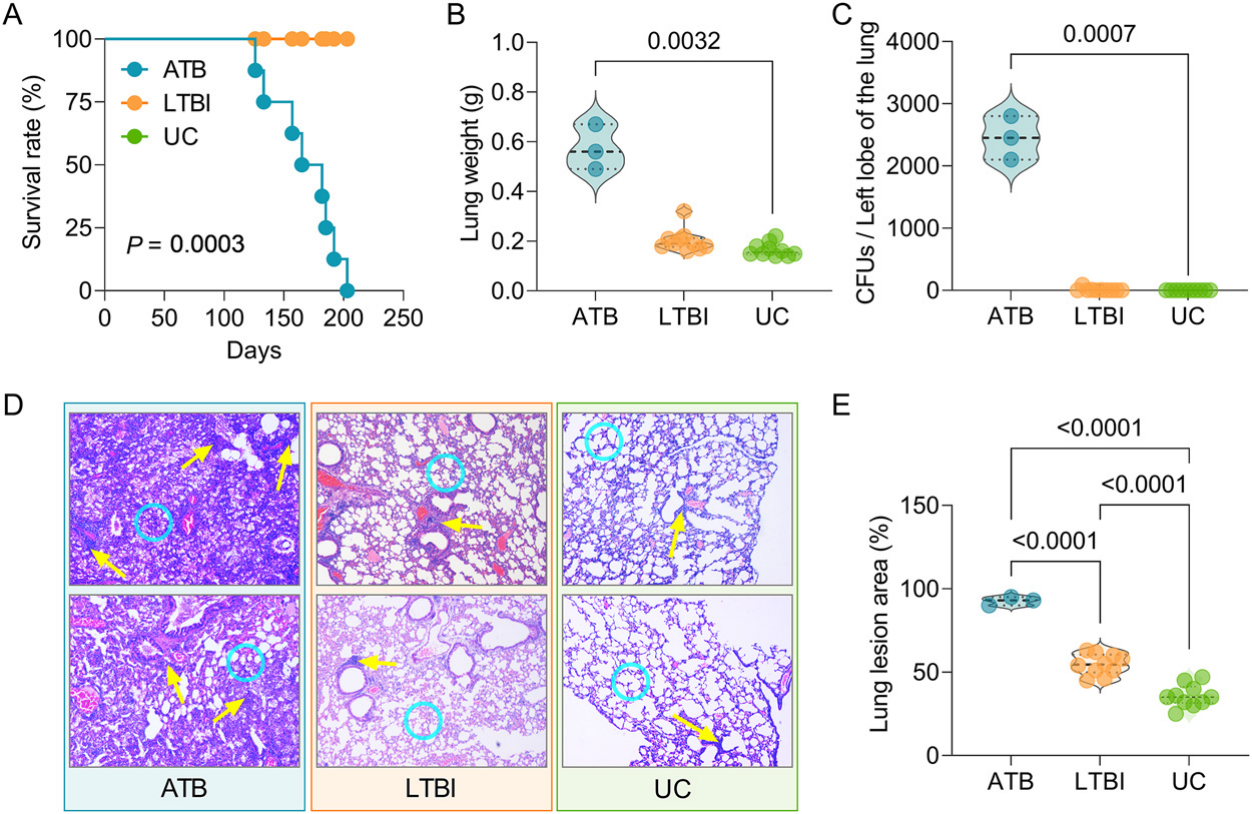
Evaluation of ATB and LTBI mouse models. Mice in ATB and LTBI groups were infected with M. tuberculosis. After 3 weeks, the mice in the LTBI group were treated with isoniazid and pyrazinamide for 19 weeks. (A) Survival rates of mice in each group were observed and recorded. At week 29, all mice in three groups were killed, and the lung weight (B) and CFU of left lobe of the lung (C) were analyzed. (D) Then, the right lobe of the lung was used to perform hematoxylin and eosin staining and observed using a microscope at an original ×40 magnification. Infiltration of inflammatory cells (shown with yellow arrows) and thickened alveolar walls (shown with blue circles) were observed in the lungs of ATB mice, but these lesions were relatively mild in LTBI mice and in uninfected control mice. (E) The lesion area of each mouse was observed and measured using software. The data are expressed as means ± the SEM and compared using one-way ANOVA or the Kruskal-Wallis test according to the data normality and homogeneity of variances. *P < *0.05 was considered significantly different.

To confirm the potential role of the dominant epitopes predicted from LTBI-RD antigens in diagnosing ATB and LTBI, we established a mouse model of ATB and a mouse model of LTBI (Fig. 1). Our results showed that eight mice in the ATB group died after M. tuberculosis infection, but mice in the LTBI group or the uninfected control (UC) group survived. The survival rate of the ATB group was significantly lower than that of the LTBI and UC groups (*P = *0.0003, Fig. 2A). The lung weights (*P = *0.0032, Fig. 2B) and the CFU loads (*P = *0.0007, Fig. 2C) of mice in the ATB group were significantly higher than those in the UC group. Although the lung weights and CFU loads of mice in the LTBI group were not statistically different from those in the ATB and UC groups, we found that the mean lung weights and CFU loads of mice in the LTBI group were higher than those in the UC group but much lower than those in the ATB group (Fig. 2B and C). Furthermore, lung lesions of mice in each group were observed under a ×40 microscope and counted using Image-Pro Plus software (Fig. 2D). The data showed that the lesion area of the right lobe of the lung collected from the ATB group was significantly larger than that in the LTBI group (*P < *0.0001) and UC group (*P < *0.0001), and the lesion area of the LTBI group was significantly larger than that in UC group (*P < *0.0001, Fig. 2E). These data indicated that the ATB and LTBI mouse models had been constructed successfully.

### Th1, CTL peptides, and their pools induced higher levels of IFN-γ^+^ T lymphocytes in ATB mice.

**FIG 3:**
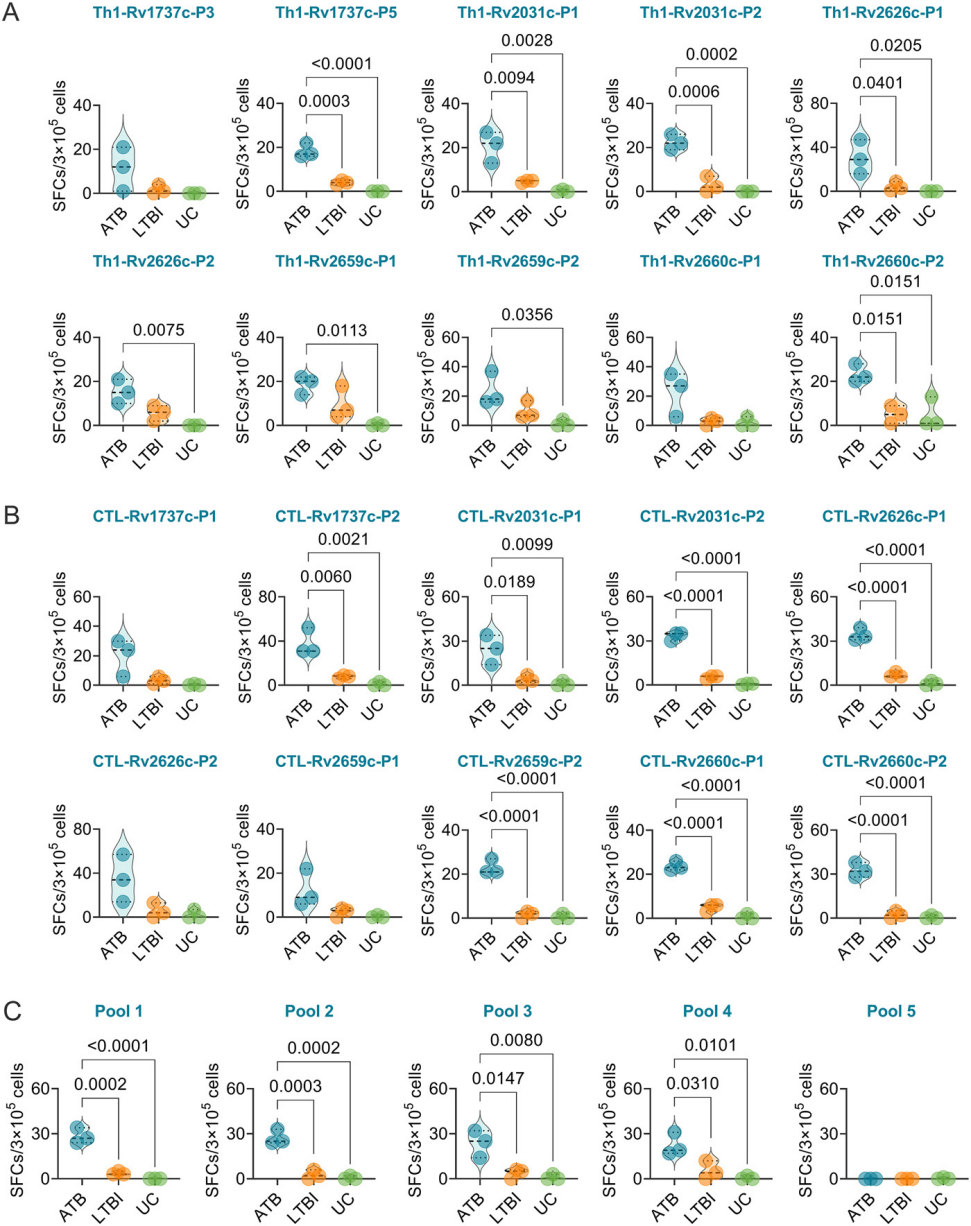
Detection of the number of IFN-γ^+^ T lymphocytes induced by dominant peptides in mice. The Th1 (A) and CTL (B) dominant peptides, as well as their pools (C), were used to stimulate the splenocytes collected from mice in the ATB, LTBI, or UC group *in vitro*. The number of IFN-γ^+^ T lymphocytes shown as SFCs per 3 × 10^5^ cells was determined by using a mouse ELISpot kit. The results were analyzed using the Kruskal-Wallis test or one-way ANOVA according to the normality and homogeneity of variances. Data are shown as means ± the SEM (*n *=* *3). *P < *0.05 was considered significantly different.

Splenocytes obtained from the spleens of mice in ATB, LTBI, and UC groups were stimulated with Th1 and CTL dominant peptides, as well as their pools (pool 1 to pool 5), respectively. The number of IFN-γ^+^ T lymphocytes were determined with spot-forming cells (SFCs; per 3 × 10^5^ cells). (i) Our results showed that for 10 Th1 dominant peptides (Fig. 3A), the numbers of IFN-γ^+^ T lymphocytes induced by Rv1737c-P5 (*P = *0.0003 or *P < *0.0001), Rv2031c-P1 (*P = *0.0094 or *P = *0.0028), Rv2031c-P2 (*P = *0.0006 or *P = *0.0002), Rv2626c-P1 (*P = *0.0401 or *P = *0.0205), and Rv2660c-P2 (*P = *0.0151 or *P = *0.0151) peptides were significantly higher in mice with ATB than in mice with LTBI or the UC group and that the numbers of IFN-γ^+^ T lymphocytes induced by Rv2626c-P2 (*P = *0.0075), Rv2659c-P1 (*P = *0.0113), and Rv2659c-P2 (*P = *0.0356) peptides were significantly higher in mice with ATB than in the UC group, but there was no significant difference in the number of IFN-γ^+^ T lymphocytes induced by peptides Rv1737c-P3 and Rv2660c-P1 among the three groups. (ii) For 10 CTL dominant peptides (Fig. 3B), the numbers of IFN-γ^+^ T lymphocytes induced by Rv1737c-P2 (*P = *0.0060 or *P = *0.0021), Rv2031c-P1 (*P = *0.0189 or *P = *0.0099), Rv2031c-P2 (*P < *0.0001 or *P < *0.0001), Rv2626c-P1 (*P < *0.0001 or *P < *0.0001), Rv2659c-P2 (*P < *0.0001 or *P < *0.0001), Rv2660c-P1 (*P < *0.0001 or *P < *0.0001), and Rv2660c-P2 (*P < *0.0001 or *P < *0.0001) peptides were significantly higher in mice with ATB than in mice with LTBI or the UC group, but there was no significant difference in the numbers of IFN-γ^+^ T lymphocytes induced by Rv1737c-P1, Rv2626c-P2, and Rv2659c-P1 among the three groups. (iii) For five peptide pools (Fig. 3C), the numbers of IFN-γ^+^ T lymphocytes induced by pool 1 (*P = *0.0002 or *P < *0.0001), pool 2 (*P = *0.0003 or *P = *0.0002), pool 3 (*P = *0.0147 or *P = *0.0080), and pool 4 (*P = *0.0310 or *P = *0.0101) were significantly higher in mice with ATB than that in mice with LTBI or the UC group, but there was no significant difference in the numbers of IFN-γ^+^ T lymphocytes induced by pool 5 among the three groups.

### The frequency of IFN-γ^+^ T lymphocytes induced by the combination of peptides could discriminate mice with LTBI from ATB and UC mice.

**FIG 4:**
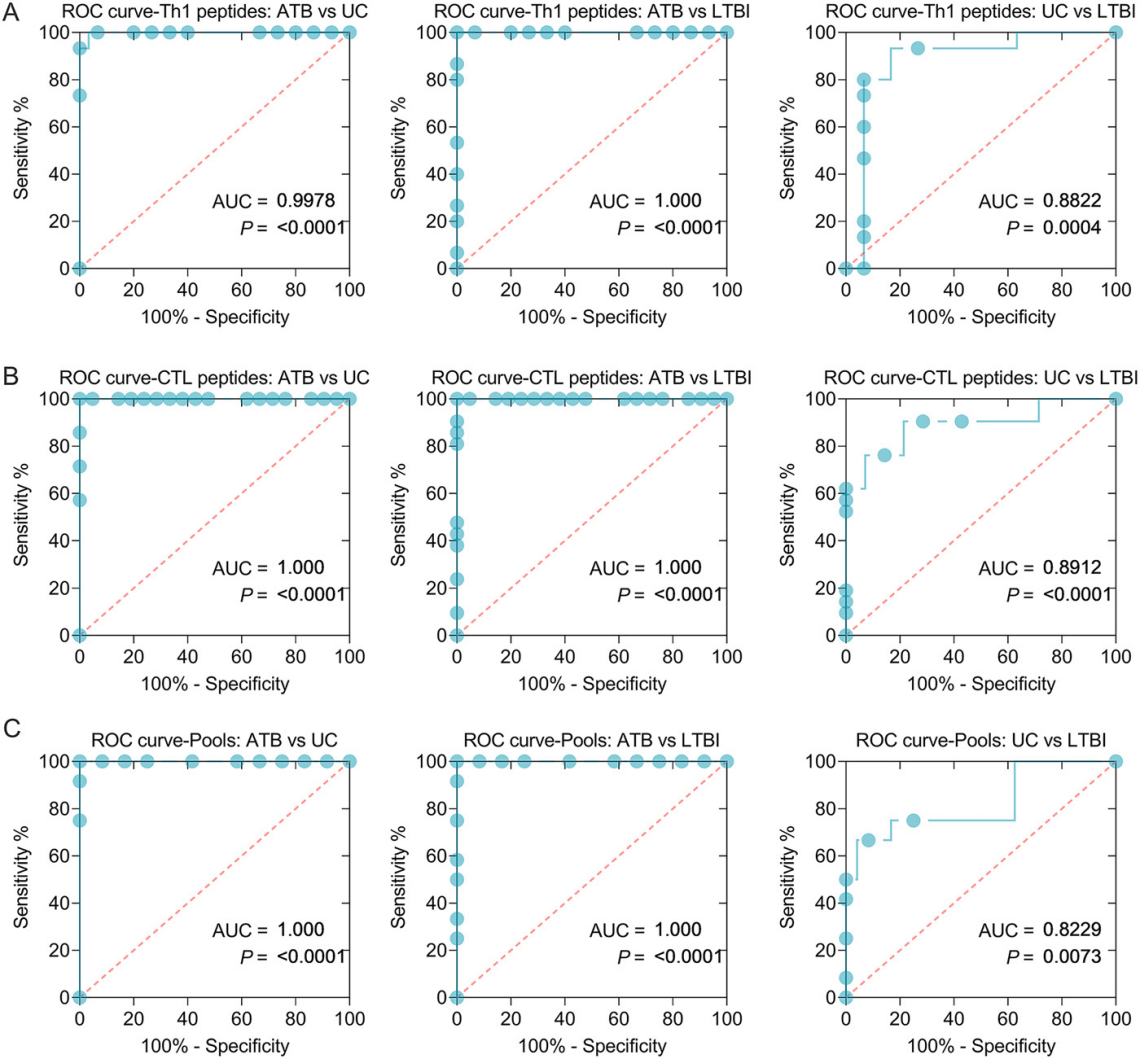
ROC curves of peptide-induced IFN-γ^+^ T lymphocytes in ATB, LTBI, and UC mice. ROC curves were used to determine the sensitivity and specificity of IFN-γ^+^ T lymphocytes induced by Th1 (A) and CTL (B) peptides and their pools (C) (see Table 2) in the diagnosis of ATB, LTBI, and UC by using the Wilson/Brown test. The AUC value and *P* value are shown in each chart. *P < *0.05 was considered significantly different.

Based on these results, we found that the frequency of IFN-γ^+^ T lymphocytes induced by five Th1 peptides (Th1-Rv1737c-P5, Th1-Rv2031c-P1, Th1-Rv2031c-P2, Th1-Rv2626c-P1, and Th1-Rv2660c-P2), seven CTL peptides (CTL-Rv1737c-P2, CTL-Rv2031c-P1, CTL-Rv2031c-P2, CTL-Rv2626c-P1, CTL-Rv2659c-P2, CTL-Rv2660c-P1, and CTL-Rv2660c-P2), and four peptide pools (1 to 4) was significantly higher in mice with ATB than in LTBI and UC mice. Therefore, these immunodominant peptides and their pools were analyzed by using an ROC curve. In this analysis, we combined five Th1 peptides or seven CTL peptides or four peptide pools together to improve their ability to differentiate mice with LTBI from ATB and UC mice (Fig. 4 and Table 2). In brief, (i) the frequency of IFN-γ^+^ T lymphocytes induced by combination of five Th1 peptides can distinguish mice with ATB from UC mice (Fig. 4A, area under the curve [AUC] = 0.9978, *P < *0.0001) or LTBI (AUC = 1, *P < *0.0001) and mice with LTBI from UC mice (AUC = 0.8822, *P = *0.0004); the sensitivities of ATB versus UC, ATB versus LTBI, and UC versus LTBI were 100, 100, and 80%, and the specificities were 93.33, 93.33, and 93.33%, respectively (Table 2). (ii) The frequency of IFN-γ^+^ T lymphocytes induced by combination of seven CTL peptides can distinguish mice with ATB from UC mice (Fig. 4B, AUC = 1, *P < *0.0001) or LTBI mice (AUC = 1, *P < *0.0001) and LTBI mice from UC mice (AUC = 0.8912, *P < *0.0001); the sensitivities of ATB versus UC, ATB versus LTBI, and UC versus LTBI were 100, 100, and 76.19%, and the specificities were 95.24, 95.24, and 85.71%, respectively (Table 2). (iii) The frequency of IFN-γ^+^ T lymphocytes induced by combination of four peptide pools can distinguish mice with ATB from UC mice (Fig. 4C, AUC = 1, *P < *0.0001) or LTBI mice (AUC = 1, *P < *0.0001), and mice with LTBI from UC mice (AUC = 0.8229, *P = *0.0073); the sensitivities of ATB versus UC, ATB versus LTBI, and UC versus LTBI were 100, 100, and 66.67%, and the specificities were 91.67, 91.67, and 91.67%, respectively (Table 2).

**TABLE 2:** Sensitivity and specificity of IFN-γ^+^ T lymphocytes induced by the combination of Th1 and CTL peptides in the diagnosis of ATB and LTBI

Peptide type	Merged peptides	Groups	*P*	AUC	95% CI	Cutoff value (SFCs/3 × 10^5^ cells)	%	
							Sensitivity	Specificity
Th1	Th1-Rv1737c-P5, Th1-Rv2031c-P1, Th1-Rv2031c-P2, Th1-Rv2626c-P1, and Th1-Rv2660c-P2	ATB vs UC	<0.0001	0.9978	0.9887–1.000	<14.50	100	93.33
		ATB vs LTBI	<0.0001	1	1.000–1.000	<14.50	100	93.33
		UC vs LTBI	0.0004	0.8822	0.7366–1.000	>1.50	80	93.33
CTL	CTL-Rv1737c-P2, CTL-Rv2031c-P1, CTL-Rv2031c-P2, CTL-Rv2626c-P1, CTL-Rv2659c-P2, CTL-Rv2660c-P1, and CTL-Rv2660c-P2	ATB vs UC	<0.0001	1	1.000–1.000	<17.50	100	95.24
		ATB vs LTBI	<0.0001	1	1.000–1.000	<17.50	100	95.24
		UC vs LTBI	<0.0001	0.8912	0.7878–0.9945	>2.50	76.19	85.71
Pools	Pool 1, pool 2, pool 3, and pool 4	ATB vs UC	<0.0001	1	1.000–1.000	<15.50	100	91.67
		ATB vs LTBI	<0.0001	1	1.000–1.000	<15.50	100	91.67
		UC vs LTBI	0.0073	0.8229	0.6454–1.000	>2.50	66.67	91.67

### Cytokines induced by six Th1 dominant peptides had the potential ability to discriminate mice with LTBI from ATB and UC mice.

**FIG 5:**
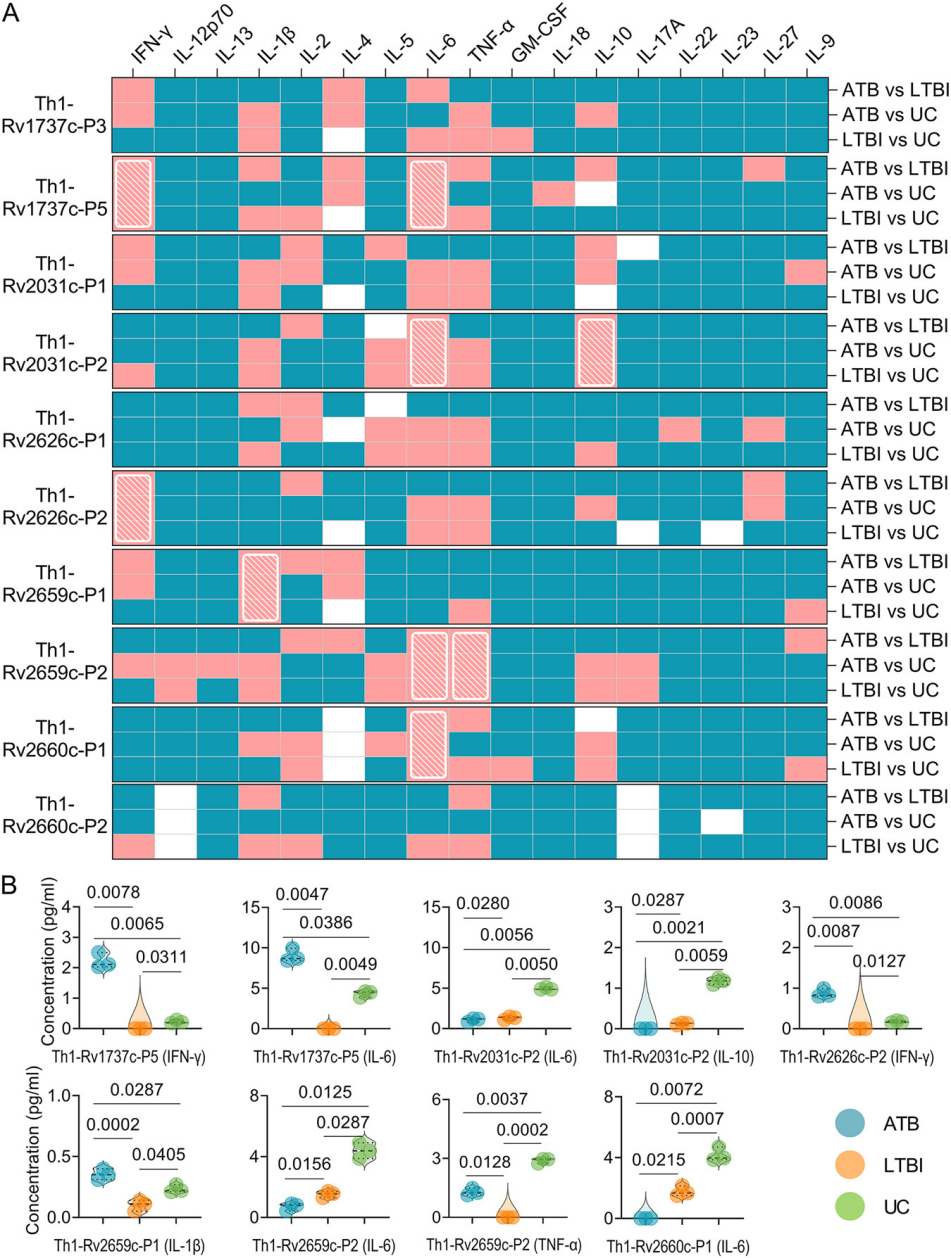
Cytokines induced by Th1 dominant peptides in mice. The splenocytes collected from mice in ATB, LTBI, and UC groups were stimulated with 10 Th1 dominant peptides for 48 h. The levels of IFN-γ, IL-12p70, IL-13, IL-1β, IL-2, IL-4, IL-5, IL-6, TNF-α, GM-CSF, IL-18, IL-10, L-17A, IL-22, IL-23, IL-27, and IL-9 cytokines in the supernatant were detected with a mouse Th1/Th2/Th9/Th17/Th22/Treg cytokine kit. The cytokine concentration differences among the three groups were compared using two-way ANOVA corrected with the Tukey test. All data were shown as means ± the SEM (*n *=* *3). (A) *P* value differences in the cytokine concentrations between groups (ATB versus LTBI, ATB versus UC, and LTBI versus UC) are shown as a heat map. *P < *0.05 was considered significantly different and is shown as a pink box, and *P ≥ *0.05 is shown as a blue box in the heat map. Furthermore, *P* values of <0.05 for cytokines induced by a peptide between the ATB versus LTBI, ATB versus UC, and LTBI versus UC groups are presented as a white diagonal grid in a heat map. (B) Detailed information is shown in violin plots.

To further clarify the potential value of 10 Th1 dominant peptides in differentiating LTBI, ATB, and UC mice, we stimulated spleen cells collected from LTBI, ATB, and UC mice *in vitro* with 10 Th1 dominant peptides. We detected the expression levels of 17 cytokines in culture supernatants. The levels of 17 cytokines induced by each Th1 dominant peptide were compared among three groups, and detailed information can be found in Fig. S1. To simplify and visualize many detailed data included in Fig. S1, we have produced a *P* value heat map (Fig. 5). We found that six Th1 dominant peptides (Th1-Rv1737c-P5, Th1-Rv2031c-P2, Th1-Rv2626c-P2, Th1-Rv2659c-P1, Th1-Rv2659c-P2, and Th1-Rv2660c-P1) can discriminate mice with LTBI from ATB and UC mice (Fig. 5A, shown with a white grid). In brief (Fig. 5B), (i) for Th1-Rv1737c-P5 peptide, the levels of IFN-γ (*P = *0.0078 or *P = *0.0065) and interleukin-6 (IL-6; *P = *0.0047 or *P = *0.0386) in mice with ATB were significantly higher than in LTBI or UC mice, and the levels of IFN-γ (*P = *0.0311) and IL-6 (*P = *0.0049) in mice with LTBI were significantly lower than in UC mice. (ii) For Th1-Rv2031c-P2 peptide, the levels of IL-6 (*P = *0.0280 or *P = *0.0056) and IL-10 (*P = *0.0287 or *P = *0.0021) in mice with ATB were significantly lower than in LTBI or UC mice, and the levels of IL-6 (*P = *0.0050) and IL-10 (*P = *0.0059) in mice with LTBI were significantly lower than in UC mice. (iii) For Th1-Rv2626c-P2 peptide, the level of IFN-γ (*P = *0.0087 or *P = *0.0086) in mice with ATB was significantly higher than in LTBI mice (*P = *0.0087) or UC mice (*P = *0.0086), and the level of IFN-γ in mice with LTBI was significantly lower than in UC mice (*P = *0.0127). (iv) For Th1-Rv2659c-P1 peptide, the level of IL-1β in mice with ATB was significantly higher than in mice with LTBI (*P = *0.0002) or in the UC group (*P = *0.0287), and the level of IFN-γ in mice with LTBI was significantly lower than in the UC group (*P = *0.0405). (v) For Th1-Rv2659c-P2 peptide, the levels of IL-6 (*P = *0.0156 or *P = *0.0125) and tumor necrosis factor alpha (TNF-α; *P = *0.0128 or *P = *0.0037) in mice with ATB were significantly lower or higher than in mice from the LTBI or UC group, and the levels of IL-6 (*P = *0.0287) and TNF-α (*P = *0.0002) in mice with LTBI were significantly lower than in the UC group. Lastly, (vi) for Th1-Rv2660c-P1 peptide, the level of IL-6 in mice with ATB was significantly lower than in mice with LTBI (*P = *0.0215) or in the UC group (*P = *0.0037), and the level of IL-6 in mice with LTBI was significantly lower than in the UC group (*P = *0.0007).

### Cytokines induced by four CTL dominant peptides had the potential ability to discriminate mice with LTBI from ATB and UC mice.

**FIG 6:**
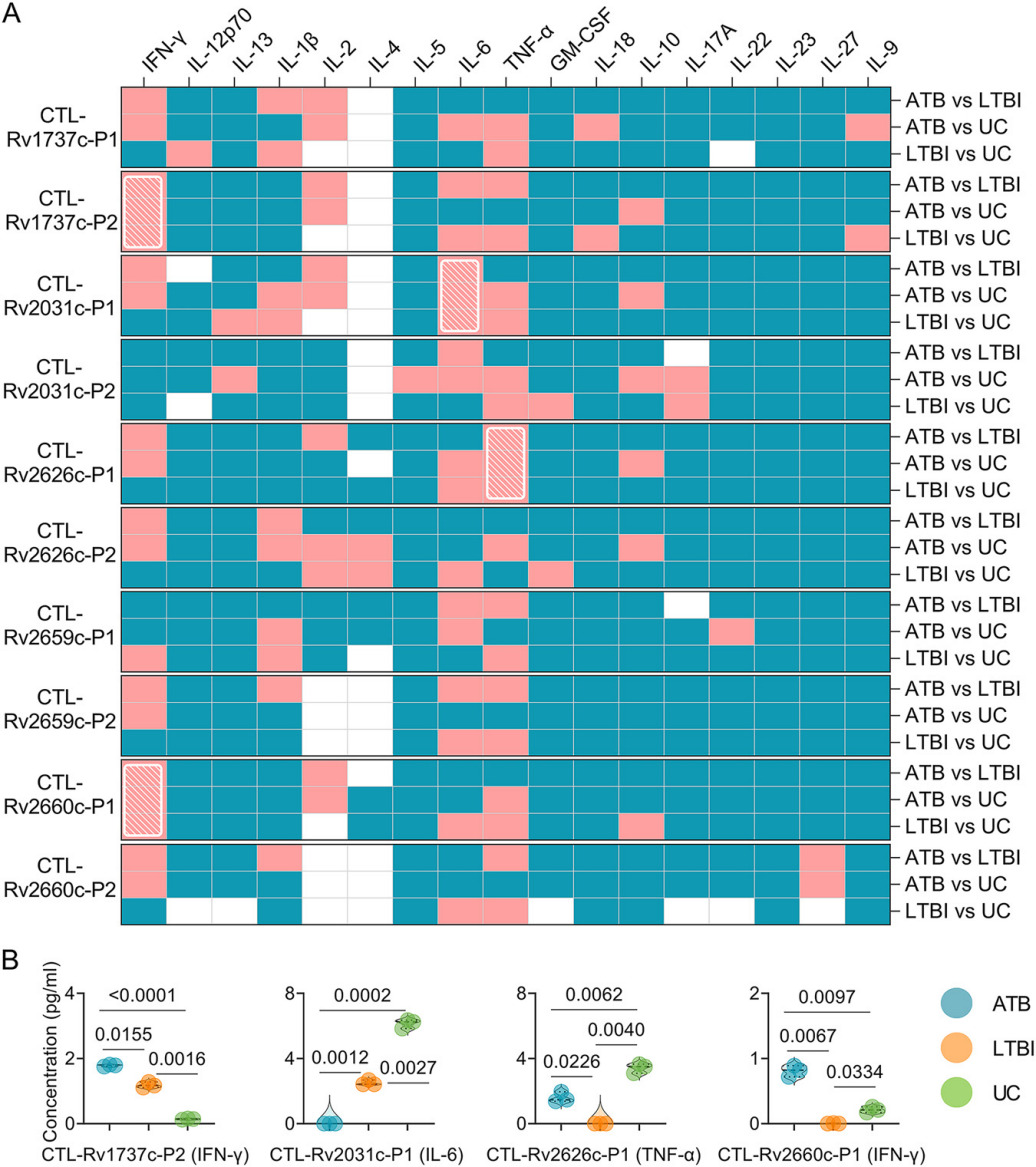
Cytokines induced by CTL dominant peptides in mice. The splenocytes collected from mice in ATB, LTBI, and UC groups were stimulated with the 10 CTL dominant peptides for 48 h. The levels of IFN-γ, IL-12p70, IL-13, IL-1β, IL-2, IL-4, IL-5, IL-6, TNF-α, GM-CSF, IL-18, IL-10, L-17A, IL-22, IL-23, IL-27, and IL-9 cytokines in the supernatant were detected with a mouse Th1/Th2/Th9/Th17/Th22/Treg cytokine kit. Cytokine concentration differences among the three groups were compared using two-way ANOVA corrected with the Tukey test. All data are shown as means ± the SEM (*n *=* *3). (A) *P* value differences in cytokine concentration between groups (ATB versus LTBI, ATB versus UC, and LTBI versus UC) are shown as a heat map. *P < *0.05 was considered significantly different and is indicated by a pink box, and *P ≥ *0.05 is indicated by a blue box in a heat map. Furthermore, the *P* values of <0.05 for cytokines induced by a peptide between the ATB versus LTBI, ATB versus UC, and LTBI versus UC groups are presented as a white diagonal grid in a heat map. (B) Detailed information is shown in violin plots.

Ten CTL dominant peptides were incubated with splenocytes from mice in three groups. The levels of 17 cytokines induced by them were detected to determine their ability to differentiate LTBI, ATB, and UC mice. Detailed information can be found in Fig. S2 in the supplemental material. We found that four CTL dominant peptides (CTL-Rv1737c-P2, CTL-Rv2031c-P1, CTL-Rv2626c-P1, and CTL-Rv2660c-P1) can discriminate mice with LTBI from ATB and UC mice (Fig. 6A, shown with a white grid). In brief (Fig. 6B), (i) for CTL-Rv1737c-P2 peptide, the level of IFN-γ in mice with ATB was significantly higher than in mice with LTBI (*P = *0.0155) or in the UC group (*P < *0.0001), and the levels of IFN-γ in mice with LTBI were significantly higher than in the UC group (*P = *0.0016). (ii) For CTL-Rv2031c-P1, the level of IL-6 in mice with ATB was significantly lower than in mice with LTBI (*P = *0.0012) or in the UC group (*P = *0.0002), and the levels of IL-6 in mice with LTBI were significantly lower than in the UC group (*P = *0.0027). (iii) For CTL-Rv2626c-P1, the level of TNF-α in mice with LTBI was significantly lower than in mice with ATB (*P = *0.0226) or in the UC group (*P = *0.0040), and the levels of TNF-α in mice with ATB were significantly lower than in UC group (*P = *0.0062). (iv) Finally, for CTL-Rv2660c-P1, the level of IFN-γ in mice with LTBI was significantly lower than in mice with ATB (*P = *0.0067) or in the UC group (*P = *0.0334), and the levels of IFN-γ in mice with ATB were significantly higher than in the UC group (*P = *0.0097).

### Cytokines induced by three peptide pools had the potential ability to discriminate mice with LTBI from ATB and UC mice.

**FIG 7:**
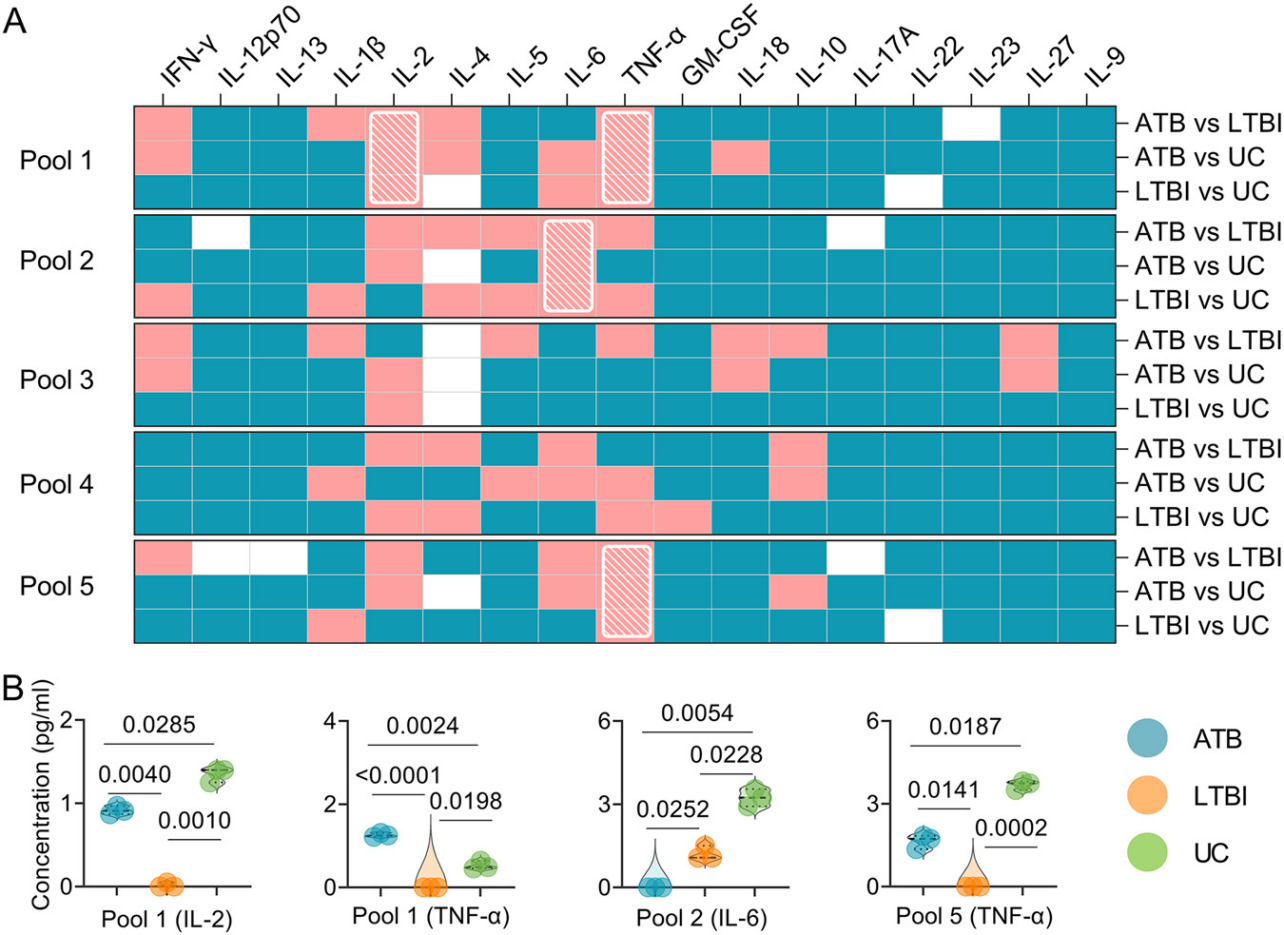
Cytokines induced by peptide pools in mice. The splenocytes collected from mice in ATB, LTBI, and UC groups were stimulated with the five peptides pools for 48 h. The levels of IFN-γ, IL-12p70, IL-13, IL-1β, IL-2, IL-4, IL-5, IL-6, TNF-α, GM-CSF, IL-18, IL-10, L-17A, IL-22, IL-23, IL-27, and IL-9 cytokines in the supernatant were detected with a mouse Th1/Th2/Th9/Th17/Th22/Treg cytokine kit. Cytokine concentration differences among the three groups were compared using two-way ANOVA corrected with a Tukey test. All data are shown as means ± the SEM (*n *=* *3). (A) *P* value differences in cytokine concentrations between groups (ATB versus LTBI, ATB versus UC, and LTBI versus UC) are shown as a heat map. *P < *0.05 was considered significantly different and is indicated by a pink box, and *P ≥ *0.05 is indicated by a blue box in a heat map. Furthermore, *P* values of <0.05 for cytokines induced by a peptide between the ATB versus LTBI, ATB versus UC, and LTBI versus UC groups are presented as a white diagonal grid in a heat map. (B) Detailed information is shown in violin plots.

Detailed information on cytokine levels generated by five pools can be found in Fig. S3. The results showed that three peptide pools (pools 1, 2, and 5) could discriminate mice with LTBI from ATB and UC mice (Fig. 7A, shown with a white grid). In brief (Fig. 7B), (i) for pool 1, the levels of IL-2 (*P = *0.0040 or *P = *0.0010) and TNF-α (*P < *0.0001 or *P = *0.0198) in mice with LTBI were significantly lower than in mice with LTBI or the UC group, the level of IL-2 in mice with ATB was significantly lower than in the UC group (*P = *0.0285), and the level of TNF-α in mice with ATB was significantly higher than in the UC group (*P = *0.0024). (ii) For pool 2, the level of IL-6 in mice with ATB was significantly lower than in mice with LTBI (*P = *0.0252) or in the UC group (*P = *0.0054), and the level of IL-6 in mice with LTBI was significantly lower than in the UC group (*P = *0.0228). (iii) For pool 5, the level of TNF-α in mice with LTBI was significantly lower than in mice with ATB (*P = *0.01416) or in the UC group (*P = *0.0002), and the level of TNF-α in mice with ATB was significantly lower than in the UC group (*P = *0.0187).

### The sensitivity and specificity of IFN-γ, IL-6, and TNF-α cytokines in discriminating ATB, LTBI, and UC mice.

**FIG 8:**
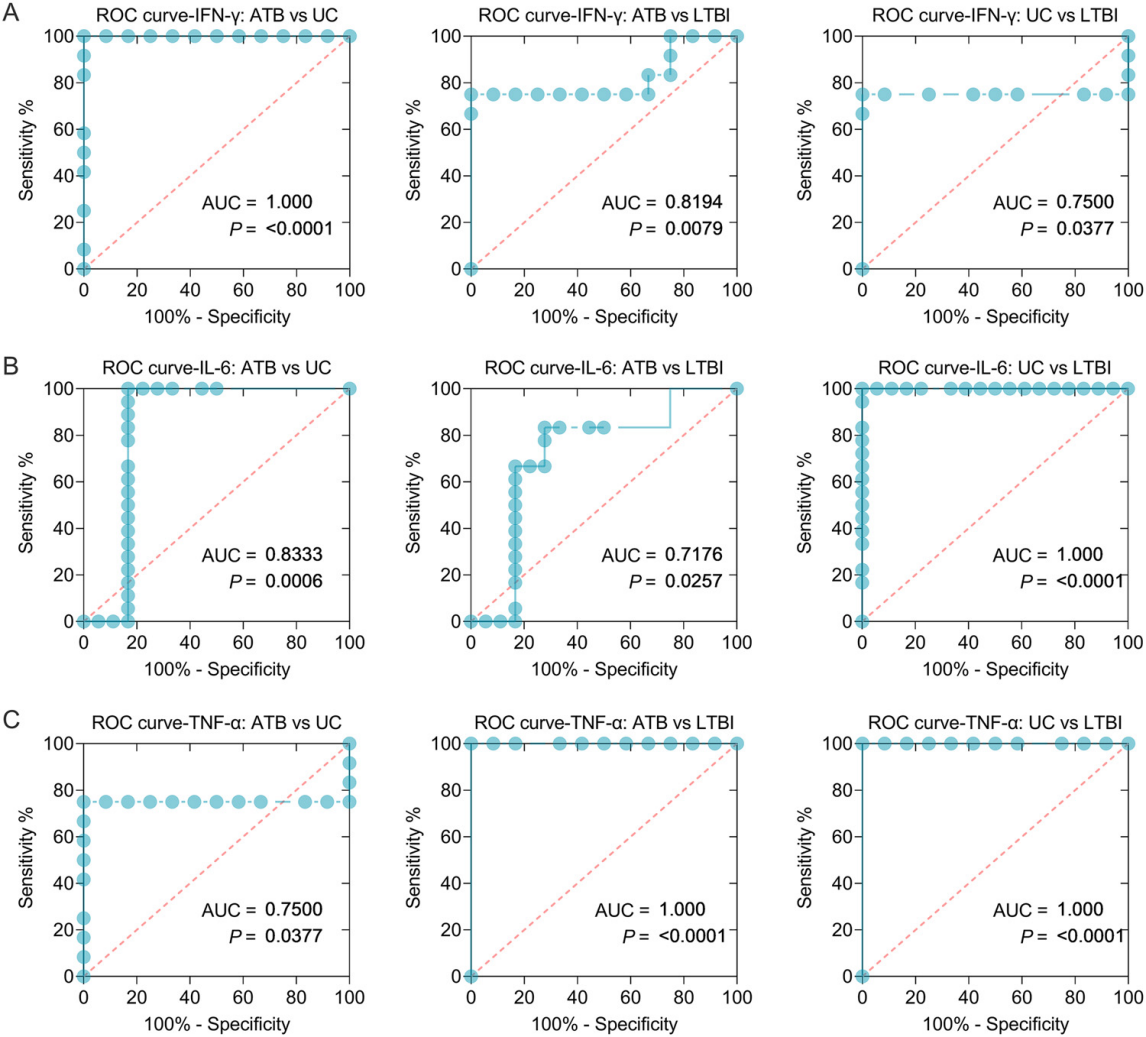
ROC curves of peptide-induced cytokines in ATB, LTBI, and UC mice. ROC curves were used to determine the sensitivity and specificity of IFN-γ (A), IL-6 (B), and TNF-α (C) cytokines induced by merged peptides (Table 3) in the diagnosis of ATB, LTBI, and UC using the Wilson/Brown test. The AUC value and *P* value are shown in each chart. *P < *0.05 was considered significantly different.

Based on these experimental results, three cytokines (IFN-γ, IL-6, and TNF-α) with significant differences among ATB, LTBI, and UC groups were selected for further analysis, and the sensitivity and specificity of each cytokine in the differential diagnosis of ATB, LTBI, and UC were evaluated by using an ROC curve (Table 3). The combined peptides or peptide pools induced significantly different cytokines between the three groups, improving their ability to differentiate ATB from LTBI (Fig. 8 and Table 3). In brief, (i) the level of IFN-γ induced by Th1-Rv1737c-P5, Th1-Rv2626c-P2, CTL-Rv1737c-P2, and CTL-Rv2660c-P1 peptides could distinguish ATB mice from UC (Fig. 8A, AUC = 1, *P < *0.0001) or LTBI (AUC = 0.8194, *P = *0.0079) mice and LTBI mice from UC mice (AUC = 0.7500, *P = *0.0377), the sensitivities of ATB versus UC, ATB versus LTBI, and UC versus LTBI were 100, 75, and 75%, and the specificities were 91.67, 91.67, and 91.67%, respectively (Table 3). (ii) The level of IL-6 induced by Th1-Rv1737c-P5, Th1-Rv2031c-P2, Th1-Rv2659c-P2, Th1-Rv2660c-P1, and CTL-Rv2031c-P1 peptides, as well as pool 2, could distinguish ATB from UC (Fig. 8B, AUC = 0.8333, *P = *0.0006) or LTBI (AUC = 0.7176, *P = *0.0257) mice and LTBI from UC mice (AUC = 1, *P < *0.0001); the sensitivities of ATB versus UC, ATB versus LTBI, and UC versus LTBI were 100, 66.67, and 100%, and the specificities were 83.33, 83.33, and 94.44%, respectively (Table 3). (iii) The level of TNF-α induced by Th1-Rv2659c-P2, CTL-Rv2626c-P1, pool 1, and pool 5 could distinguish ATB from UC mice (Fig. 8C, AUC = 0.7500, *P = *0.0377) or LTBI mice (AUC = 1, *P < *0.0001), and LTBI from UC mice (AUC = 1, *P < *0.0001); the sensitivities of ATB versus UC, ATB versus LTBI, and UC versus LTBI were 75, 100, and 100%, and the specificities were 91.67, 91.67, and 91.67%, respectively (Table 3).

**TABLE 3:** Sensitivity and specificity of IFN-γ, TNF-α, and IL-6 cytokines induced by the combination of Th1 and CTL peptides in the diagnosis of ATB and LTBI

Cytokine	Merged peptides	Groups	*P*	AUC	95% CI	Cutoff value (pg/mL)	%	
							Sensitivity	Specificity
IFN-γ	Th1-Rv1737c-P5, Th1-Rv2626c-P2, CTL-Rv1737c-P2, CTL-Rv2660c-P1	ATB vs UC	<0.0001	1.0000	1.0000–1.0000	<0.3850	100	91.67
		ATB vs LTBI	0.0079	0.8194	0.6294–1.0000	<0.3850	75	91.67
		UC vs LTBI	0.0377	0.7500	0.5050–0.9950	<0.0900	75	91.67
IL-6	Th1-Rv1737c-P5, Th1-Rv2031c-P2, Th1-Rv2659c-P2, Th1-Rv2660c-P1, CTL-Rv2031c-P1, pool 2	ATB vs UC	0.0006	0.8333	0.6612–1.0000	>2.0750	100	83.33
		ATB vs LTBI	0.0257	0.717	0.5320–0.9032	>1.2550	66.67	83.33
		UC vs LTBI	<0.0001	1.0000	1.0000–1.0000	<3.0850	100	94.44
TNF-α	Th1-Rv2659c-P2, CTL-Rv2626c-P1, pool 1, pool 5	ATB vs UC	0.0377	0.7500	0.5050–0.9950	>1.900	75%	91.67
		ATB vs LTBI	<0.0001	1.0000	1.0000–1.0000	<1.175	100	91.67
		UC vs LTBI	<0.0001	1.0000	1.0000–1.0000	<0.4750	100	91.67

### Recombinant CFP10-ESAT6 protein (CE protein) and pool 2 discriminated patients with ATB or LTBI from the UC group.

**FIG 9:**
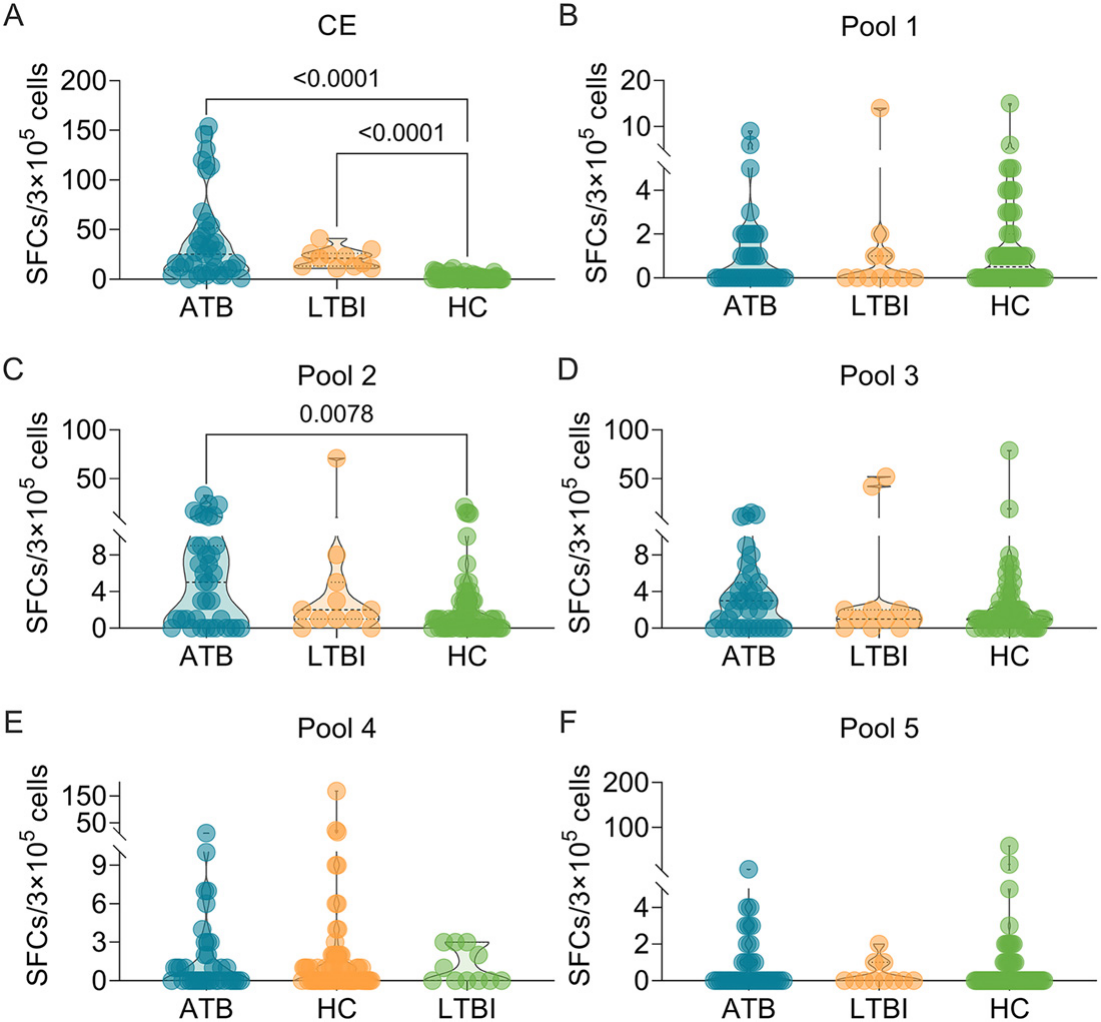
Detection of the number of IFN-γ^+^ T lymphocytes induced by peptide pools in humans. The CE protein (A), pool 1 (B), pool 2 (C), pool 3 (D), pool 4 (E), and pool 5 (F) were used to stimulate PBMCs collected from patients with ATB (*n *=* *37) or LTBI (*n *=* *11) or in the UC group (*n *=* *62). The number of IFN-γ^+^ T lymphocytes (shown as SFCs per 3 × 10^5^ cells) was determined by using a human ELISpot assay. The results were analyzed using the Kruskal-Wallis test or one-way ANOVA according to the normality and homogeneity of variances. Data are shown as means ± the SEM. *P < *0.05 was considered significantly different.

**FIG 10:**
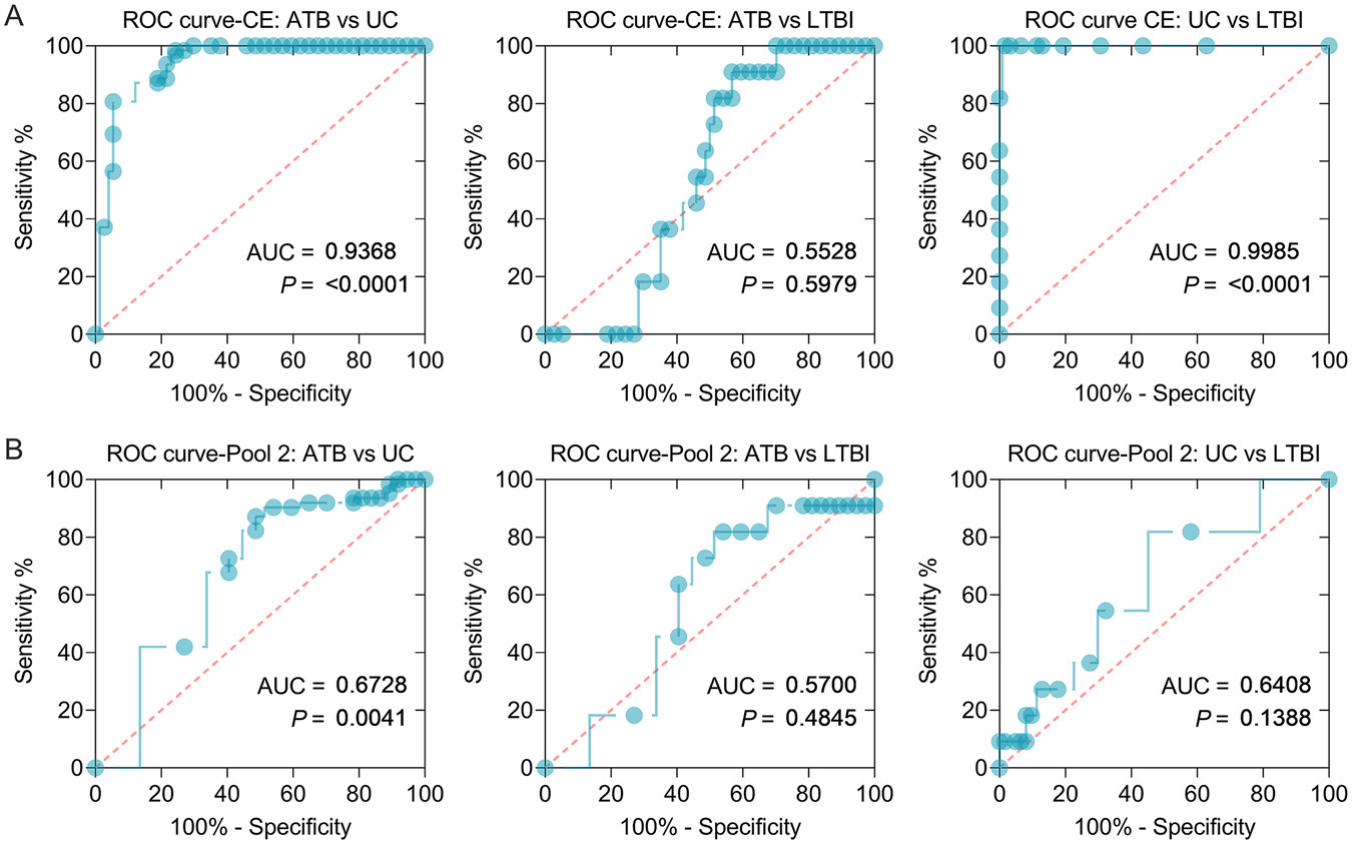
ROC curves of IFN-γ^+^ T lymphocytes induced by CE protein and pool 2 peptides in ATB, LTBI, and UC humans. ROC curves were used to determine the sensitivity and specificity of IFN-γ^+^ T lymphocytes induced by CE protein (A) and pool 2 peptides (B) in diagnosing ATB, LTBI, and UC by using the Wilson/Brown test. The AUC value and *P* value are shown in each chart. *P < *0.05 was considered significantly different.

To further corroborate the results obtained from the mouse model, we explored the potential roles of dominant peptides in human participants. Considering the specificity of human samples and the affordability of experimental costs, we selected CE protein (Fig. 9A), pool 1 (Fig. 9B), pool 2 (Fig. 9C), pool 3 (Fig. 9D), pool 4 (Fig. 9E), and pool 5 (Fig. 9F) for human ELISpot experiments. The results showed that the number of IFN-γ^+^ T lymphocytes induced by CE protein was significantly higher in patients with ATB (*P < *0.0001) or LTBI (*P < *0.0001) than in UC participants, and the number of IFN-γ^+^ T lymphocytes induced by pool 2 peptides was significantly higher in patients with ATB than in UC participants. However, there was no significant difference in the number of IFN-γ^+^ T lymphocytes induced by pool 1, pool 3, pool 4, and pool 5 among the three groups. In addition, we also analyzed the sensitivity and specificity of CE protein and pool 2 in discriminating patients with ATB, LTBI individuals, and UC volunteers (Fig. 10). Our results indicated that the number of IFN-γ^+^ T lymphocytes induced by CE protein could distinguish patients with ATB from UC (Fig. 10A, AUC = 0.9368, *P < *0.0001) and LTBI individuals from UC (AUC = 0.9985, *P < *0.0001), but it could not distinguish patients with ATB from LTBI individuals (AUC = 0.5528, *P = *0.5979). The sensitivity and specificity of CE protein in ATB versus UC subjects were 80.65 and 94.59%, with a cutoff value of < 3.500, and the sensitivity and specificity of CE protein in UC versus LTBI subjects were 100 and 98.39%, with a cutoff value of  >10.000, respectively. Interestingly, we found that the number of IFN-γ^+^ T lymphocytes induced by pool 2 peptides could distinguish patients with ATB from the UC group (Fig. 10B, AUC = 0.6728, *P = *0.0041). The sensitivity and specificity of pool 2 in ATB versus UC were 72.58 and 59.46%, with a cutoff value of < 2.500.

## DISCUSSION

The biggest obstacle to TB prevention, diagnosis, and treatment is the lack of understanding of the pathogenesis of M. tuberculosis and its interaction with the host. Both the innate and the adaptive immunity of the host play a crucial role in eliminating or killing the mycobacteria, but the mechanism is still unclear (18). It is generally accepted that macrophages, dendritic cells (DCs), and natural killer (NK) cells are the first-line cells for fighting against M. tuberculosis. Macrophages and DCs, as the most important APCs, play an essential role in the phagocytosis of M. tuberculosis (19). DCs activated by M. tuberculosis migrate to lymph nodes, where they present and display mycobacterium peptides on their surfaces. These peptides will be recognized by CD4^+^ T cells and CD8^+^ T cells via the MHC-II and MHC-I molecules, respectively (7, 20). Interestingly, recognition between T cells and APCs is based on some essential peptides rather than the full-length protein. Therefore, the selection of candidate antigens and the prediction and screening of potential immunodominant peptides have become a key to designing a new generation of TB diagnostic biomarkers. A peptide-based diagnostic biomarker can not only be recognized and efficiently presented by more MHC molecules but also compensate for the immune failure caused by the mutation of a dominant epitope of the pathogen. Due to these unique advantages, peptides have gradually been paid more and more attention by scholars and have become one of the hot spots in developing vaccines and diagnostic biomarkers (21).

Our previous study suggests that antigens belonging to latency and RDs are the most novel and promising targets for LTBI differential diagnosis (7). Therefore, identifying biomarkers derived from LTBI-RD antigens is the key to solving the problem of LTBI and ATB diagnosis in China. Here, five candidate antigens related to LTBI-RD were selected. Previous studies have reported that the levels of IFN-γ production in human peripheral blood mononuclear cells (PBMCs) induced by all of them were significantly different between TB patients and LTBI, suggesting that these antigens have potential in the differential diagnosis of LTBI and ATB (22–39). M. tuberculosis is an intracellular parasite, and the host’s elimination and killing of invading mycobacteria mainly depend on antigen-specific T cells, including CD4^+^ T lymphocytes and CD8^+^ T lymphocytes (40). Thus, we predicted 70 Th1 and 49 CTL epitopes from five antigens and identified the top 10 Th1 and CTL dominant epitopes for further exploration.

To reduce the effect of human PBMC cellular heterogeneity on the efficacy of peptide diagnostics, we constructed ATB and LTBI animal models over 29 weeks. Our results indicated that the survival rate of mice in the ATB group was significantly lower than those of mice in the LTBI and UC groups. The lung weight and CFUs in the lungs (left lobe) of mice in the ATB group were remarkably higher than those of mice in the LTBI and UC groups, and the lung lesion areas of mice in the LTBI group were significantly smaller than those of mice in ATB group but larger than those of mice in the UC group. These data suggested that the ATB and LTBI animal models have been successfully constructed. Based on this animal tool, we further explored the number of IFN-γ^+^ T lymphocytes induced by candidate Th1 and CTL peptides and their pools among mice in the ATB, LTBI, and UC groups. We found that five Th1 dominant peptides (Rv1737c-P5, Rv2031c-P1, Rv2031c-P2, Rv2626c-P1, and Rv2660c-P2), seven CTL dominant peptides (Rv1737c-P2, Rv2031c-P1, Rv2031c-P2, Rv2626c-P1, Rv2659c-P2, Rv2660c-P1, and Rv2660c-P2), and four peptide pools (pool 1, pool 2, pool 3, and pool 4) elicited higher frequencies of IFN-γ^+^ T lymphocytes in ATB mice than in LTBI and UC mice. It has been reported that IFN-γ^+^ T lymphocytes play an essential role in defending against M. tuberculosis infection by producing a high level of IFN-γ (21, 41). The number of IFN-γ^+^ T lymphocytes and level of IFN-γ production have been used to diagnose ATB by ELISpot or enzyme-linked immunosorbent assay (7). Here, we explored the diagnostic value of the number of IFN-γ^+^ T lymphocytes elicited with the combination of Th1 immunodominant peptides, CTL immunodominant peptides, and the combination of their pools. The results indicated that these three combinations had excellent performance in the differential diagnosis of mice with LTBI and ATB, especially in the differential diagnosis of ATB versus UC mice and of ATB versus LTBI (both sensitivity and specificity reached 100%). These data suggest that the combination of five Th1 immunodominant peptides, seven CTL immunodominant peptides, or four peptide pools can differentially diagnose mice with LTBI from mice with ATB or UC mice.

Cytokines have been reported as promising biomarkers for distinguishing ATB cases from controls or LTBI individuals from patients with ATB or controls, including IFN-γ, TNF-α, IL-1ra, IL-1β, IL-2, IL-10, and granulocyte-macrophage colony-stimulating factor (GM-CSF) (42, 43). In the present study, we assessed the levels of 17 Th1/Th2/Th9/Th17/Th22/Treg cytokines among ATB, LTBI, and UC mouse groups. We found that the levels of IFN-γ, IL-1β, IL-2, IL-6, IL-10, and TNF-α induced by Th1 and CTL immunodominant peptides and their pools were significantly different among the three groups. A meta-analysis showed that the cytokines could assist the discrimination between ATB and LTBI, but a single biomarker for the differential diagnosis of ATB and LTBI is often ineffective due to poor diagnostic sensitivity and specificity (44). Recently, it has been proposed that multiple biomarkers combined to diagnose ATB and LTBI can significantly improve the sensitivity and specificity (7, 45, 46). Based on this theory, we combined different Th1 and CTL immunodominant peptides, as well as their pools, to improve the diagnostic efficacy. It was observed that (i) the combination of Th1-Rv1737c-P5, Th1-Rv2626c-P2, CTL-Rv1737c-P2, and CTL-Rv2660c-P1 peptides, (ii) the combination of Th1-Rv1737c-P5, Th1-Rv2031c-P2, Th1-Rv2659c-P2, Th1-Rv2660c-P1, CTL-Rv2031c-P1, and pool 2 peptides, and (iii) the combination of Th1-Rv2659c-P2, CTL-Rv2626c-P1, pool 1, and pool 5 peptides could significantly distinguish mice with LTBI from mice with ATB or the UC group with high sensitivity and specificity. These results indicated that the combinations between Th1 and CTL peptides and their pools could significantly enhance the performance of these three cytokines in mouse models.

All of the above data were obtained from animal models, which may differ from human beings. Therefore, to verify the performance of these immunodominant peptides in differentiating patients with ATB from LTBI individuals, we selected CE protein and five peptide pools (pool 1 to pool 5) for further exploration. The results showed that CE protein induced significantly higher levels of IFN-γ^+^ T lymphocytes in patients with ATB and LTBI than in people in the UC group, and pool 2 induced a significantly higher level of IFN-γ^+^ T lymphocytes in patients with ATB than in people in the UC group. In addition, we also found that CE could discriminate patients with ATB or LTBI from uninfected controls but could not discriminate LTBI individuals from patients with ATB, a finding consistent with previous studies (7). Interestingly, we observed that pool 2 could distinguish patients with ATB from uninfected controls. The sensitivity and specificity of pool 2 were 72.58 and 59.46%, with a cutoff value of <2.500.

This study has several limitations, which should be improved in the future. (i) Although as many as 70 Th1 and 49 CTL dominant epitopes were predicted by bioinformatics technology, only the top 10 Th1 and CTL dominant epitopes were synthesized *in vitro* due to financial constraints, which may increase the probability of losing potential epitopes in TB diagnosis. (ii) All of the Th1 and CTL epitopes were predicted to bind high-frequency MHC-II- and MHC-I-restricted alleles in the Chinese population, but the animal model used in this study was wild-type mice rather than MHC humanized mice. (iii) Finally, the sample size of LTBI individuals was only 11, which is smaller than that for patients with ATB and UC.

### Conclusions.

In summary, the results of ELISpot assay suggested that (i) the combination of Th1-Rv1737c-P5, Th1-Rv2031c-P1, Th1-Rv2031c-P2, Th1-Rv2626c-P1, and Th1-Rv2660c-P2 peptides, (ii) the combination of CTL-Rv1737c-P2, CTL-Rv2031c-P1, CTL-Rv2031c-P2, CTL-Rv2626c-P1, CTL-Rv2659c-P2, CTL-Rv2660c-P1, and CTL-Rv2660c-P2 peptides, and (iii) the combination of pool 1, pool 2, pool 3, and pool 4 demonstrated excellent performance in discriminating mice with ATB and LTBI based on the frequency of IFN-γ^+^ T lymphocytes. Furthermore, the results of Th1/Th2/Th9/Th17/Th22/Treg cytokine analysis indicated that (i) the combination of Th1-Rv1737c-P5, Th1-Rv2626c-P2, CTL-Rv1737c-P2, and CTL-Rv2660c-P1 peptides, (ii) the combination of Th1-Rv1737c-P5, Th1-Rv2031c-P2, Th1-Rv2659c-P2, Th1-Rv2660c-P1, CTL-Rv2031c-P1, and pool 2 peptides, and (iii) the combination of Th1-Rv2659c-P2, CTL-Rv2626c-P1, pool 1, and pool 5 peptides showed excellent performance in discriminating mice with ATB and LTBI based on the levels of IFN-γ, IL-6, and TNF-α, respectively. We also confirmed that pool 2 might distinguish patients with ATB from uninfected controls, but its specificity was 59.46%. To our knowledge, this study is the first to report that immunodominant Th1 and CTL peptides derived from LTBI-RD antigens have unique advantages in distinguishing LTBI from ATB in mice and ATB patients from uninfected individuals, which provides a new understanding of these peptides in the differential diagnosis of ATB and LTBI in humanized animal models and large sample populations for the future.

## MATERIALS AND METHODS

### Ethics statements.

The construction of LTBI and ATB mouse models was conducted according to the Experimental Animal Regulation Ordinances principles released by the China National Science and Technology Commission. The mice were raised in a specific-pathogen-free laboratory and operated under the approval of the Ethics Committee of the Eighth Medical Center of the PLA General Hospital (approval 309201904081530). Mice were challenged with the M. tuberculosis H37Rv strain in a qualified negative pressure biosafety laboratory level-2 PLUS (negative pressure BSL-2 PLUS) in the Eighth Medical Center of the PLA General Hospital. Furthermore, the human PBMCs were obtained from patients with ATB or LTBI individuals and from UCs according to the principles of the Declaration of Helsinki established by the World Medical Association (WMA), as well as the Ethical Review of Biomedical Research Involving Humans released by National Health and Family Planning Commission of China (NHFPC). All participants were informed of the details of the experiment and agreed to sign informed consent. The practical steps related to PBMC separation were approved by the Medical Ethics Committee of the Eighth Medical Center of PLA General Hospital (approval 309201904081530).

### Inclusion and exclusion criteria for participants.

The diagnostic criteria of patients with ATB included in this study were obtained following the “Diagnostic Criteria for Pulmonary Tuberculosis WS288-2017” issued by the National Health Commission of the People’s Republic of China. In brief, the diagnosis of TB is mainly based on the examination of etiology (including bacteriology and molecular biology), combined with a comprehensive analysis of epidemiological history, clinical manifestations, chest imaging, related auxiliary examinations, and differential diagnosis. Among them, etiology and pathology results are first considered. The exclusion criteria for patients with ATB include (i) those who use hormones; (ii) those who have the diseases that affect immune function such as HIV infection, posttransplantation, and autoimmune diseases; (iii) those who are malnourished; and (iv) children younger than 12 years old.

The inclusion criteria for participants with LTBI were as follows: (i) a history of close contact with ATB patients or health care workers (HCWs) in a TB specialized hospital or laboratory, (ii) IGRA positive, (iii) no TB clinical manifestations and normal chest X-ray, (iv) over 12 years of age, and (v) HIV negative. The exclusion criteria for participants with LTBI were as follows: (i) confirmed or suspected TB patients, (ii) pregnant women and lactating women, (iii) previously received anti-TB treatment for more than 1 month, (iv) children under 12 years old, and (v) HIV positive.

The inclusion criteria for UC group were as follows: (i) no history of contact with TB patients; (ii) IGRA negative; (iii) no TB clinical manifestations and normal chest X-ray, excluding a diagnosis of ATB; and (iv) HIV negative. The exclusion criteria for the UC group were as follows: (i) those who had a history of travel or residence in high-risk areas of TB, (ii) HCWs in a TB specialized hospital or laboratory, (iii) children under 12 years old, (iv) those who cannot be tested for IGRA or are allergic to CE protein, and (v) HIV positive.

### Selection of target antigen.

Based on our previous study, antigens belonging to both the RD and LTBI are the most novel and promising targets for LTBI differential diagnosis (7). Furthermore, previous studies found that the levels of IFN-γ stimulated by five antigens (Rv1737c, Rv2031c, Rv2626c, Rv2659c, and Rv2660c) in PBMCs were significantly different between TB and LTBI patients, suggesting that these antigens have a potential role in the differential diagnosis of ATB and LTBI (22–39). Therefore, the above-listed five antigens associated with LTBI-RD were selected as target candidates. Detailed information on these five antigens can be found in Table 4.

**TABLE 4:** Characteristics of the candidate antigens related to LTBI-RD^*a*^

Antigen	Gene	Protein ID	Product	Length (aa)	RD	LTBI	References
Rv1737c	*narK2*	CCP44503.1	Possible nitrate/nitrite transporter NarK2	395	RD-Others	DosR	22 – 24
Rv2031c	*HspX*	CCP44804.1	Heat shock protein HspX, HSP16.3	144	RD-Others	DosR	25 – 30
Rv2626c	*hrp1*	CCP45424.1	Hypoxic response protein 1 Hrp1	143	RD-Others	DosR	22, 25, 31–33
Rv2659c	*Rv2659c*	CCP45457.1	Possible PhiRv2 prophage protein	375	RD13	NS	34 – 38
Rv2660c	*Rv2660c*	CCP45458.1	Possible PhiRv2 prophage protein	75	RD13	NS	34, 38, 39

a aa, amino acids; DosR, dormancy survival regulon antigens; RD, region of difference; NS, nutrition starvation-associated antigens.

### Screening of high-frequency MHC-I and MHC-II alleles.

The high-frequency MHC-I- and MHC-II-restricted alleles in the Chinese population were selected using the Allele Frequency Net Database (http://www.allelefrequencies.net/default.asp). The parameter country was set as China, and the sample size was set as ≥500 (MHC-I) or ≥100 (MHC-II). The other parameters were default values. Alleles with an allele frequency of ≥0.10 were selected as the dominant MHC-I- and -II-restricted alleles in the Chinese population (47).

### Prediction and synthesis of Th1 and CTL epitopes.

The Th1 or CTL epitopes of candidate antigens of M. tuberculosis were predicted by the MHC-II or MHC-I binding module of the IEDB database as described in our previous studies (21, 48). In brief, the amino acid sequence of the target protein was filled in the “enter protein sequence (s) in FASTA format” column of the prediction page, the selected alleles in the Chinese population were uploaded to the “select MHC allele (s)” column, and other parameters were set to default values. The IEDB database has seven prediction methods for Th1 epitopes, including IEDB recommended, the Consensus method, the Combinatorial library, NN-align (netMHCII-2.2), SMM-align (netMHCII-1.1), Sturniolo, and NetMHCIIpan. By default, the system selects the IEDB recommended method for prediction, which preferentially uses NN-align, SMM-align, CombLib, and Sturniolo for prediction. Otherwise, the NetMHCIIpan method will be selected. Correspondingly, the IEDB database has nine prediction methods for CTL epitopes, including the artificial neural network (ANN), stabilized matrix method (SMM), SMM with a Peptide: MHC Binding Energy Covariance matrix (SMMPMBEC), Scoring Matrices derived from Combinatorial Peptide Libraries (Comblib_Sidney2008), Consensus, NetMHCpan, NetMHCcons, PickPocket, and NetMHCstabpan. By default, the system selects the IEDB recommended 2.19 method for CTL peptide prediction. This method preferentially uses Consensus, ANN, SMM, and CombLibif for prediction. When conditions are not allowed, the NetMHCIIpan method is used. The priority of these methods is Consensus > ANN > SMM > NetMHCpan > CombLib. Finally, the immunodominant peptides were synthesized by DGpeptides Co., Ltd. (Wuhan, Hubei Province, China).

### Construction of animal models with ATB and LTBI.

Female BALB/c mice at the age of 6 to 7 weeks were obtained from Vital River Laboratories (Beijing, China). Thirty mice were stratified according to their weight and randomly divided into three groups (10 mice per group), ensuring that the mice in each group were as close in weight as possible. ATB and LTBI mouse models were constructed as described in a previous study (49). In brief, mice in ATB and LTBI groups were infected with 3.6 × 10^5^ CFU of M. tuberculosis H37Rv (0.4 mL) via caudal vein injection. Four weeks later, mice in LTBI groups were treated with 0.12 g/L isoniazid and 8 g/L pyrazinamide in drinking water. At week 17, mice in the LTBI group were given normal drinking water without isoniazid and pyrazinamide. Furthermore, mice in the UC group were used as negative controls and fed generally without any treatments. Finally, at week 29, mice in each group were sacrificed, and their spleens and lungs were removed to evaluate the infection model. Briefly, the lungs were first weighed, and then the left lobe of the lung was homogenized in normal saline as described in our previous study (21). Subsequently, 0.1 mL of the diluted solution of each lung sample was inoculated onto a Lowenstein-Jensen medium plate (Baso Biotechnology Co., Ltd., Zhuhai, Guangdong Province, China) twice and incubated at 37°C. Twenty-eight days later, the CFUs for each plate were counted.

Furthermore, the right lobe of each lung was used for histopathological analysis as described in our previous studies (21, 48, 50). The lesion area rate was determined by using Image-Pro Plus software (version 6.0; Media Cybernetics, Inc., Bethesda, MD). A flow chart describing infection, treatment, activation, and evaluation in the animal models can be found in Fig. 1.

### Peptide dilution and preparation of peptide pools.

At 29 weeks after M. tuberculosis infection, mice in each group were sacrificed, and their spleens were obtained to prepare splenocytes suspension following our previous studies (21, 48). The synthetic peptide was dissolved and diluted in sterile Roswell Park Memorial Institute (RPMI) 1640 medium (Gibco/Thermo Fisher Technology, China) with a working concentration of 100 μg/mL. To verify the performance of multiple peptide combinations in diagnosing TB, we constructed five peptide pools: pool 1, pool 2, pool 3, pool 4, and pool 5. Pool 1 consists of four peptides from the Rv1737c antigen (Th1-Rv1737c-P3, Th1-Rv1737c-P5, CTL-Rv1737c-P1, and CTL-Rv1737c-P2), pool 2 consists of four peptides from Rv2031c antigen (Th1-Rv2031c-P1, Th1-Rv2031c-P2, CTL-Rv2031c-P1, and CTL-Rv2031c-P2), pool 3 consists of four peptides from Rv2626c antigen (Th1-Rv2626c-P1, Th1-Rv2626c-P2, CTL-Rv2626c-P1, and CTL-Rv2626c-P2), pool 4 consists of four peptides from Rv2659c antigen (Th1-Rv2659c-P1, Th1-Rv2659c-P2, CTL-Rv2659c-P1, and CTL-Rv2659c-P2), and pool 5 consists of four peptides from Rv2660c antigen (Th1-Rv2660c-P1, Th1-Rv2660c-P2, CTL-Rv2660c-P1, and CTL-Rv2660c-P2). The working concentration of each peptide in the pool is 100 μg/mL.

### ELISpot assay in mouse models.

About 100 μL of prepared splenocytes at a concentration of 3 × 10^6^/mL was added to each 96-well ELISpot plate. Then, 50 μL of phosphate-buffered saline (PBS; negative control), 50 μL of Th1 peptide (100 μg/mL), 50 μL of CTL peptide (100 μg/mL), 50 μL of peptide pool (100 μg/mL), or 50 μL of phytohemagglutinin (PHA; positive control, 50 μg/mL, lot SLBT1570 [Sigma-Aldrich, St. Louis, MO]) was added to the well, followed by incubation with splenocytes at 37°C for 24 h, respectively. Finally, the SFCs were determined by a using a mouse IFN-γ ELISpot Plus kit (catalog no. 3321-4APT-2; Mabtech, Nacka Strand, Sweden) according to the manufacturer’s instructions.

### ELISpot assay in TB or LTBI patients or in UCs.

Blood samples (5 ml) collected from patients with ATB (*n *=* *37) or LTBI (*n *=* *11) or from the UC group (*n *=* *62) were used to isolate PBMCs by using a human PBMC isolation kit (Solarbio, Beijing, China) according to the manufacturer’s instructions. Then, 100 μL of the isolated PBMCs (3 × 10^5^) was incubated with 50 μL of PBS (negative control), 50 μL of peptide pool (100 μg/mL), 50 μL of PHA (50 μg/mL, positive control), or 50 μL of recombinant CFP10-ESAT6 protein (CE protein, Gene-Optimal Science & Technology Co., Ltd., Shanghai, China), respectively, in a 96-well ELISpot plate at 37°C. After 24 h, SFCs were confirmed by using a human IFN-γ ELISpot PRO kit (catalog no. 3420-2APW-10; Mabtech) according to the manufacturer’s instructions.

### Th1/Th2/Th9/Th17/Th22/Treg cytokine analysis.

The mice in each group were killed at week 29 after M. tuberculosis infection, and the spleens were obtained to prepare splenocyte suspensions. A 100-μL volume of splenocytes at the concentration of 3 × 10^6^/mL was added to the well of a 96-well cell culture plate (Mabtech), followed by incubation with 50 μL of PBS (negative control), 50 μL of Th1 peptide (100 μg/mL), 50 μL of CTL peptide (100 μg/mL), 50 μL of peptide pool (100 μg/mL), or 50 μL of PHA (positive control, 50 μg/mL) at 37°C for 48 h. Then, after centrifugation at 500 × *g* for 10 min, the supernatant of each sample was collected to detect the levels of Th1/Th2/Th9/Th17/Th22/Treg cytokines (IFN-γ, IL-12p70, IL-13, IL-1β, IL-2, IL-4, IL-5, IL-6, TNF-α, GM-CSF, IL-18, IL-10, L-17A, IL-22, IL-23, IL-27, and IL-9) by using a Th1/Th2/Th9/Th17/Th22/Treg cytokine 17-Plex mouse ProcartaPlex panel (catalog no. EPX170-26087-901; Thermo Fisher, China) according to the manufacturer’s instructions.

### Statistical analysis.

The data in this study were analyzed using Prism 9.3.1 software (GraphPad, San Diego, CA). The data for lung weight, CFU in the left lobe of the lung, pathological lesions in the right lobe of the lung, and ELISpot assay results were analyzed using an ordinary one-way analysis of variance (ANOVA) test or a Kruskal-Wallis nonparametric test. Multiple comparisons were corrected with the Tukey or Dunn test according to the data normality and homogeneity of variances. The cytokine differences among the three groups were compared using the two-way ANOVA corrected with the Tukey test. Survival curves were compared using the Mantel-Cox logarithmic rank test. The diagnostic performance of candidate biomarkers was analyzed with ROC by using the Wilson/Brown test, and the AUC and 95% confidence intervals (CI) and sensitivity, as well as the specificity, were also determined. The data are presented as means ± the standard errors of the mean (SEM), and a *P* value of <0.05 was considered a significant difference.
