# Supplementary material for: Prediction of Th1 and Cytotoxic T Lymphocyte Epitopes of Mycobacterium tuberculosis and Evaluation of Their Potential in the Diagnosis of Tuberculosis in a Mouse Model and in Humans
Source: Microbiol Spectr. 2022 Aug 8;10(4):e01438-22. doi: 10.1128/spectrum.01438-22 (PMC9430503; doi:10.1128/spectrum.01438-22)
Supplement: Supplemental file 1 — Supplemental material. Download spectrum.01438-22-s0001.pdf, PDF file, 2.3 MB [file spectrum.01438-22-s0001.pdf]

Supplementary materials

Table S1 Twelve MHC I alleles and 25 MHC II alleles with high-frequency were identified in Chinese population

DRB1	DQA1/DQB1	DPA1/DPB1
HLA-DRB1*12:02	HLA-DQA1*05:01/DQB1*02:01	HLA-DPA1*01:03/DPB1*02:01
HLA-DRB1*09:01	HLA-DQA1*05:01/DQB1*03:01	HLA-DPA1*01/DPB1*04:01
HLA-DRB1*14:01	HLA-DQA1*03:01/DQB1*03:02	HLA-DPA1*03:01/DPB1*04:02
HLA-DRB1*07:01	HLA-DQA1*01:01/DQB1*05:01	HLA-DPA1*02:01/DPB1*05:01
HLA-DRB1*15:01	HLA-DQA1*01:02/DQB1*06:02	
HLA-DRB1*15:04	HLA-DQA1*02:01/DQB1*05:02	
HLA-DRB1*15:02	HLA-DQA1*06:01/DQB1*03:03	
HLA-DRB1*16:02	HLA-DQA1*03:01/DQB1*06:01	
HLA-DRB1*14:04		
HLA-DRB3*01:01		
HLA-DRB3*02:02		
HLA-DRB4*01:01		
HLA-DRB5*01:01		
**HLA-A**	**HLA-B**	**HLA-C**
A*11:01	B*15:01	C*01:02
A*02:01	B*15:02	C*07:02
A*24:02	B*15:11	C*03:04
A*33:03		C*06:02
A*30:01		

Table S2. The characteristics of Th1 dominant epitopes associated with LTBI-RD antigens

Antigen	Allele[1]	Start	End	Epitopes	Method[2]	Percentile_rank[3]
**Rv1737c**	HLA-DPA1*01:03/DPB1*02:01	292	306	QPPPEVWSAATFITL	Consensus (comb.lib./smm/nn)	0.01
	HLA-DPA1*01/DPB1*04:01	95	109	GSYALLVFFGLFLGV	Consensus (comb.lib./smm/nn)	0.03
	HLA-DQA1*06:01/DQB1*03:03<br>HLA-DQA1*03:01/DQB1*06:01	162	176	TTHAIVAAALASTAV	NetMHCIIpan	0.06
	HLA-DRB1*15:02<br>HLA-DPA1*01/DPB1*04:01	93	107	TMGSYALLVFFGLFL	sturniolo	0.07
	HLA-DRB1*09:01	193	207	DPVLPRLKAAARLPV	Consensus (comb.lib./smm/nn)	0.08
	HLA-DQA1*05:01/DQB1*03:01	164	178	HAIVAAALASTAVVA	Consensus (comb.lib./smm/nn)	0.18
	HLA-DQA1*01:02/DQB1*06:02	1	15	MRGQAANLVLATWIS	Consensus (comb.lib./smm/nn)	0.19
	HLA-DRB1*15:01	97	111	YALLVFFGLFLGVAG	Consensus (smm/nn/sturniolo)	0.23
	HLA-DQA1*03:01/DQB1*03:02	277	291	ASLAGTALLAFAAAL	Consensus (comb.lib./smm/nn)	0.64
	HLA-DRB5*01:01	233	247	ITTIYGFSTVDAGAR	Consensus (smm/nn/sturniolo)	0.78
	HLA-DRB3*01:01<br>HLA-DPA1*03:01/DPB1*04:02	42	56	EASLLVATPILVGAL	Consensus (comb.lib./smm/nn)	0.81
	HLA-DPA1*02:01/DPB1*05:01	91	105	AATMGSYALLVFFGL	Consensus (comb.lib./smm/nn)	0.85
	HLA-DRB1*14:01<br>HLA-DRB1*14:04<br>HLA-DRB3*02:02<br>HLA-DRB1*12:02	195	209	VLPRLKAAARLPVTW	NetMHCIIpan	1.16
	HLA-DQA1*01:01/DQB1*05:01	143	157	GTALSAFFTPRFVRW	Consensus (comb.lib./smm/nn)	1.34
	HLA-DQA1*02:01/DQB1*05:02	14	28	ISVVNFWAWNLIGPL	NetMHCIIpan	1.35
	HLA-DRB1*16:02	153	167	RFVRWFGLFTTHAIV	NetMHCIIpan	1.46

	HLA-DRB1*07:01	222	236	FVAFSNYLPTYITTI	Consensus (comb.lib./smm/nn)	1.93
	HLA-DQA1*05:01/DQB1*02:01	72	86	AMLIAVTLASILPVL	Consensus (comb.lib./smm/nn)	2.07
	HLA-DRB1*15:04	314	328	TGGVFAWVARRAPAA	NetMHCIIpan	2.39
	HLA-DRB4*01:01	173	187	STAVVAMVVLRDAPY	Consensus (comb.lib./smm/nn)	3.34
**Rv2031c**	HLA-DRB1*09:01	95	109	YGSFVRTVSLPVGAD	Consensus (comb.lib./smm/nn)	0.02
	HLA-DRB1*07:01<br>HLA-DRB1*15:02<br>HLA-DRB5*01:01	92	106	EFAYGSFVRTVSLPV	Consensus (comb.lib./smm/nn)	0.04
	HLA-DRB3*02:02<br>HLA-DRB1*14:01<br>HLA-DRB1*14:04<br>HLA-DRB1*15:04<br>HLA-DRB1*15:01<br>HLA-DRB1*16:02<br>HLA-DRB1*12:02	94	108	AYGSFVRTVSLPVGA	NetMHCIIpan	0.08
	HLA-DPA1*01:03/DPB1*02:01<br>HLA-DQA1*05:01/DQB1*03:01	88	102	DGRSEFAYGSFVRTV	Consensus (comb.lib./smm/nn)	0.12
	HLA-DPA1*01/DPB1*04:01	89	103	GRSEFAYGSFVRTVS	Consensus (comb.lib./smm/nn)	0.37
	HLA-DQA1*03:01/DQB1*03:02	14	28	LFPEFSELFAAFPSF	Consensus (comb.lib./smm/nn)	2.12
	HLA-DPA1*03:01/DPB1*04:02<br>HLA-DPA1*02:01/DPB1*05:01	90	104	RSEFAYGSFVRTVSL	Consensus (comb.lib./smm/nn)	2.42
	HLA-DQA1*05:01/DQB1*02:01	12	26	RSLFPEFSELFAAFP	Consensus (comb.lib./smm/nn)	2.56
	HLA-DQA1*02:01/DQB1*05:02	11	25	PRSLFPEFSELFAAF	NetMHCIIpan	2.73
	HLA-DQA1*06:01/DQB1*03:03<br>HLA-DQA1*03:01/DQB1*06:01	117	131	YDKGILTVSVAVSEG	NetMHCIIpan	3.68
	HLA-DRB3*01:01	63	77	DKDVDIMVRDGQLTI	Consensus (comb.lib./smm/nn)	4.37

	HLA-DQA1*01:02/DQB1*06:02	118	132	DKGILTVSVAVSEGK	Consensus (comb.lib./smm/nn)	4.76
	HLA-DRB4*01:01	96	110	GSFVRTVSLPVGADE	Consensus (comb.lib./smm/nn)	6.26
**Rv2626c**	HLA-DRB3*01:01	42	56	DDRLHGMLTDRDIVI	Consensus (comb.lib./smm/nn)	0.01
	HLA-DRB1*09:01<br>HLA-DRB3*02:02<br>HLA-DRB1*15:04<br>HLA-DRB1*14:04<br>HLA-DRB1*14:01	128	142	IVQFVKAICSPMALA	Consensus (comb.lib./smm/nn)	0.04
	HLA-DQA1*03:01/DQB1*03:02	76	90	DSIYYVDANASIQEM	Consensus (comb.lib./smm/nn)	0.57
	HLA-DRB1*15:01	126	140	HAIVQFVKAICSPMA	Consensus (smm/nn/sturniolo)	1.11
	HLA-DQA1*06:01/DQB1*03:03<br>HLA-DQA1*03:01/DQB1*06:01<br>HLA-DQA1*02:01/DQB1*05:02	77	91	SIYYVDANASIQEML	NetMHCIIpan	1.47
	HLA-DRB5*01:01<br>HLA-DRB1*12:02<br>HLA-DRB1*16:02<br>HLA-DRB4*01:01	127	141	AIVQFVKAICSPMAL	Consensus (smm/nn/sturniolo)	1.49
	HLA-DRB1*15:02	122	136	HLPEHAIVQFVKAIC	sturniolo	2.03
	HLA-DQA1*01:01/DQB1*05:01	73	87	LARDSIYYVDANASI	Consensus (comb.lib./smm/nn)	2.98
	HLA-DQA1*01:02/DQB1*06:02	15	29	VGEHETLTAAAQYMR	Consensus (comb.lib./smm/nn)	4.69
	HLA-DPA1*01:03/DPB1*02:01	120	134	ARHLPEHAIVQFVKA	Consensus (comb.lib./smm/nn)	5.03
	HLA-DQA1*05:01/DQB1*03:01	3	17	TARDIMNAGVTCVGE	Consensus (comb.lib./smm/nn)	5.04
	HLA-DRB1*07:01	125	139	EHAIVQFVKAICSPM	Consensus (comb.lib./smm/nn)	5.28
	HLA-DQA1*05:01/DQB1*02:01	79	93	YYVDANASIQEMLNV	Consensus (comb.lib./smm/nn)	5.52
	HLA-DPA1*03:01/DPB1*04:02<br>HLA-DPA1*02:01/DPB1*05:01	123	137	LPEHAIVQFVKAICS	Consensus (comb.lib./smm/nn)	7.83

Rv2659c	HLA-DRB1*16:02<br>HLA-DRB1*15:04	294	308	PSALYRMFYKARKAA	NetMHCIIpan	0.01
	HLA-DRB5*01:01<br>HLA-DRB1*14:04<br>HLA-DRB1*14:01<br>HLA-DRB1*12:02<br>HLA-DRB3*02:02	296	310	ALYRMFYKARKAAGR	Consensus (smm/nn/sturniolo)	0.02
	HLA-DRB1*15:02	190	204	KAMPDPYQAFVLMAA	sturniolo	0.12
	HLA-DRB1*09:01	194	208	DPYQAFVLMAAWLAM	Consensus (comb.lib./smm/nn)	0.18
	HLA-DQA1*01:02/DQB1*06:02	317	331	DLRHSGAVLAASTGA	Consensus (comb.lib./smm/nn)	0.18
	HLA-DPA1*01:03/DPB1*02:01<br>HLA-DQA1*01:01/DQB1*05:01	199	213	FVLMAAWLAMRYGEL	Consensus (comb.lib./smm/nn)	0.19
	HLA-DQA1*05:01/DQB1*03:01	318	332	LRHSGAVLAASTGAT	Consensus (comb.lib./smm/nn)	0.19
	HLA-DRB4*01:01	140	154	HSYSLLRAIMQTALA	Consensus (comb.lib./smm/nn)	0.39
	HLA-DRB3*01:01<br>HLA-DQA1*02:01/DQB1*05:02<br>HLA-DPA1*01/DPB1*04:01	196	210	YQAFVLMAAWLAMRY	Consensus (comb.lib./smm/nn)	0.4
	HLA-DQA1*06:01/DQB1*03:03	323	337	AVLAASTGATLAELM	NetMHCIIpan	0.53
	HLA-DQA1*03:01/DQB1*06:01	315	329	VHDLRHSGAVLAAST	NetMHCIIpan	0.61
	HLA-DRB1*15:01	193	207	PDPYQAFVLMAAWLA	Consensus (smm/nn/sturniolo)	0.79
	HLA-DQA1*05:01/DQB1*02:01	172	186	RRVHKIRPATLDELE	Consensus (comb.lib./smm/nn)	1.48
	HLA-DRB1*07:01	130	144	TAVGTPTMRAHSYSL	Consensus (comb.lib./smm/nn)	1.95
	HLA-DQA1*03:01/DQB1*03:02	39	53	KTFNAKIDAEAWLTD	Consensus (comb.lib./smm/nn)	2.41
	HLA-DPA1*03:01/DPB1*04:02	97	111	HYRKLLDNHILATFA	Consensus (comb.lib./smm/nn)	2.93
	HLA-DPA1*02:01/DPB1*05:01	292	306	LAPSALYRMFYKARK	Consensus (comb.lib./smm/nn)	5.38

**Rv2660c**	HLA-DQA1*03:01/DQB1*06:01<br>HLA-DQA1*06:01/DQB1*03:03	2	16	IAGVDQALAATGQAS	NetMHCIIpan	1.79
	HLA-DQA1*05:01/DQB1*03:01	16	30	SQRAAGASGGVTVGV	Consensus (comb.lib./smm/nn)	1.86
	HLA-DQA1*01:02/DQB1*06:02	33	47	GTEQRNLSVVAPSQF	Consensus (comb.lib./smm/nn)	4.02
	HLA-DRB1*09:01<br>HLA-DRB5*01:01	36	50	QRNLSVVAPSQFTFS	Consensus (comb.lib./smm/nn)	4.13
	HLA-DRB1*07:01	35	49	EQRNLSVVAPSQFTF	Consensus (comb.lib./smm/nn)	4.89
	HLA-DRB1*15:02	59	73	ETAGQSWCAILGLNQ	sturniolo	8.75

1. The HLA allele frequencies of Chinese population specific alleles were obtained from Allele Frequency Net Database (AFND, http://www.allelefrequencies.net/default.asp). The preferences of DRB1, DPA1, DPB1, DQA1, and DQB1 were set as following: Country = China, Sample Size >= 100, Allele Frequency >=0.10 will be considered. Finally, the following HLA alleles were chosen in this study: HLA-DRB1*12:02, HLA-DRB1*09:01, HLA-DRB1*14:01, HLA-DRB1*07:01, HLA-DRB1*15:01, HLA-DRB1*15:04, HLA-DRB1*15:02, HLA-DRB1*16:02, HLA-DRB1*14:04, HLA-DRB3*01:01, HLA-DRB3*02:02, HLA-DRB4*01:01, HLA-DRB5*01:01, HLA-DQA1*05:01/DQB1*02:01, HLA-DQA1*05:01/DQB1*03:01, HLA-DQA1*03:01/DQB1*03:02, HLA-DQA1*01:01/DQB1*05:01, HLA-DQA1*01:02/DQB1*06:02, HLA-DQA1*02:01/DQB1*05:02, HLA-DQA1*06:01/DQB1*03:03, HLA-DQA1*03:01/DQB1*06:01, HLA-DPA1*01:03/DPB1*02:01, HLA-DPA1*01/DPB1*04:01, HLA-DPA1*03:01/DPB1*04:02, and HLA-DPA1*02:01/DPB1*05:01. These data were accessed on September 18, 2018.

2. The prediction method list box allows choosing between seven currently implemented MHC class II binding prediction methods: IEDB recommended, Consensus method, Combinatorial library, NN-align (netMHCII-2.2), SMM-align (netMHCII-1.1), Sturniolo, and NetMHCIIpan. The default selection IEDB Recommended is provided. Based

on availability of predictors and previously observed predictive performance, this selection tries to use the best possible method for a given MHC molecule. The selection IEDB Recommended uses the Consensus approach, combining NN-align, SMM-align, CombLib and Sturniolo if any corresponding predictor is available for the molecule, otherwise NetMHCIIpan is used.

3. For each peptide, a percentile rank for each of the three methods (combinatorial library, SMM_align and Sturniolo) is generated by comparing the peptide's score against the scores of five million random 15 mers selected from SWISSPROT database. A small numbered percentile rank indicates high affinity. The median percentile rank of the three methods were then used to generate the rank for consensus method.

Table S3. The characteristics of CTL dominant epitopes associated with LTBI-RD antigens

Antigen	Allele[1]	Start	End	length	Peptide	Method[2]	Percentile_rank[3]
**Rv1737c**	HLA-C*01:02	268	276	9	RIAPRHVVL	netmhcpan	0.01
	HLA-A*33:03	378	386	9	CTYTALHAR	netmhcpan	0.02
	HLA-B*15:11	179	187	9	MVVLRDAPY	netmhcpan	0.02
	HLA-C*03:04	86	94	9	LAVGVAATM	netmhcpan	0.03
	HLA-C*07:02	124	132	9	WYQPARRGF	Consensus (ann/smm)	0.07
	HLA-C*06:02	1	9	9	MRGQAANLV	Consensus (ann/smm)	0.11
	HLA-A*24:02	231	239	9	TYITTIYGF	Consensus (ann/smm)	0.11
	HLA-B*15:01	178	187	10	AMVVLRDAPY	Consensus (ann/smm)	0.18
	HLA-A*02:01	44	53	10	SLLVATPILV	Consensus (ann/smm)	0.29
	HLA-B*15:02	213	222	10	FLYAIVFGGF	ann	0.34
	HLA-A*11:01	145	153	9	ALSAFFTPR	Consensus (ann/smm)	0.55
	HLA-A*30:01	198	207	10	RLKAAARLPV	Consensus (ann/smm)	0.83
**Rv2031c**	HLA-C*03:04	23	31	9	AAFPSFAGL	netmhcpan	0.04
	HLA-A*33:03	92	100	9	EFAYGSFVR	netmhcpan	0.05
	HLA-C*01:02<br>HLA-A*02:01	13	21	9	SLFPEFSEL	netmhcpan	0.06
	HLA-B*15:11	10	18	9	HPRSLFPEF	netmhcpan	0.15
	HLA-A*24:02	14	22	9	LFPEFSELF	Consensus (ann/smm)	0.17
	HLA-B*15:01	20	28	9	ELFAAFPSF	Consensus<br>(ann/comblib_sidney2008/smm)	0.3

	HLA-C*06:02<br>HLA-C*07:02	49	57	9	GRYEVRAEL	Consensus (ann/smm)	0.39
	HLA-A*11:01	123	132	10	TVSVAVSEGK	Consensus (ann/smm)	0.52
	HLA-A*30:01	98	106	9	FVRTVSLPV	Consensus (ann/comblib_sidney2008/smm)	0.9
	HLA-B*15:02	87	95	9	FDGRSEFAY	Consensus (ann/smm)	1.49
**Rv2626c**	HLA-C*03:04<br>HLA-C*01:02	133	141	9	KAICSPMAL	netmhcpan	0.02
	HLA-B*15:11	131	139	9	FVKAICSPM	netmhcpan	0.03
	HLA-A*33:03<br>HLA-A*11:01	21	29	9	LTAAAQYMR	netmhcpan	0.09
	HLA-A*24:02	79	87	9	YYVDANASI	Consensus (ann/smm)	0.22
	HLA-A*30:01	73	81	9	LARDSIYYV	Consensus (ann/comblib_sidney2008/smm)	0.4
	HLA-C*07:02	100	108	9	RRVPVISEH	Consensus (ann/smm)	0.46
	HLA-A*02:01	72	81	10	ELARDSIYYV	Consensus (ann/smm)	0.48
	HLA-B*15:02	27	36	10	YMREHDIGAL	ann	0.91
	HLA-B*15:01	71	80	10	GELARDSIYY	Consensus (ann/smm)	1.25
	HLA-C*06:02	120	128	9	ARHLPEHAI	Consensus (ann/smm)	1.65
**Rv2659c**	HLA-B*15:11	202	210	9	MAAWLAMRY	netmhcpan	0.02
	HLA-C*03:04<br>HLA-C*01:02	325	333	9	LAASTGATL	netmhcpan	0.02
	HLA-A*33:03	201	209	9	LMAAWLAMR	netmhcpan	0.05
	HLA-A*11:01	295	303	9	SALYRMFYK	Consensus (ann/smm)	0.11

	HLA-C*06:02	208	216	9	MRYGELTEL	Consensus (ann/smm)	0.12
	HLA-C*07:02	54	62	9	RRREIDRQL	Consensus (ann/smm)	0.13
	HLA-A*30:01	93	101	9	RTRAHYRKL	Consensus(ann/comblib_siney2008/smm)	0.2
	HLA-B*15:02<br>HLA-B*15:01	199	208	10	FVLMAAWLAM	ann	0.29
	HLA-A*02:01	100	108	9	KLLDNHILA	Consensus(ann/comblib_siney2008/smm)	0.8
	HLA-A*24:02	191	199	9	AMPDPYQAF	Consensus (ann/smm)	1.1
**Rv2660c**	HLA-B*15:11<br>HLA-B*15:02	47	56	10	FTFSSRSPDF	netmhcpan	0.07
	HLA-C*01:02<br>HLA-C*03:04<br>HLA-C*07:02<br>HLA-A*24:02	41	49	9	VVAPSQFTF	netmhcpan	0.17
	HLA-B*15:01	16	24	9	SQRAAGASG	Consensus<br>(ann/comblib_sidney2008/smm)	0.7
	HLA-A*33:03<br>HLA-A*11:01	43	52	10	APSQFTFSSR	netmhcpan	0.86
	HLA-A*30:01	15	24	10	ASQRAAGASG	Consensus (ann/smm)	1.3
	HLA-C*06:02	51	59	9	SRSPDFVDE	Consensus (ann/smm)	3.45
	HLA-A*02:01	62	71	10	GQSWCAILGL	Consensus (ann/smm)	4.1

1. The HLA allele frequencies of Chinese population specific alleles were obtained from Allele Frequency Net Database (AFND, http://www.allelefrequencies.net/default.asp).

The preferences of HLA-A, HLA-B, and HAL-C were set as following: Country = China, Sample Size >= 500, Allele Frequency >=0.10 will be considered. Finally, the

following HLA alleles were chosen in this study: HLA-A*11:01, HLA-A*02:01, HLA-A*24:02, HLA-A*33:03, HLA-A*30:01, HLA-B*15:01, HLA-B*15:02, HLA-B*15:11, HLA-C*01:02, HLA-C*07:02, HLA-C*03:04, and HLA-C*06:02. These data were accessed on September 30, 2018.

2. The prediction method list box allows choosing from a number of MHC class I binding prediction methods: Artificial neural network (ANN), Stabilized matrix method (SMM), SMM with a Peptide: MHC Binding Energy Covariance matrix (SMMPMBEC), Scoring Matrices derived from Combinatorial Peptide Libraries (Comblib_Sidney2008), Consensus, NetMHCpan, NetMHCcons, PickPocket and NetMHCstabpan. IEDB recommended is the default prediction method selection. Based on availability of predictors and previously observed predictive performance, this selection tries to use the best possible method for a given MHC molecule. Currently for peptide: MHC-I binding prediction, for a given MHC molecule, IEDB Recommended uses the Consensus method consisting of ANN, SMM, and CombLibif any corresponding predictor is available for the molecule. Otherwise, NetMHCpan is used. This choice was motivated by the expected predictive performance of the methods in decreasing order: Consensus > ANN > SMM > NetMHCpan > CombLib.

3. In addition to the IC50 values for each peptide, a percentile rank is generated by comparing the peptide's IC50against those of a set of random peptides from SWISSPROT database. A small numbered percentile rank indicates high affinity. For the 'consensus' and 'IEDB recommended' methods, the median percentile rank of the methods used is reported as the representative percentile rank.

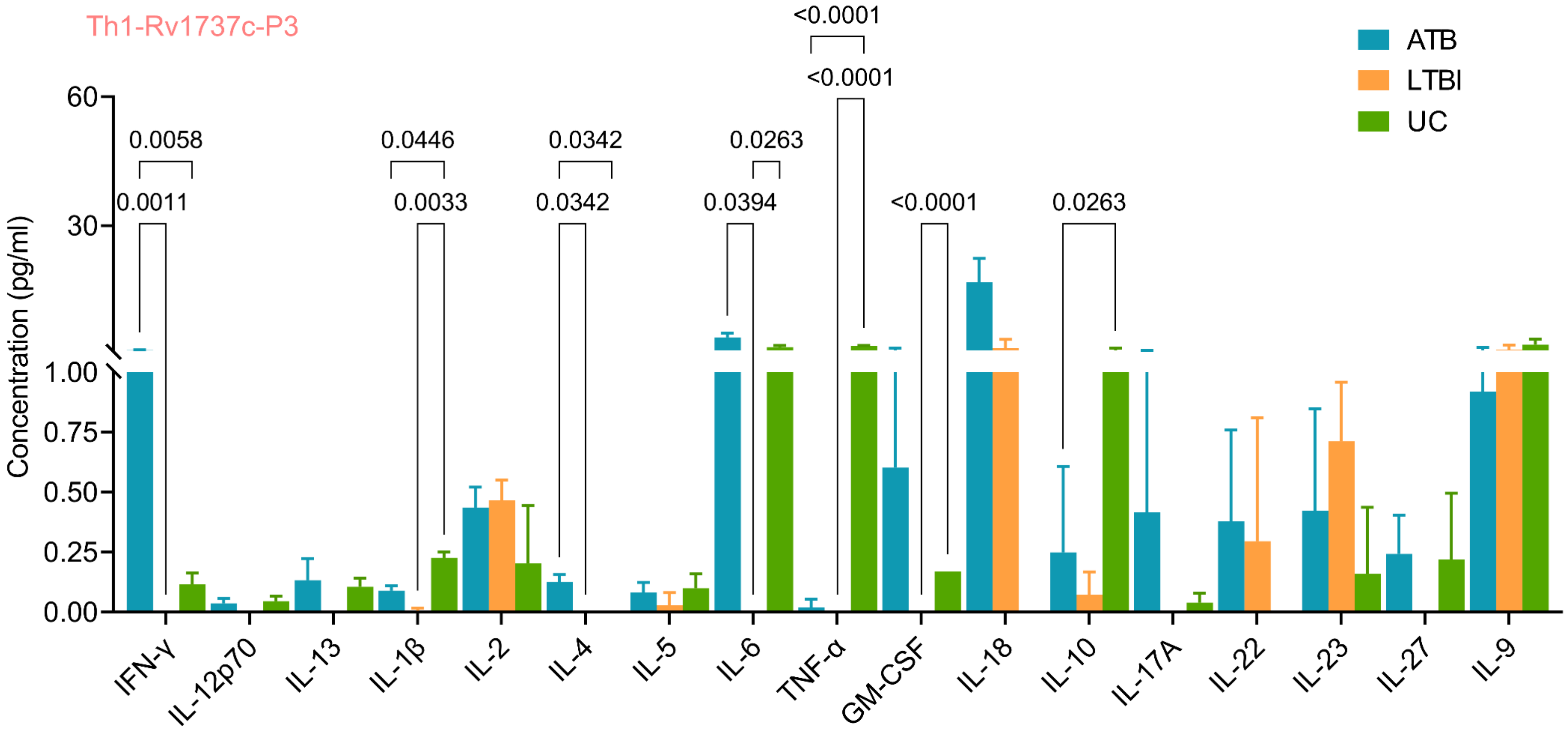

Th1-Rv1737c-P3
ATB
LTBI
UC
Concentration (pg/ml)
60
30
1.00
0.75
0.50
0.25
0.00
0.0058
0.0011
0.0446
0.0033
0.0342
0.0342
0.0263
0.0394
<0.0001
<0.0001
<0.0001
0.0263
IFN-γ
IL-12p70
IL-13
IL-1β
IL-2
IL-4
IL-5
IL-6
TNF-α
GM-CSF
IL-18
IL-10
IL-17A
IL-22
IL-23
IL-27
IL-9

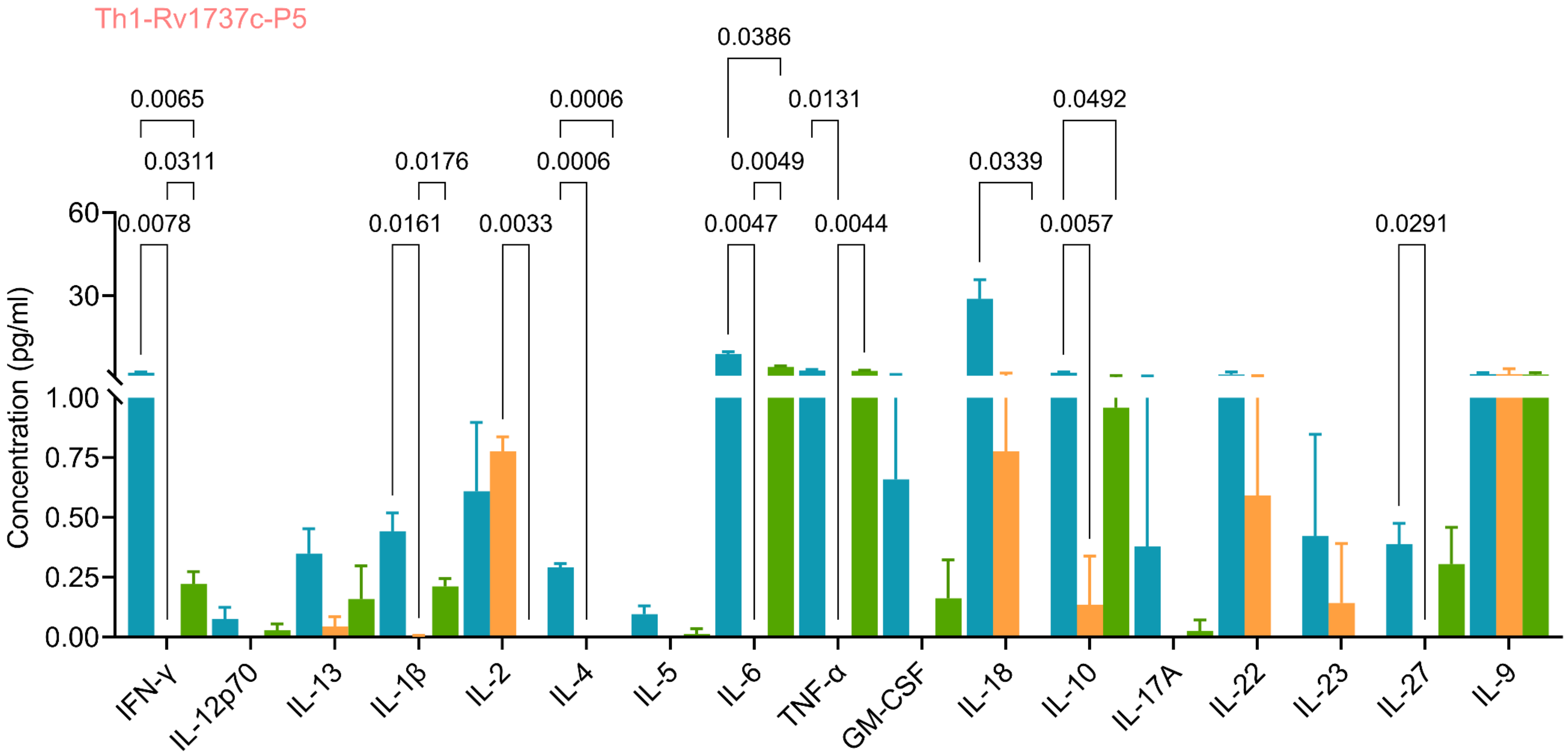

Th1-Rv1737c-P5
Concentration (pg/ml)
60
30
1.00
0.75
0.50
0.25
0.00
0.0065
0.0311
0.0078
0.0176
0.0161
0.0033
0.0006
0.0006
0.0386
0.0049
0.0047
0.0131
0.0044
0.0339
0.0492
0.0057
0.0291
IFN-γ
IL-12p70
IL-13
IL-1β
IL-2
IL-4
IL-5
IL-6
TNF-α
GM-CSF
IL-18
IL-10
IL-17A
IL-22
IL-23
IL-27
IL-9

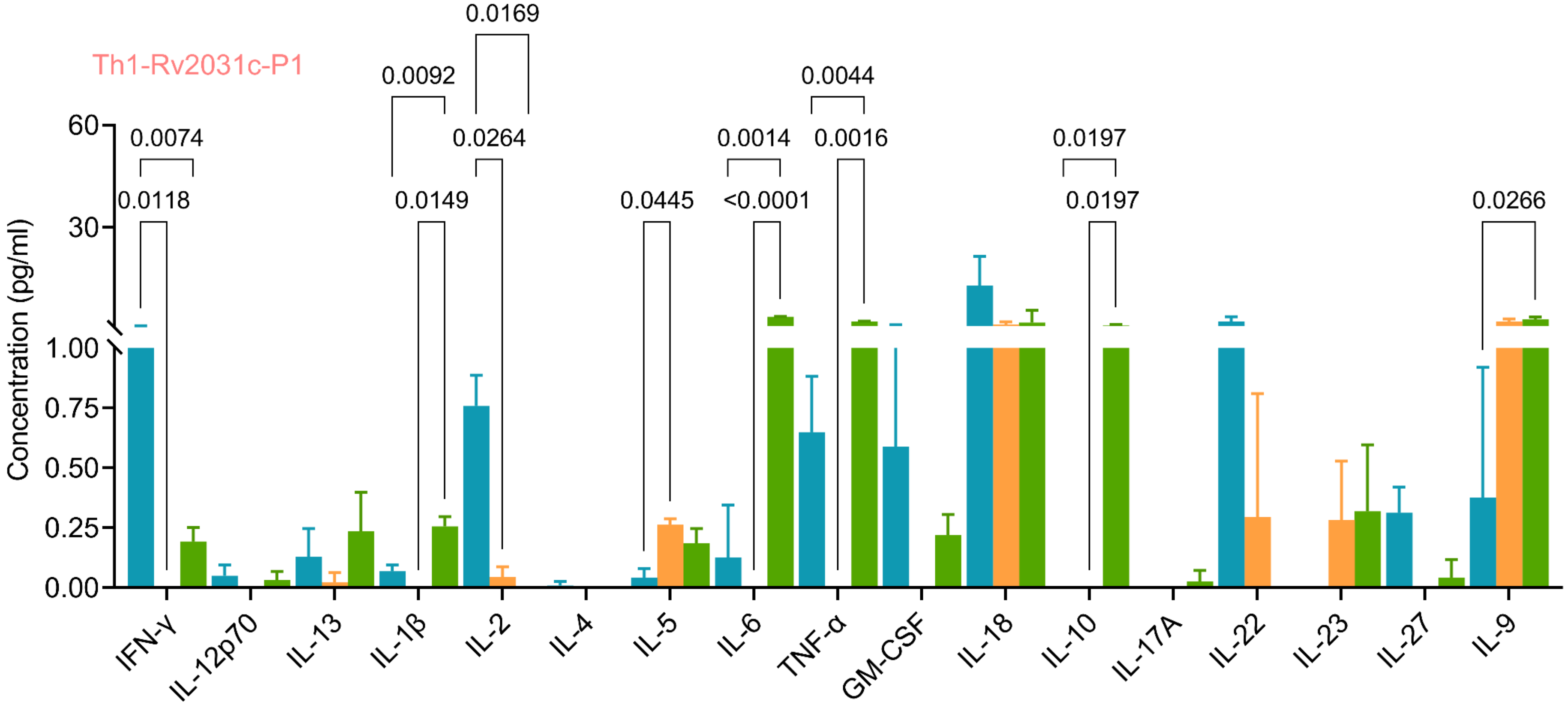

Th1-Rv2031c-P1
Concentration (pg/ml)
60
30
1.00
0.75
0.50
0.25
0.00
0.0074
0.0118
0.0092
0.0149
0.0169
0.0264
0.0445
0.0014
<0.0001
0.0044
0.0016
0.0197
0.0197
0.0266
IFN-γ
IL-12p70
IL-13
IL-1β
IL-2
IL-4
IL-5
IL-6
TNF-α
GM-CSF
IL-18
IL-10
IL-17A
IL-22
IL-23
IL-27
IL-9

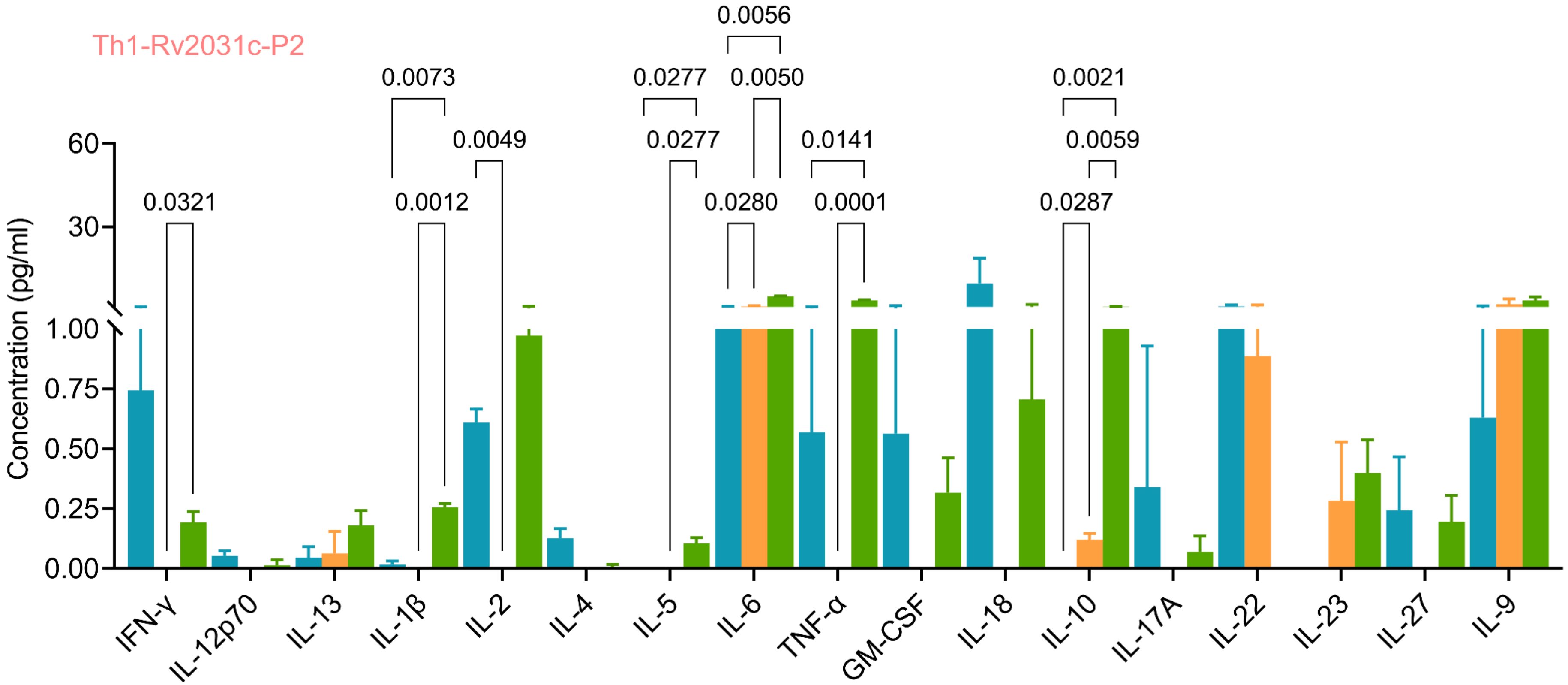
Th1-Rv2031c-P2
Concentration (pg/ml)
60
30
1.00
0.75
0.50
0.25
0.00
0.0321
0.0073
0.0012
0.0049
0.0277
0.0277
0.0056
0.0050
0.0280
0.0141
0.0001
0.0021
0.0059
0.0287
IFN-γ
IL-12p70
IL-13
IL-1β
IL-2
IL-4
IL-5
IL-6
TNF-α
GM-CSF
IL-18
IL-10
IL-17A
IL-22
IL-23
IL-27
IL-9

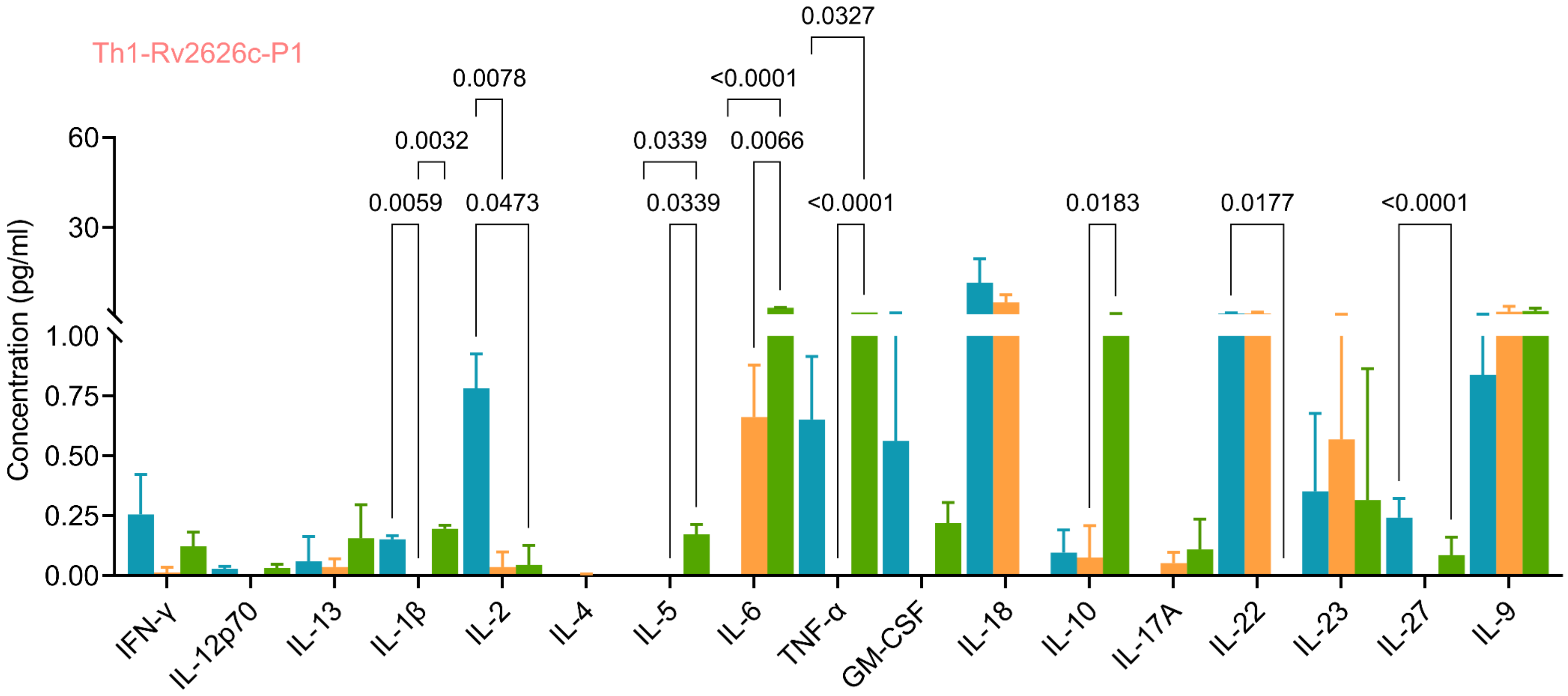
Th1-Rv2626c-P1
Concentration (pg/ml)
60
30
1.00
0.75
0.50
0.25
0.00
0.0327
0.0078
<0.0001
0.0032
0.0339
0.0066
0.0059
0.0473
0.0339
<0.0001
0.0183
0.0177
<0.0001
IFN-γ
IL-12p70
IL-13
IL-1β
IL-2
IL-4
IL-5
IL-6
TNF-α
GM-CSF
IL-18
IL-10
IL-17A
IL-22
IL-23
IL-27
IL-9

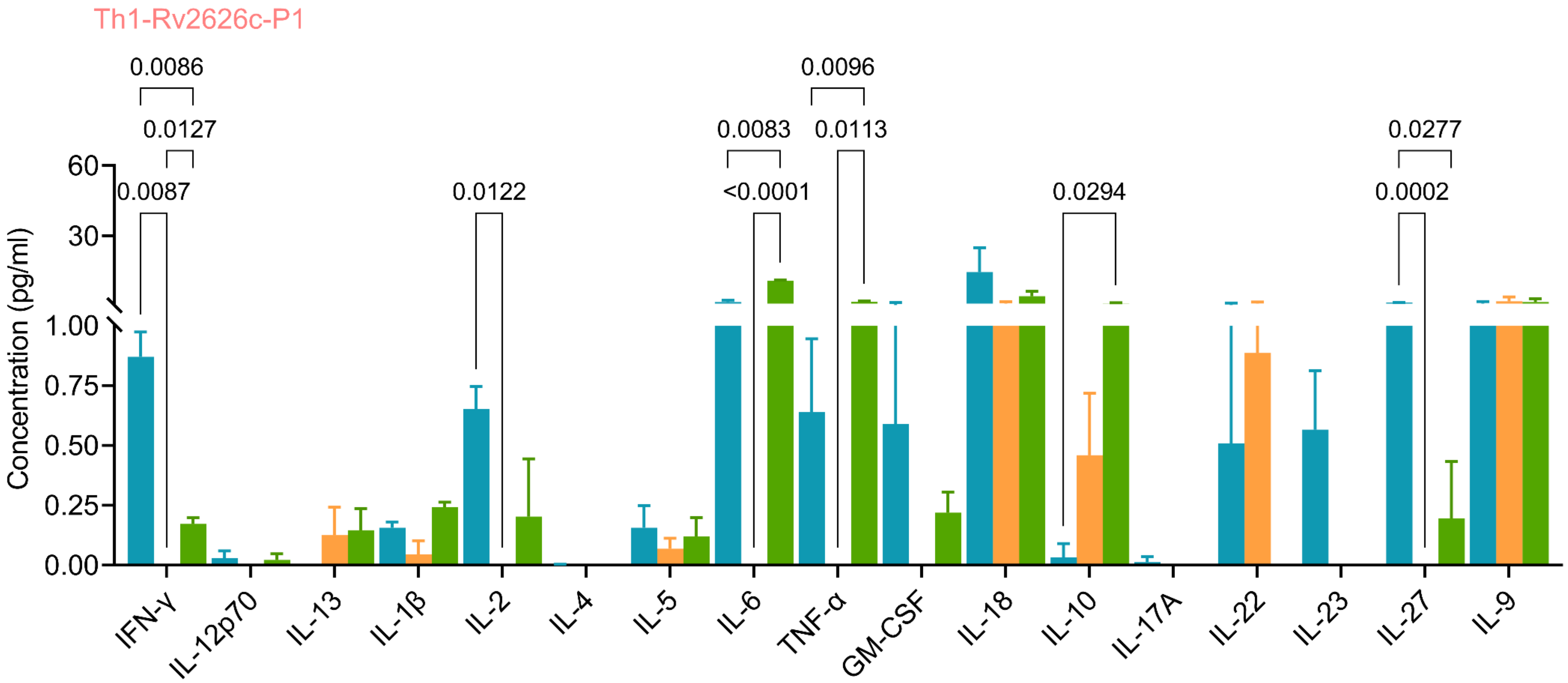
Th1-Rv2626c-P1
0.0086
0.0127
0.0087
0.0122
0.0083
<0.0001
0.0096
0.0113
0.0294
0.0277
0.0002
Concentration (pg/ml)
60
30
1.00
0.75
0.50
0.25
0.00
IFN-γ
IL-12p70
IL-13
IL-1β
IL-2
IL-4
IL-5
IL-6
TNF-α
GM-CSF
IL-18
IL-10
IL-17A
IL-22
IL-23
IL-27
IL-9

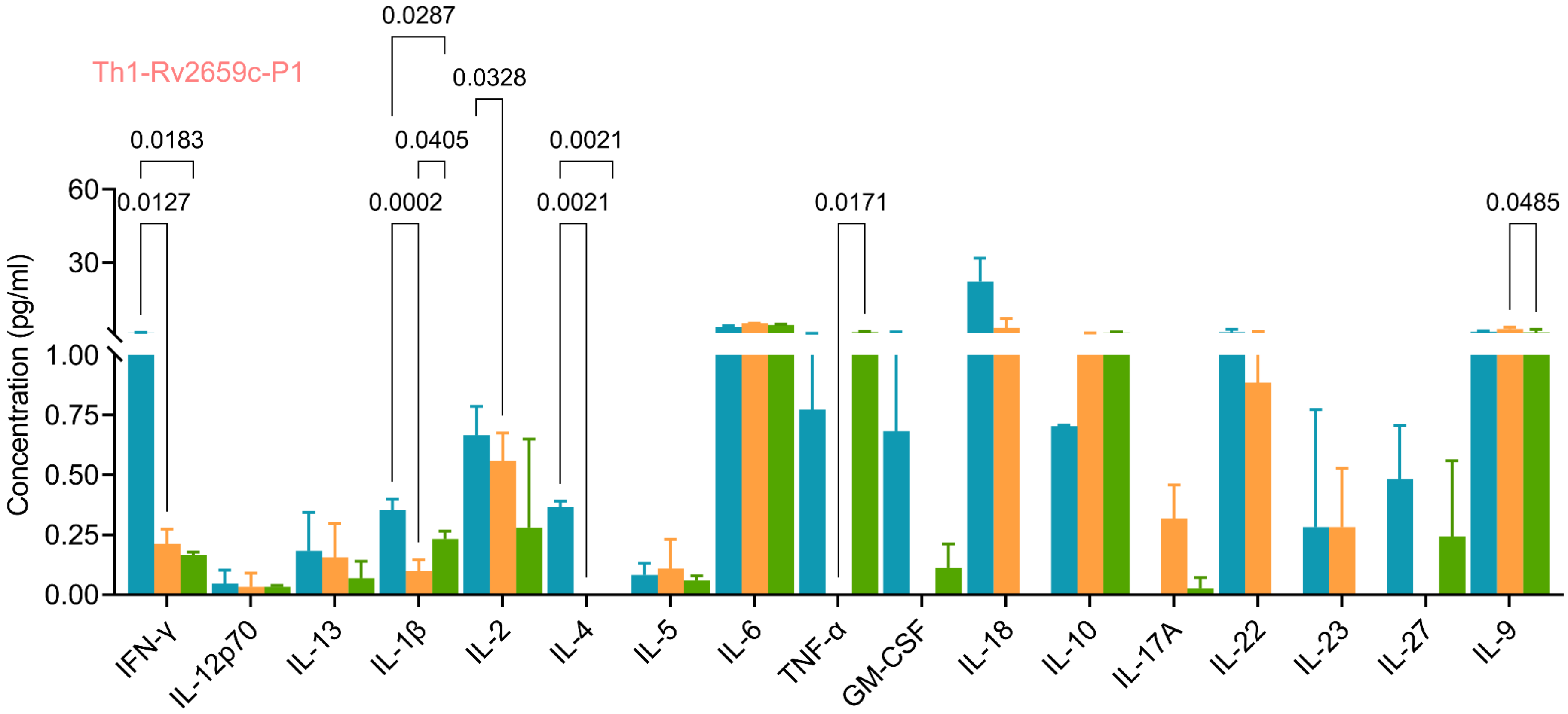

Th1-Rv2659c-P1
Concentration (pg/ml)
60
30
1.00
0.75
0.50
0.25
0.00
0.0183
0.0127
0.0287
0.0328
0.0405
0.0002
0.0021
0.0021
0.0171
0.0485
IFN-γ
IL-12p70
IL-13
IL-1β
IL-2
IL-4
IL-5
IL-6
TNF-α
GM-CSF
IL-18
IL-10
IL-17A
IL-22
IL-23
IL-27
IL-9

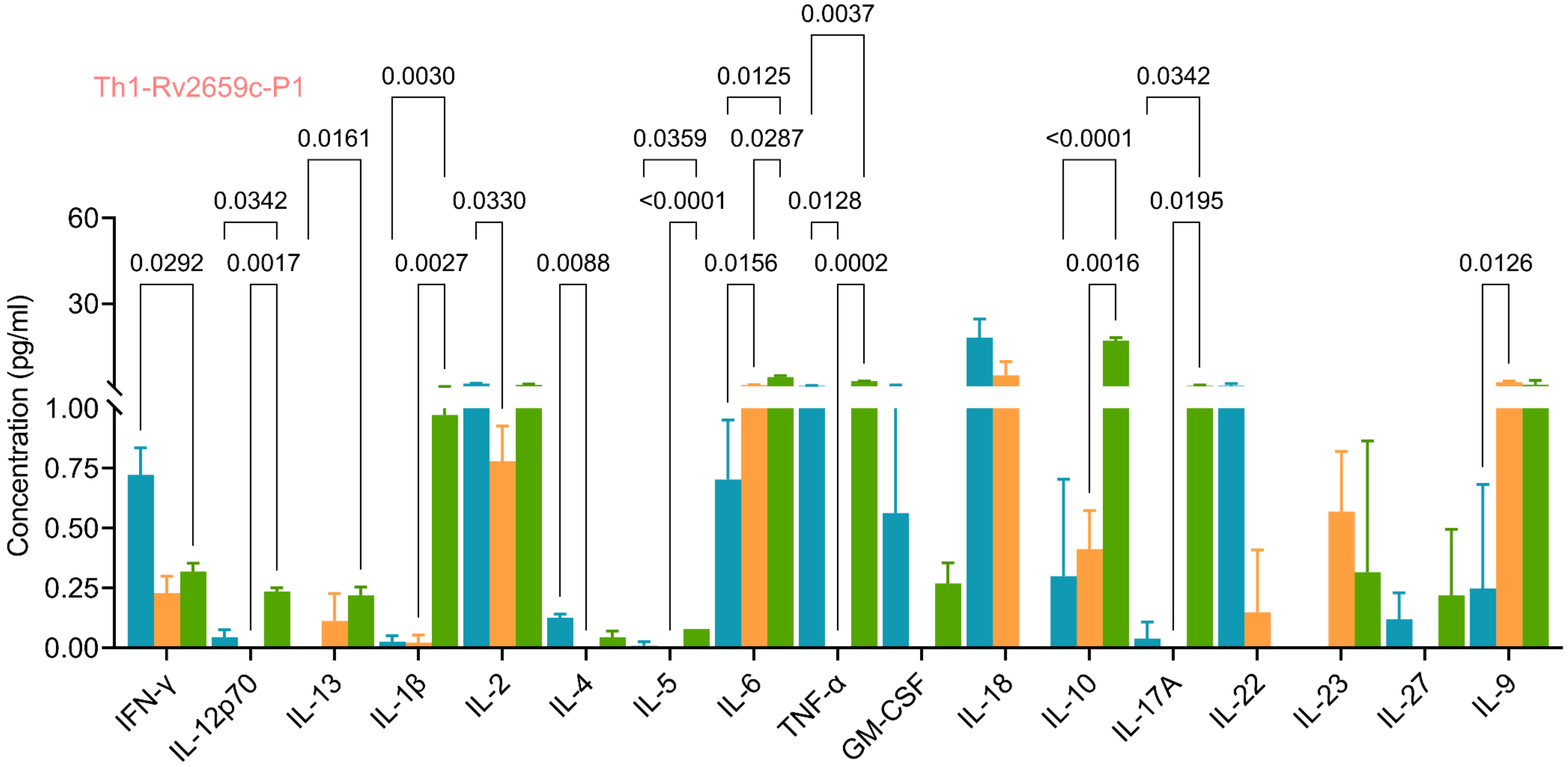

Th1-Rv2659c-P1
Concentration (pg/ml)
60
30
1.00
0.75
0.50
0.25
0.00
0.0292
0.0342
0.0017
0.0161
0.0030
0.0027
0.0330
0.0088
0.0359
<0.0001
0.0125
0.0287
0.0156
0.0037
0.0128
0.0002
<0.0001
0.0016
0.0342
0.0195
0.0126
IFN-γ
IL-12p70
IL-13
IL-1β
IL-2
IL-4
IL-5
IL-6
TNF-α
GM-CSF
IL-18
IL-10
IL-17A
IL-22
IL-23
IL-27
IL-9

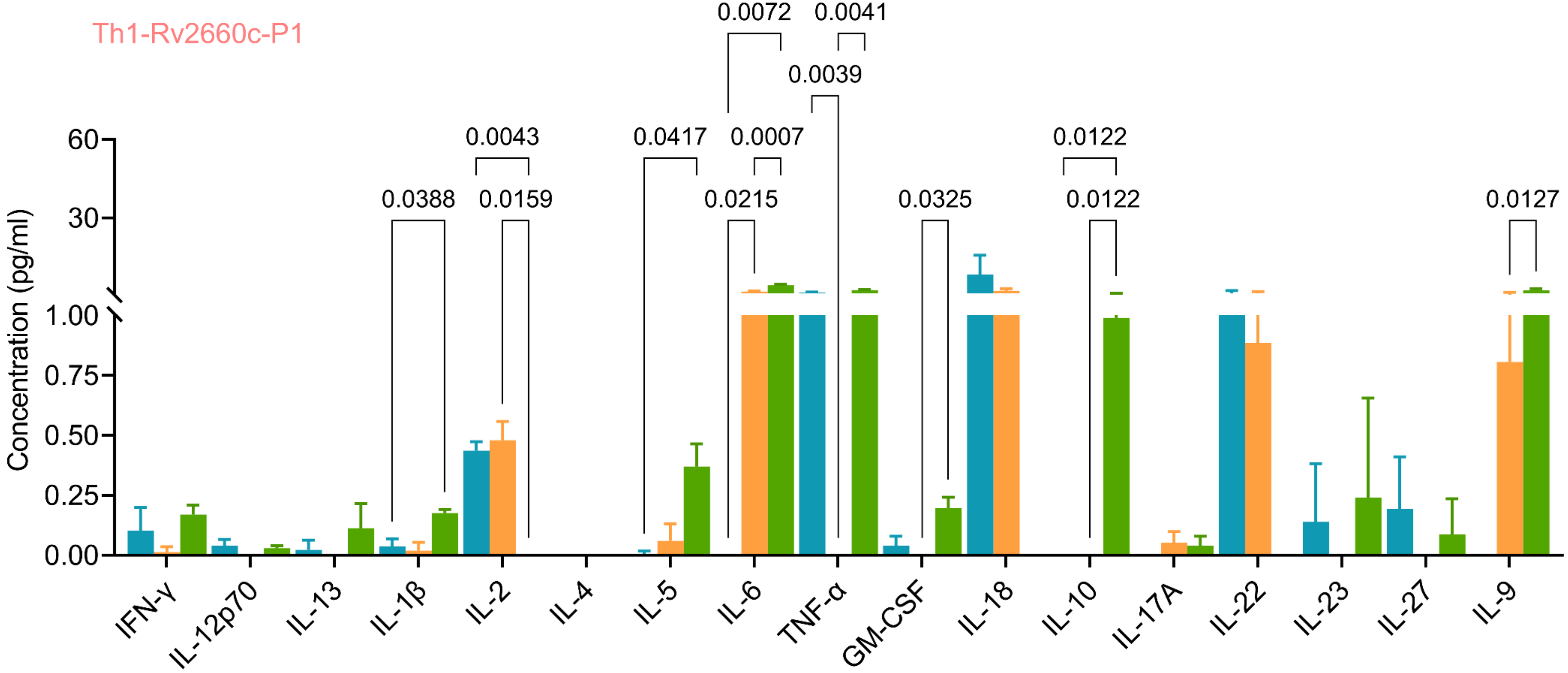
Th1-Rv2660c-P1
Concentration (pg/ml)
60
30
1.00
0.75
0.50
0.25
0.00
0.0072
0.0041
0.0039
0.0043
0.0417
0.0007
0.0122
0.0388
0.0159
0.0215
0.0325
0.0122
0.0127
IFN-γ
IL-12p70
IL-13
IL-1β
IL-2
IL-4
IL-5
IL-6
TNF-α
GM-CSF
IL-18
IL-10
IL-17A
IL-22
IL-23
IL-27
IL-9

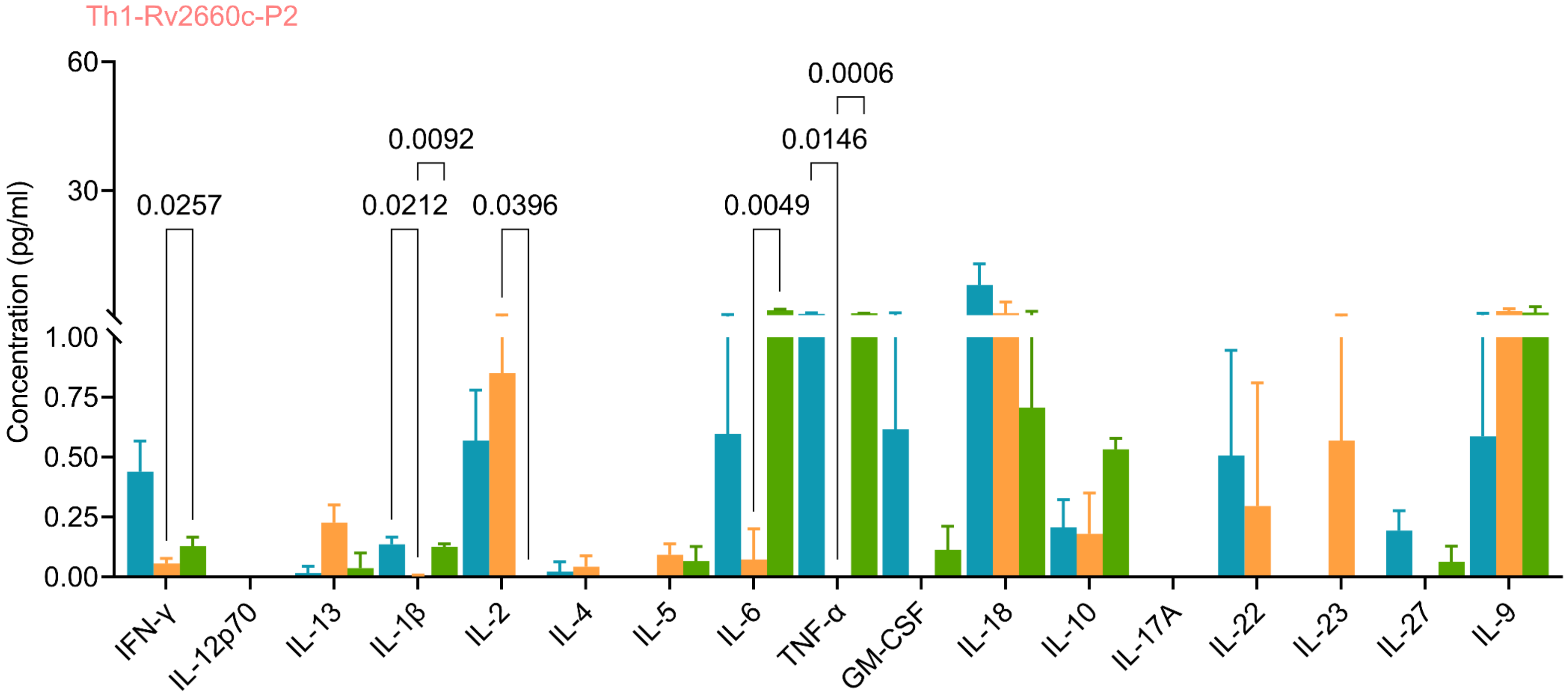

Figure S1. Detailed information of cytokines induced by Th1 dominant peptides in mice. The splenocytes collected from mice in ATB, LTBI, and UC groups were stimulated with the ten Th1 dominant peptides for 48 hours. The levels of IFN-γ, IL-12p70, IL-13, IL-1β, IL-2, IL-4, IL-5, IL-6, TNF-α, GM-CSF, IL-18, IL-10, L-17A, IL-22, IL-23, IL-27, and IL-9 cytokines in the supernatant were detected with a Mouse Th1/Th2/Th9/Th17/Th22/Treg Cytokine Kit. The cytokine differences among the three

groups were compared with the two-way analysis of variance (ANOVA) corrected with the Tukey test. All data were shown as mean + SEM (n = 3). $P < 0.05$ was considered significantly different.

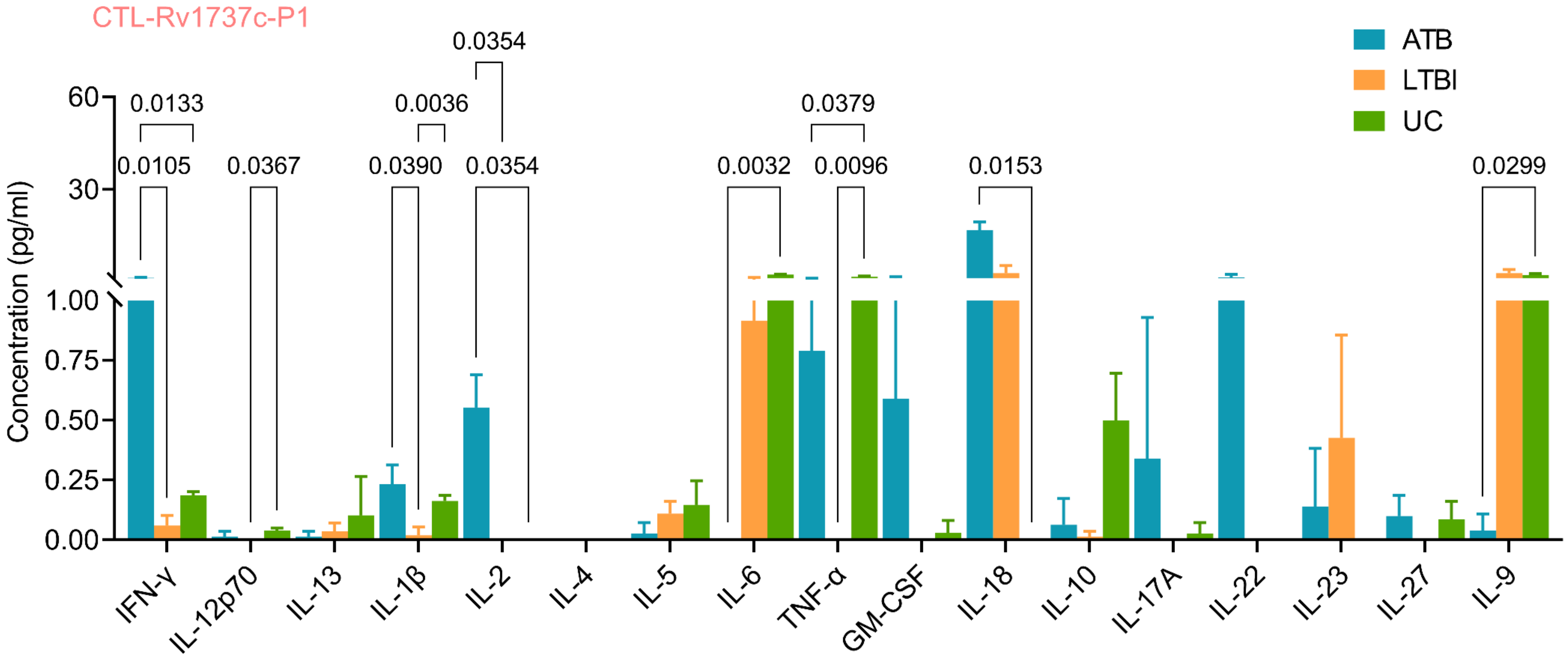

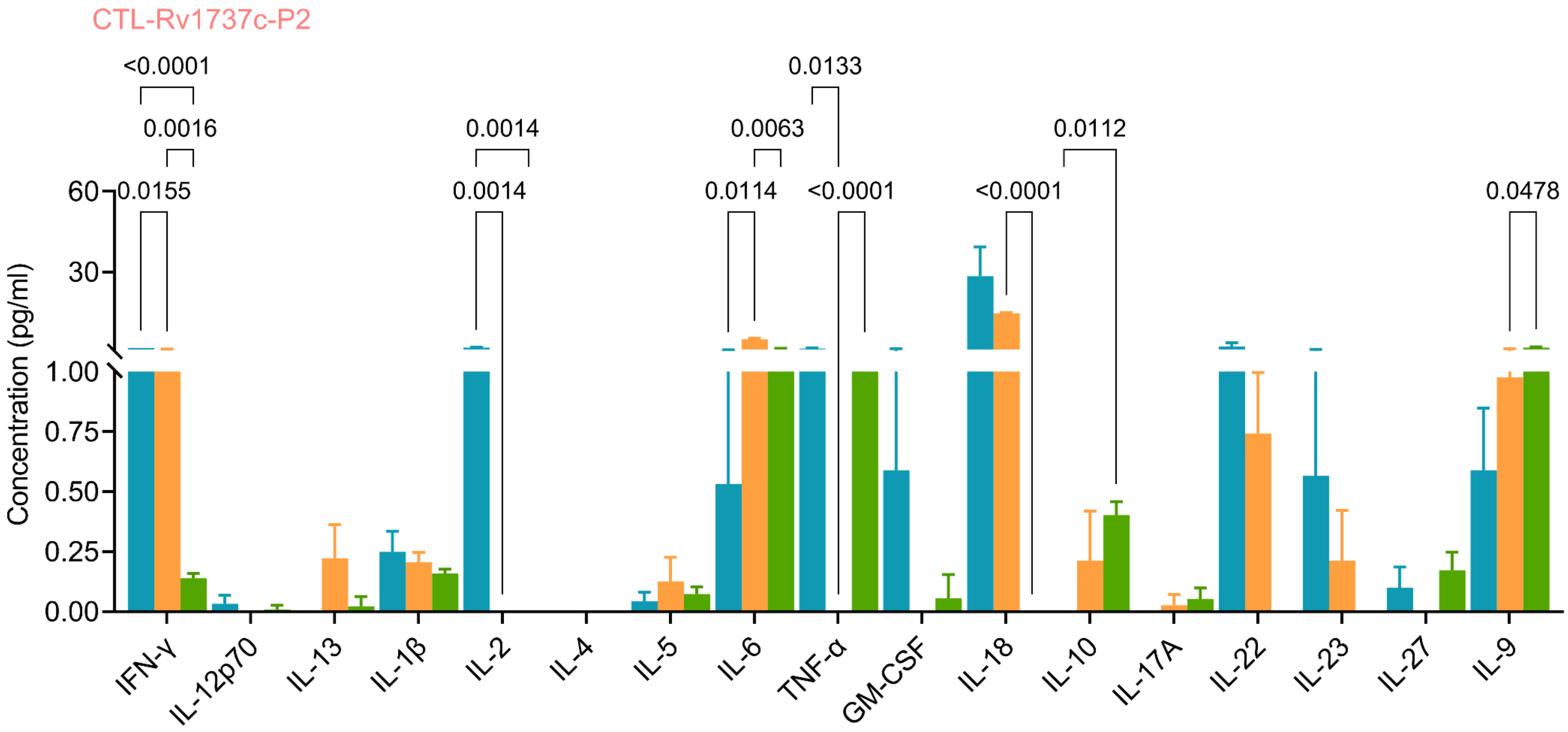
CTL-Rv1737c-P2
Concentration (pg/ml)
60
30
1.00
0.75
0.50
0.25
0.00
<0.0001
0.0016
0.0155
0.0014
0.0014
0.0063
0.0114
0.0133
<0.0001
<0.0001
0.0112
0.0478
IFN-γ
IL-12p70
IL-13
IL-1β
IL-2
IL-4
IL-5
IL-6
TNF-α
GM-CSF
IL-18
IL-10
IL-17A
IL-22
IL-23
IL-27
IL-9

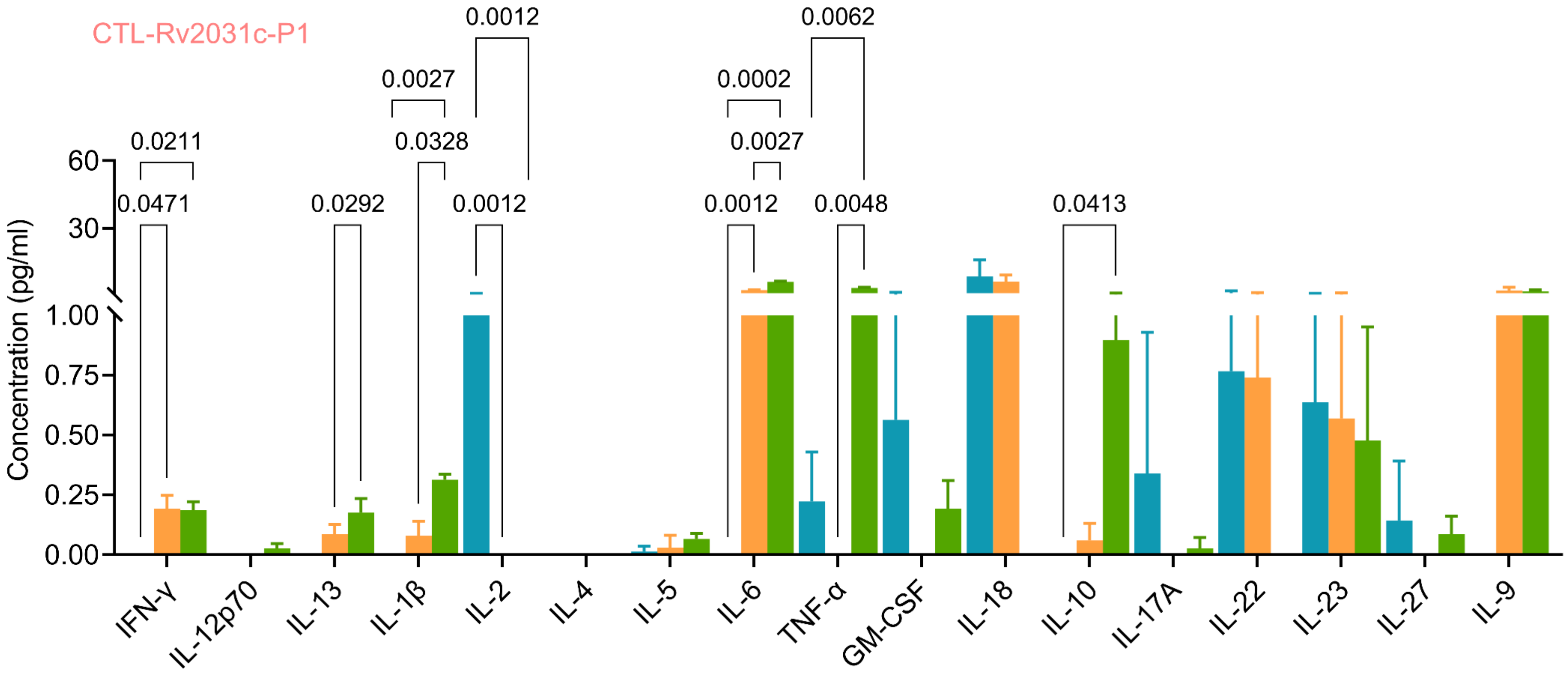
CTL-Rv2031c-P1
Concentration (pg/ml)
60
30
1.00
0.75
0.50
0.25
0.00
0.0211
0.0471
0.0292
0.0027
0.0328
0.0012
0.0012
0.0002
0.0027
0.0012
0.0062
0.0048
0.0413
IFN-γ
IL-12p70
IL-13
IL-1β
IL-2
IL-4
IL-5
IL-6
TNF-α
GM-CSF
IL-18
IL-10
IL-17A
IL-22
IL-23
IL-27
IL-9

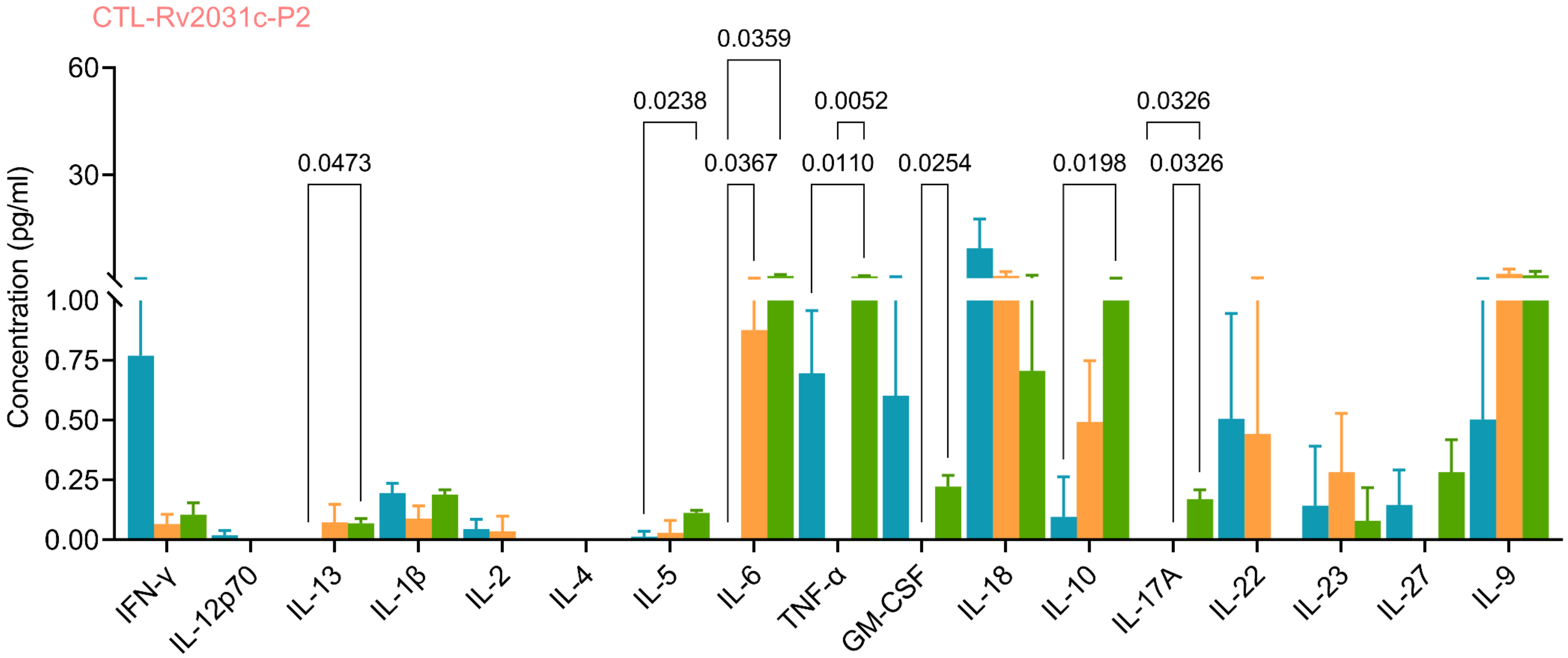

CTL-Rv2031c-P2
Concentration (pg/ml)
60
30
1.00
0.75
0.50
0.25
0.00
0.0473
0.0238
0.0359
0.0367
0.0052
0.0110
0.0254
0.0198
0.0326
0.0326
IFN-γ
IL-12p70
IL-13
IL-1β
IL-2
IL-4
IL-5
IL-6
TNF-α
GM-CSF
IL-18
IL-10
IL-17A
IL-22
IL-23
IL-27
IL-9

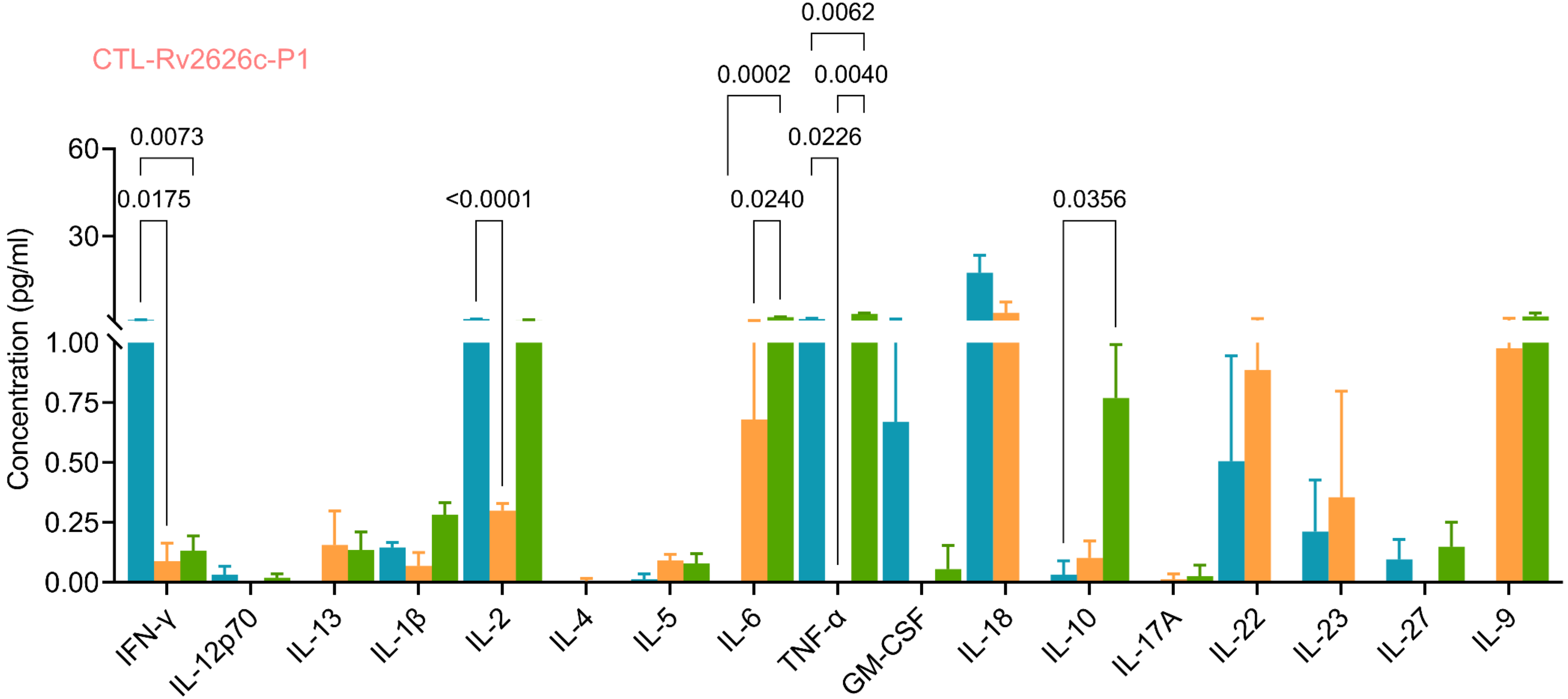

CTL-Rv2626c-P1
Concentration (pg/ml)
60
30
1.00
0.75
0.50
0.25
0.00
0.0073
0.0175
<0.0001
0.0002
0.0240
0.0062
0.0040
0.0226
0.0356
IFN-γ
IL-12p70
IL-13
IL-1β
IL-2
IL-4
IL-5
IL-6
TNF-α
GM-CSF
IL-18
IL-10
IL-17A
IL-22
IL-23
IL-27
IL-9

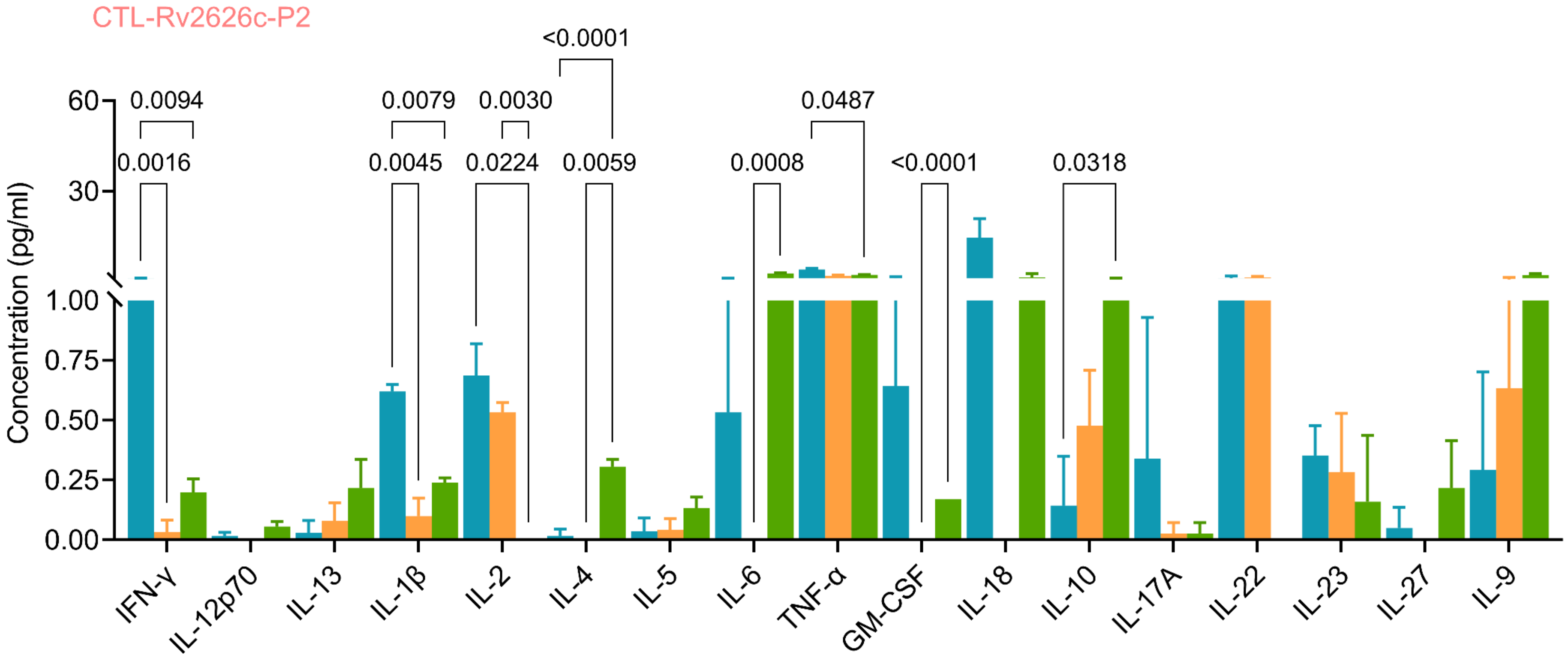
CTL-Rv2626c-P2
Concentration (pg/ml)
60
30
1.00
0.75
0.50
0.25
0.00
0.0094
0.0016
0.0079
0.0045
0.0030
0.0224
<0.0001
0.0059
0.0008
0.0487
<0.0001
0.0318
IFN-γ
IL-12p70
IL-13
IL-1β
IL-2
IL-4
IL-5
IL-6
TNF-α
GM-CSF
IL-18
IL-10
IL-17A
IL-22
IL-23
IL-27
IL-9

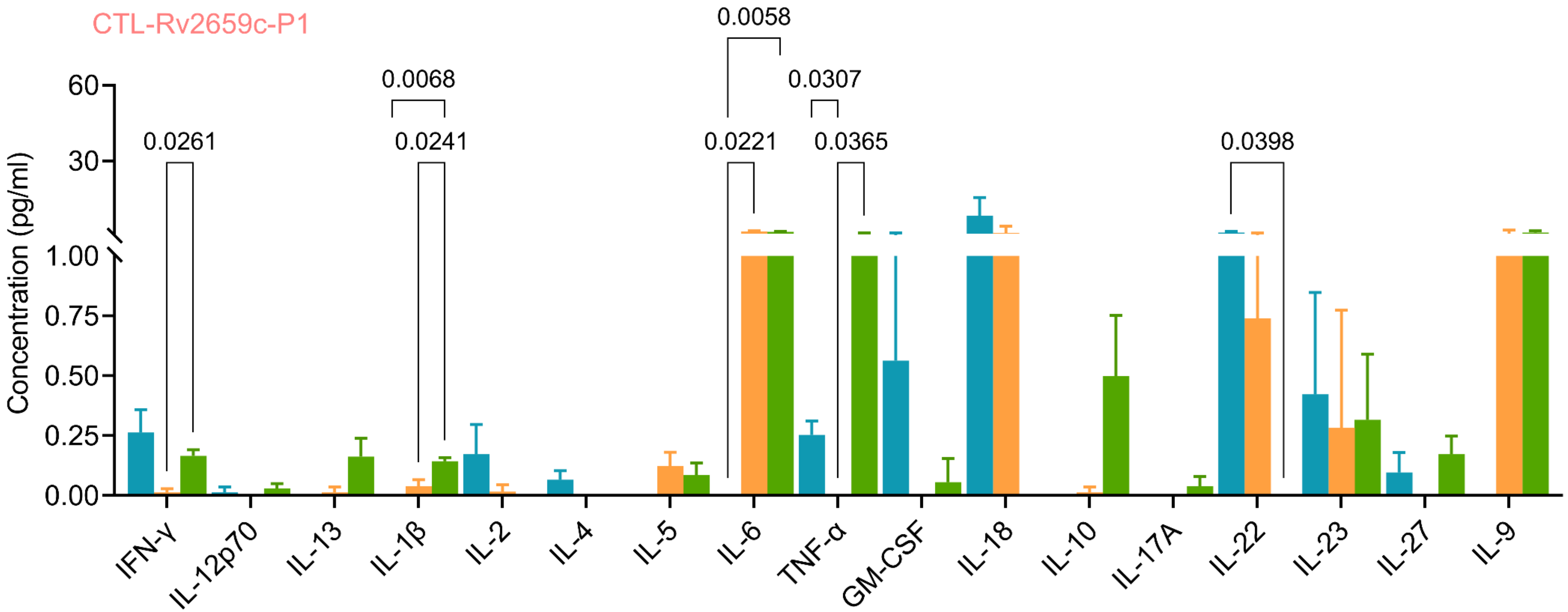
CTL-Rv2659c-P1
Concentration (pg/ml)
60
30
1.00
0.75
0.50
0.25
0.00
0.0261
0.0068
0.0241
0.0058
0.0221
0.0307
0.0365
0.0398
IFN-γ
IL-12p70
IL-13
IL-1β
IL-2
IL-4
IL-5
IL-6
TNF-α
GM-CSF
IL-18
IL-10
IL-17A
IL-22
IL-23
IL-27
IL-9

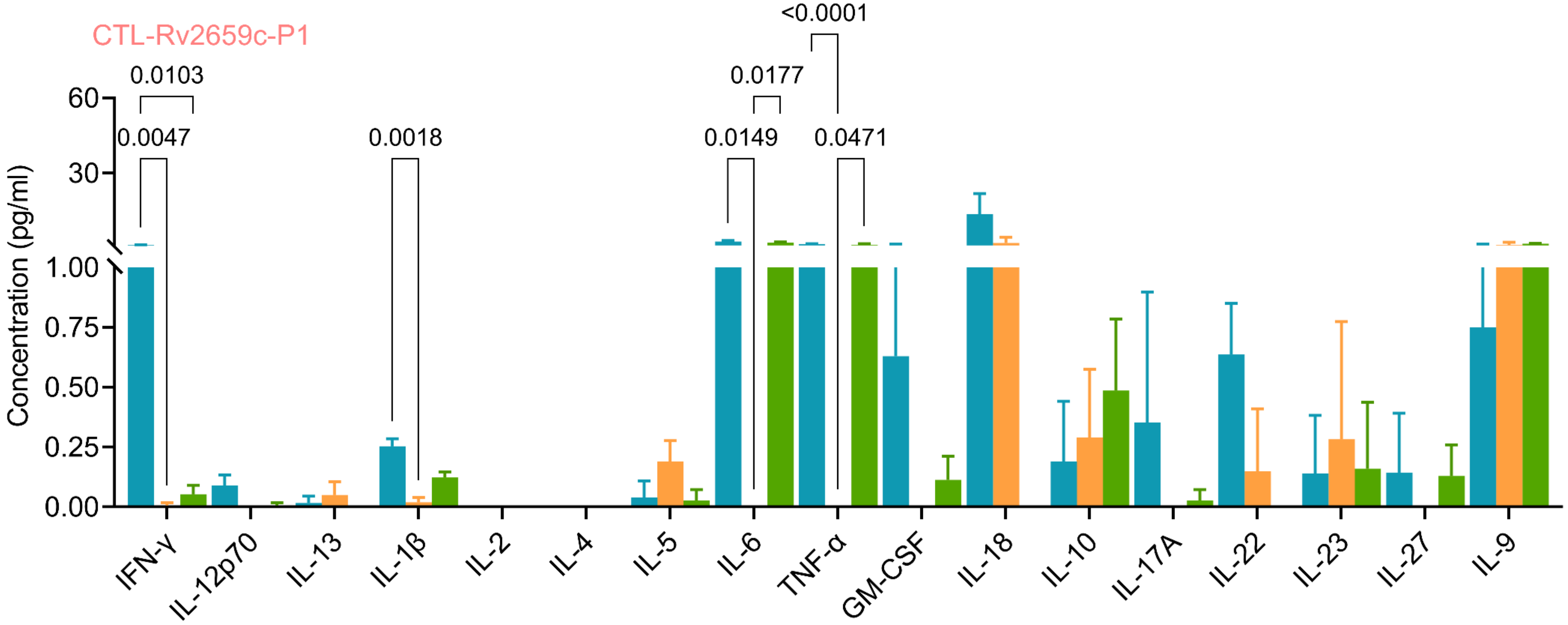
CTL-Rv2659c-P1
Concentration (pg/ml)
60
30
1.00
0.75
0.50
0.25
0.00
0.0103
0.0047
0.0018
0.0177
0.0149
<0.0001
0.0471
IFN-γ
IL-12p70
IL-13
IL-1β
IL-2
IL-4
IL-5
IL-6
TNF-α
GM-CSF
IL-18
IL-10
IL-17A
IL-22
IL-23
IL-27
IL-9

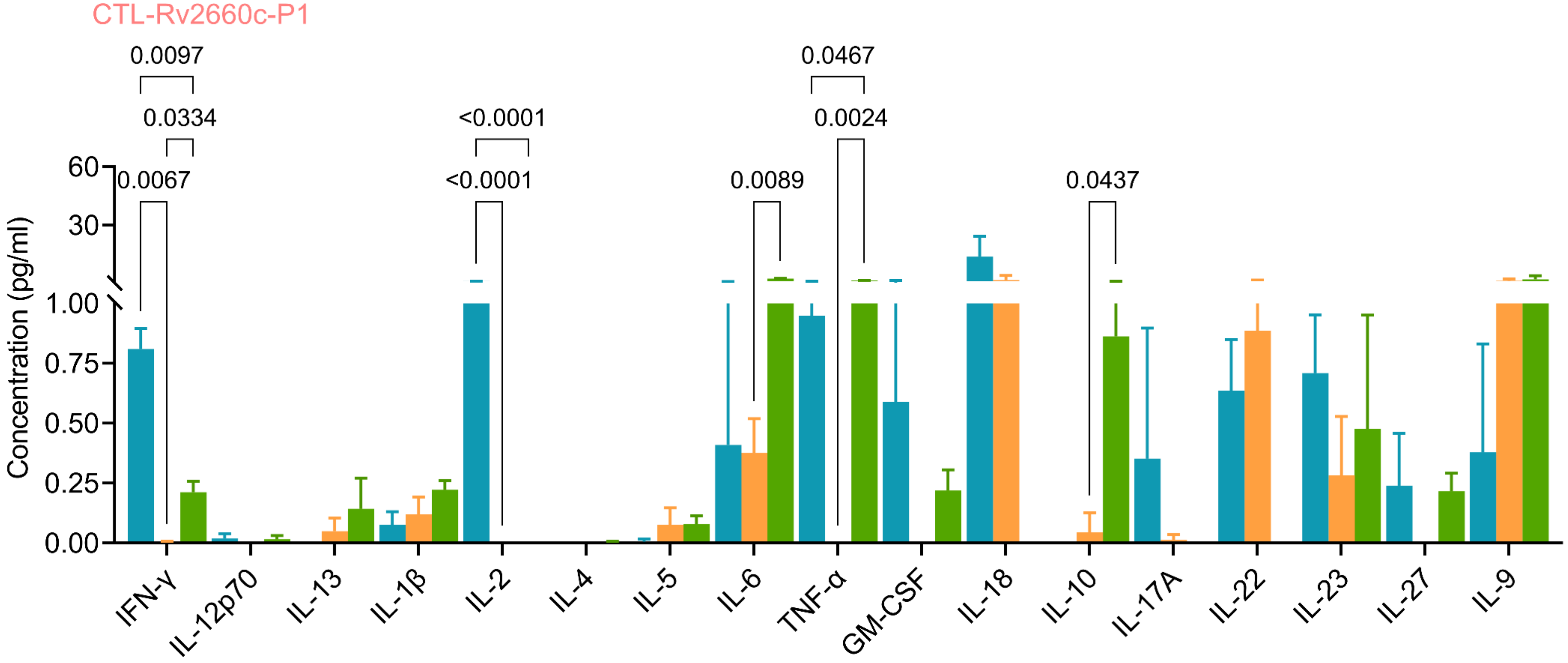

CTL-Rv2660c-P1
0.0097
0.0334
0.0067
<0.0001
<0.0001
0.0467
0.0024
0.0089
0.0437
Concentration (pg/ml)
60
30
1.00
0.75
0.50
0.25
0.00
IFN-γ
IL-12p70
IL-13
IL-1β
IL-2
IL-4
IL-5
IL-6
TNF-α
GM-CSF
IL-18
IL-10
IL-17A
IL-22
IL-23
IL-27
IL-9

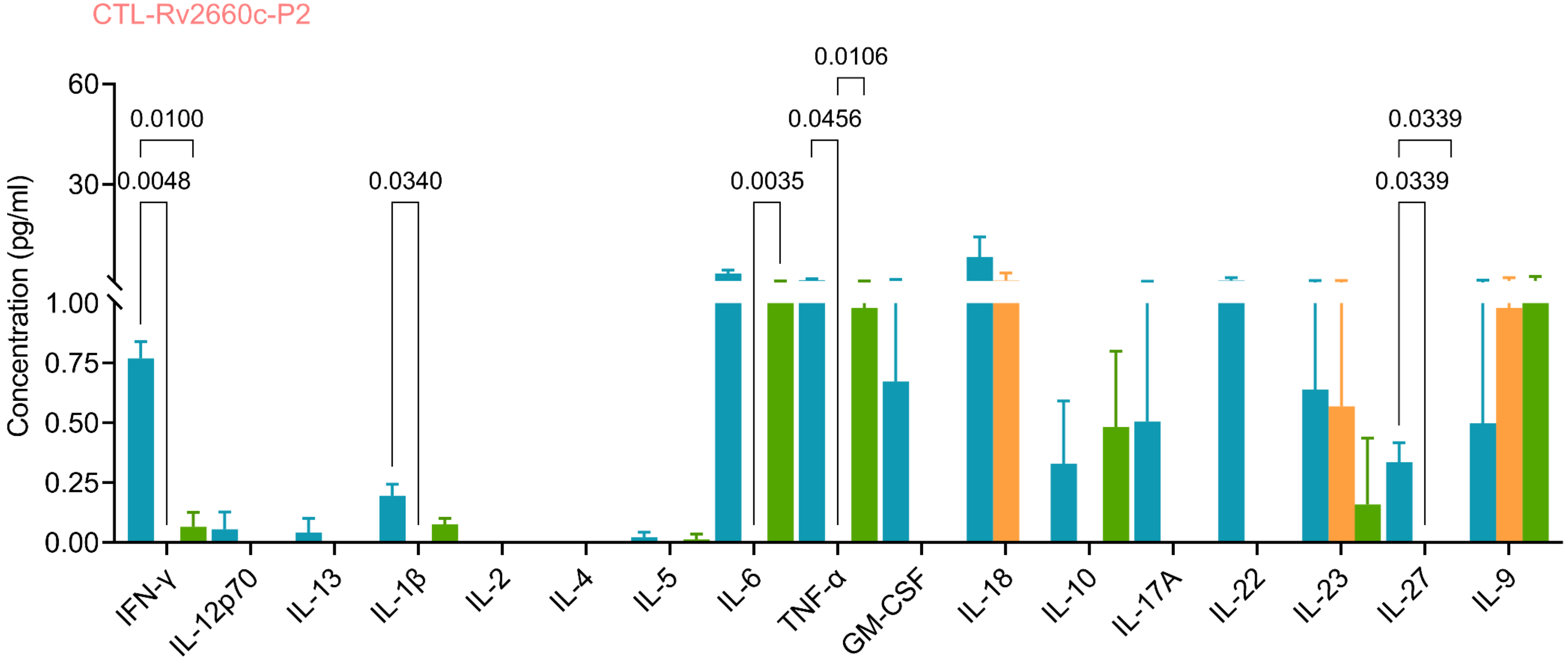

Figure S2. Detailed information of cytokines induced by CTL dominant peptides in mice. The splenocytes collected from mice in ATB, LTBI, and UC groups were stimulated with the ten CTL dominant peptides for 48 hours. The levels of IFN-γ, IL-12p70, IL-13, IL-1β, IL-2, IL-4, IL-5, IL-6, TNF-α, GM-CSF, IL-18, IL-10, L-17A, IL-22, IL-23, IL-27, and IL-9 cytokines in the supernatant were detected with a Mouse Th1/Th2/Th9/Th17/Th22/Treg Cytokine Kit. The cytokine differences among the three

groups were compared with the two-way analysis of variance (ANOVA) corrected with the Tukey test. All data were shown as mean + SEM ($n = 3$). $P < 0.05$ was considered significantly different.

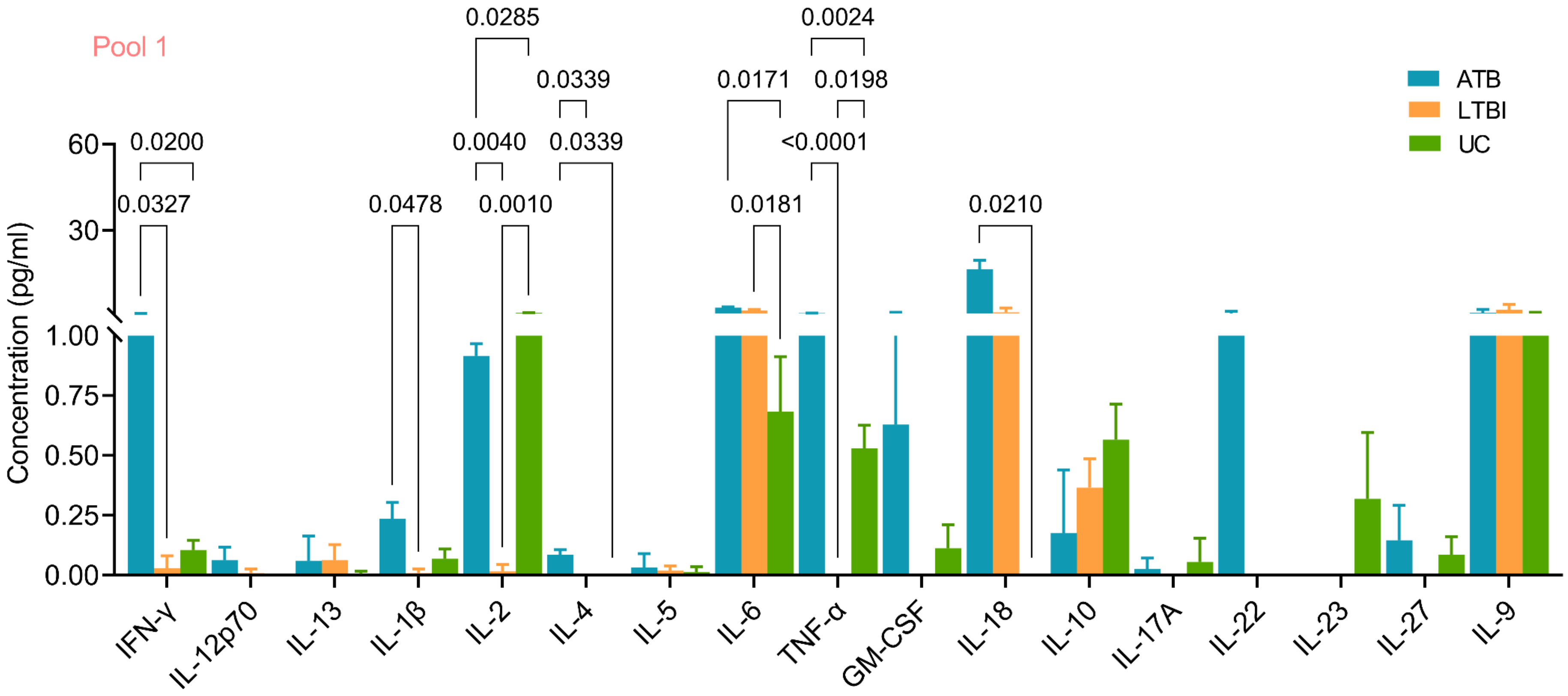

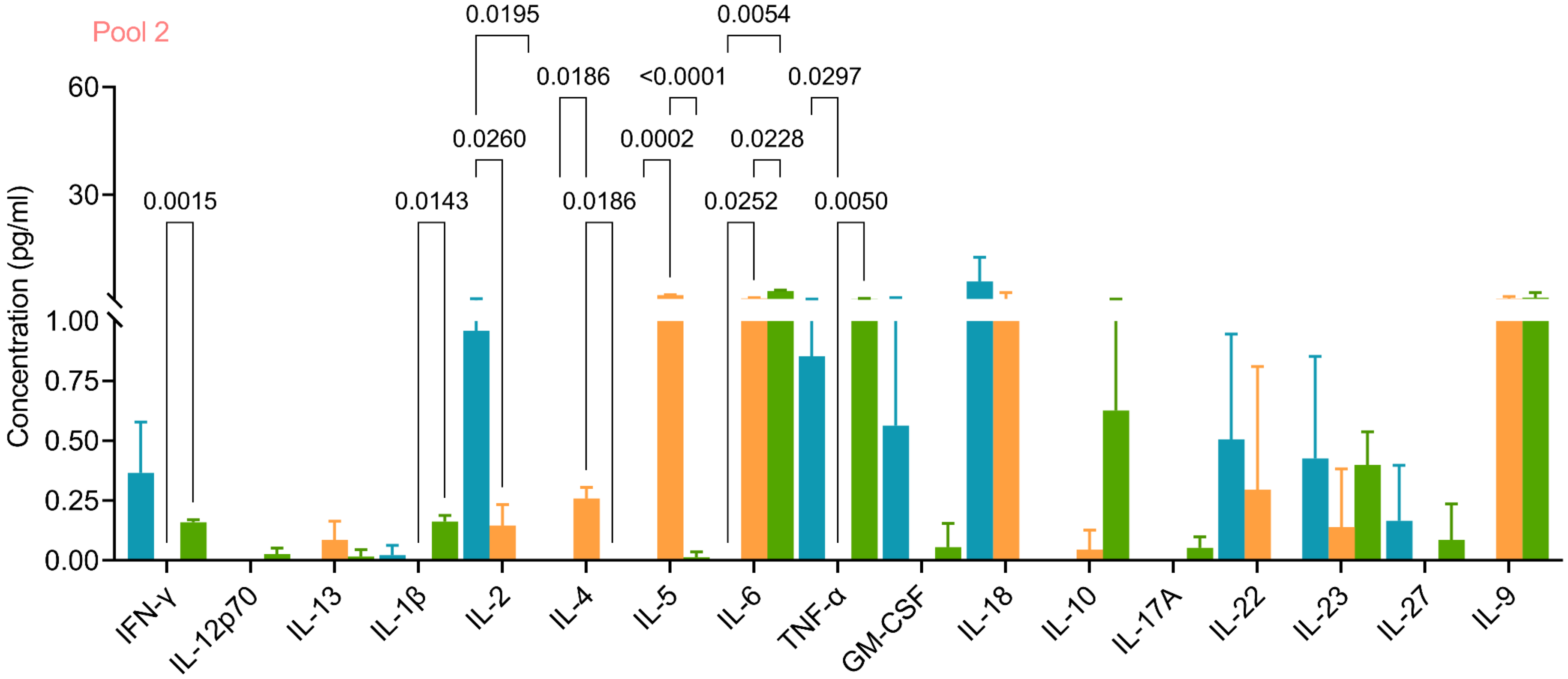
Pool 2
Concentration (pg/ml)
60
30
1.00
0.75
0.50
0.25
0.00
0.0015
0.0143
0.0260
0.0195
0.0186
0.0186
0.0002
<0.0001
0.0054
0.0252
0.0228
0.0297
0.0050
IFN-γ
IL-12p70
IL-13
IL-1β
IL-2
IL-4
IL-5
IL-6
TNF-α
GM-CSF
IL-18
IL-10
IL-17A
IL-22
IL-23
IL-27
IL-9

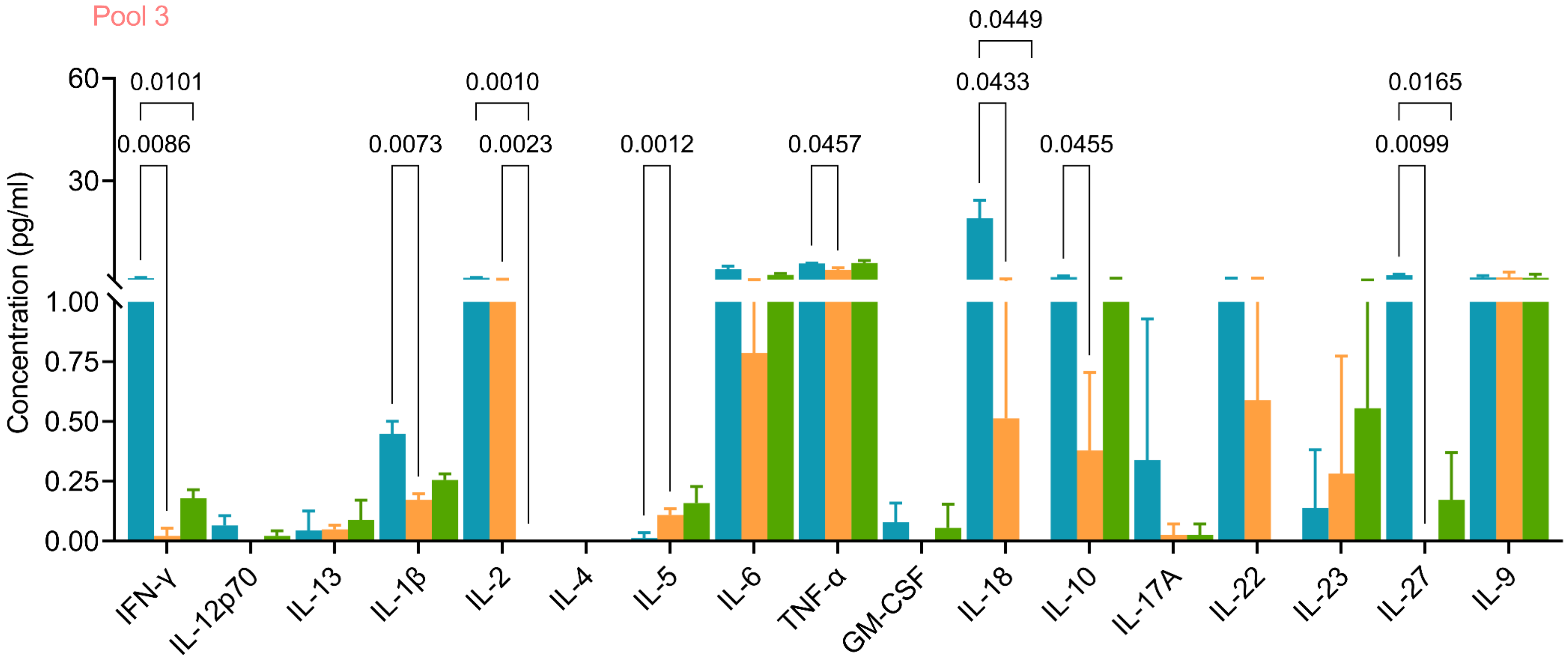

Pool 3
Concentration (pg/ml)
60
30
1.00
0.75
0.50
0.25
0.00
0.0101
0.0086
0.0073
0.0010
0.0023
0.0012
0.0457
0.0449
0.0433
0.0455
0.0165
0.0099
IFN-γ
IL-12p70
IL-13
IL-1β
IL-2
IL-4
IL-5
IL-6
TNF-α
GM-CSF
IL-18
IL-10
IL-17A
IL-22
IL-23
IL-27
IL-9

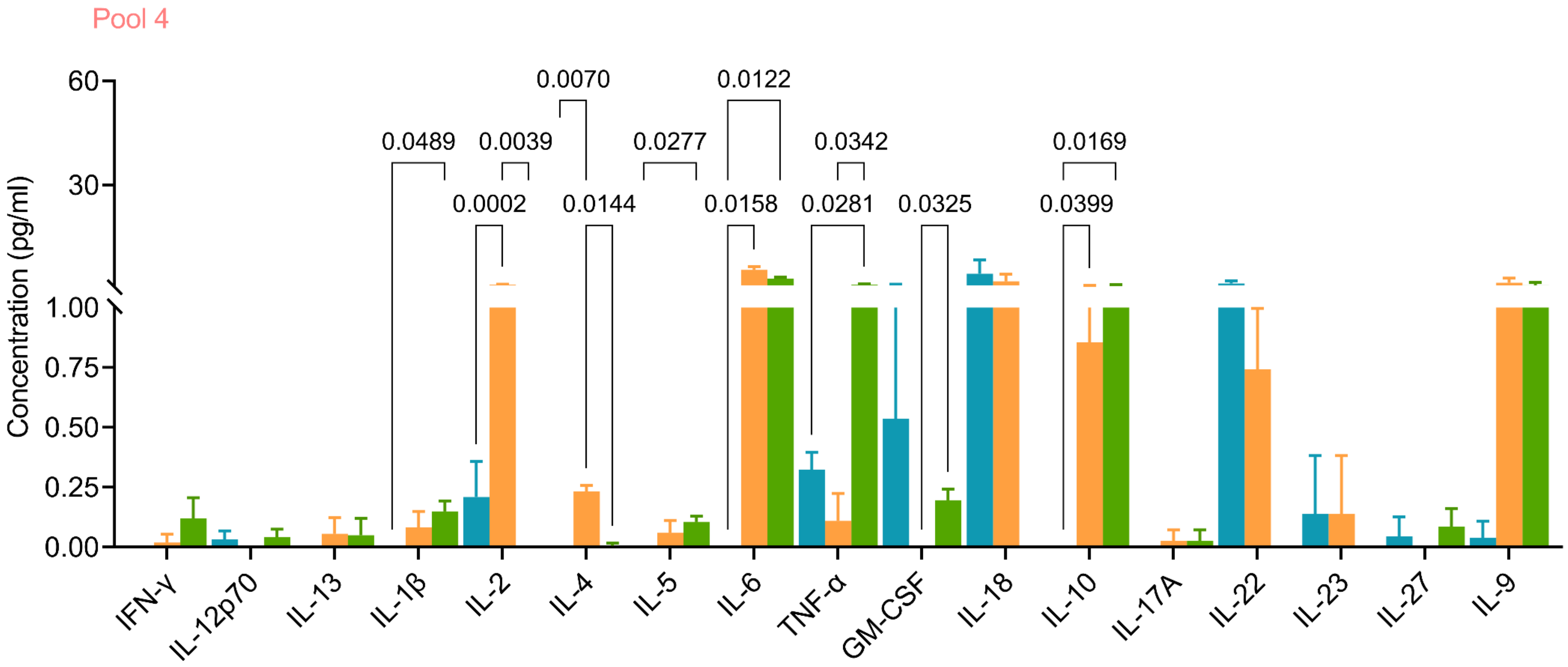
Pool 4
Concentration (pg/ml)
60
30
1.00
0.75
0.50
0.25
0.00
0.0070
0.0122
0.0489
0.0039
0.0277
0.0342
0.0169
0.0002
0.0144
0.0158
0.0281
0.0325
0.0399
IFN-γ
IL-12p70
IL-13
IL-1β
IL-2
IL-4
IL-5
IL-6
TNF-α
GM-CSF
IL-18
IL-10
IL-17A
IL-22
IL-23
IL-27
IL-9

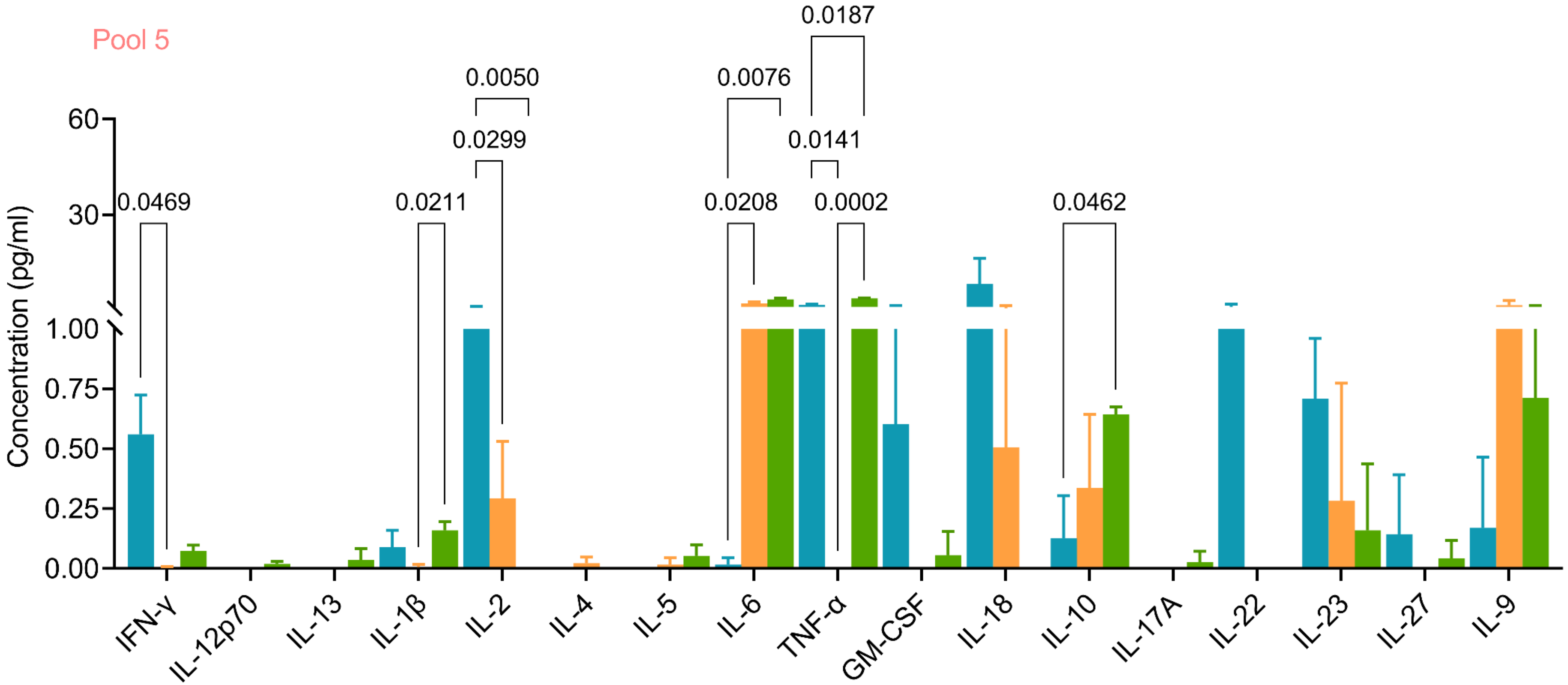

Figure S3. Detailed information on cytokines induced by peptide pools in mice. The splenocytes collected from mice in ATB, LTBI, and UC groups were stimulated with the five peptides pools for 48 hours. The levels of IFN-γ, IL-12p70, IL-13, IL-1β, IL-2, IL-4, IL-5, IL-6, TNF-α, GM-CSF, IL-18, IL-10, L-17A, IL-22, IL-23, IL-27, and IL-9 cytokines in the supernatant were detected with a Mouse Th1/Th2/Th9/Th17/Th22/Treg Cytokine Kit. The cytokine differences among the three groups were compared with

the two-way analysis of variance (ANOVA) corrected with the Tukey test. All data were shown as mean + SEM ($n = 3$). $P < 0.05$ was considered significantly different.
